# Why Does One Measure Resonance Raman Optical Activity? A Unique Case of Measurements under Strong Resonance versus Far-from-Resonance Conditions

**DOI:** 10.1021/acs.jpclett.4c00270

**Published:** 2024-04-29

**Authors:** Ewa Machalska, Monika Halat, Takumi Tani, Tomotsumi Fujisawa, Masashi Unno, Andrzej Kudelski, Malgorzata Baranska, Grzegorz Zając

**Affiliations:** 1 Jagiellonian Centre for Experimental Therapeutics (JCET), Jagiellonian University, Bobrzynskiego 14, 30-348 Krakow, Poland; 2 Laboratory for Spectroscopy, Molecular Modeling and Structure Determination, Institute of Nuclear Chemistry and Technology, Dorodna 16, 03-195 Warsaw, Poland; 3 Department of Plant Biology and Biotechnology, Faculty of Biotechnology and Horticulture, University of Agriculture, Al. Mickiewicza 21, 31-120 Krakow, Poland; 4 Department of Chemistry and Applied Chemistry, Faculty of Science and Engineering, Saga University, Saga 840-8502, Japan; 5 Faculty of Chemistry, University of Warsaw, Ludwika Pasteura 1, 02-093 Warsaw, Poland; 6 Faculty of Chemistry, Jagiellonian University, Gronostajowa 2, 30-387 Krakow, Poland

## Abstract

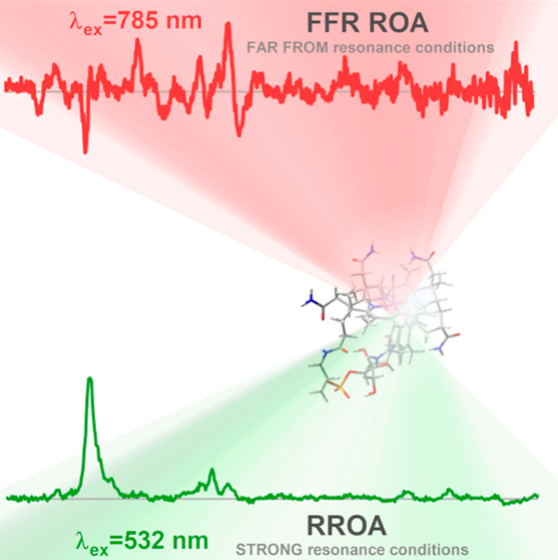

Raman optical activity (ROA) spectroscopy exhibits significant potential in the study of (bio)molecules as it encodes information on their molecular structure, chirality, and conformations. Furthermore, the method reveals details on excited electronic states when applied under resonance conditions. Here, we present a combined study of the far from resonance (FFR)-ROA and resonance ROA (RROA) of a single relatively small molecular system. Notably, this study is the first to employ the density functional theory (DFT) analysis of both FFR-ROA and RROA spectra. This is illustrated for cobalamin derivatives using near-infrared and visible light excitation. Although the commonly observed monosignate RROA spectra lose additional information visible in bisignate nonresonance ROA spectra, the RROA technique acts as a complement to nonresonance ROA spectroscopy. In particular, the combination of these methods integrated with DFT calculations can reveal a complete spectral picture of the structural and conformational differences between tested compounds.

Resonance Raman (RR) spectroscopy has been proven to be a potent technique for investigating the molecular structure and electronic properties of organometallic vitamin B_12_ and its analogues. Due to its electronic absorption in the visible range, related to the corrin ring-based π → π* transitions,^1−3^ the vibrational Raman intensities of the corrinoid modes can be significantly enhanced by blue or green laser lines. This property was demonstrated for the first time in the early 1970s when Mayer and co-workers measured the RR spectra of native vitamin B_12_ and several other synthetic corrinoids dissolved in organic and inorganic solvents using 514.5 nm laser excitation.^4−6^ Later, due to its unique abilities, Raman spectroscopy was used to investigate Co-alkyl modes^7^ and explore the conformational changes of the corrin macrocycle upon the binding of B_12_ coenzymes^8,9^ and under various Co oxidation states.^10^

Recently, resonance Raman optical activity (RROA) has played a notable role in the structural analysis of vitamin B_12_ and its derivatives.^11,12^ As a chiral version of RR spectroscopy, RROA measures the intensity difference in the Raman scattering of right- and left-circularly polarized (RCP and LCP, respectively) light,^13,14^ yielding bi- or monosignate patterns depending on the resonance conditions. Far from resonance (FFR)-ROA or RROA spectra with multiple electronic states may possess both positive and negative bands. On the other hand, RROA should be monosignate in the single electronic state (SES) limit, where the excitation wavelength is in resonance with one electronic transition.^15,16^ Studies have already shown that RROA combined with electronic circular dichroism (ECD) enables the exploration of subtle alterations in the structure of truncated vitamin B_12_ analogues^12^ and derivatives with different upper axial substituents or ring modifications.^11,17^ However, the RROA spectra can be significantly influenced by the so-called ECD-Raman (eCP-Raman) effect, arising from a combination of ECD and circularly polarized Raman (CP-Raman) spectroscopy.^17−19^ Nevertheless, the ECD-Raman effect can be controlled, minimized, and subtracted if necessary.^19^

Up to now, bisignate or monosignate RROA spectral patterns have been proven to be beneficial in the study of varied molecular systems, including carotenoid aggregates,^14,20−24^ metal complexes,^18,25,26^ photoreceptor proteins,^16,27,28^ and drugs.^29^ The sign and magnitude of RROA bands in the SES limit are determined by the ECD resonant transition,^15^ where the spectral pattern is similar to that of the parent RR spectrum (except for the sign). Therefore, to support ROA measurements collected under strong resonance (SR) conditions (532 nm), FFR-ROA spectroscopy (785 nm) combined with computational studies is necessary to conduct a complete structural and conformational analysis. Several ROA measurements in resonance and FFR modulations of metal complexes using solely a 532 nm excitation line have recently been reported.^25^ Moreover, near-infrared (NIR) excitation at 785 nm has been used to obtain pure ROA spectra of europium complexes, which was not possible under visible (vis) 532 nm incident light, where the luminescence of europium dominates.^30^

Herein, we employed the rarely implemented combination of ROA and RROA to investigate cyanocobalamin species using vis and NIR incident radiation via commercial and home-built ROA setups, respectively.^16,31,32^ These methods usually require different sample concentrations, that is, diluted conditions for RROA and concentrated ones in the case of FFR-ROA (Supporting Information). Despite different experimental modulations, this combined approach allows us to investigate a single molecular system under different resonance conditions.

We examined four cyanocobalamin analogues, namely, (CN)Cbl (**Cbl-1**), (CN)Cbl(*c*-lactone) (**Cbl-2**), (CN)13-*epi*-Cbl(*e*-lactone) (**Cbl-3**), and (CN)13-*epi*-Cbl(*e*-CO_2_Me)(13-OH) (**Cbl-4**); the molecular structures of the studied compounds are presented in Figure 1. As shown in Figure 2, the UV–vis absorption spectra exhibit a set of characteristic bands for cobalamins, including the dominant so-called γ absorption band, located in the near-UV spectral range centered at ∼360 nm, and the one (**Cbl-3**, **Cbl-4**) or two (**Cbl-1**, **Cbl-2**) bands occurring in the α/β visible region, with the more intense counterpart located at ∼550 nm and the less intense one appearing at 521 nm. Thus, the “γ-band-type” transition is attributed to transitions with a predominantly corrin π → π* character, whereas the α/β ones correspond to the HOMO → LUMO electronic excitation of the corrin macrocycle.^1,10^

**Figure 1:**
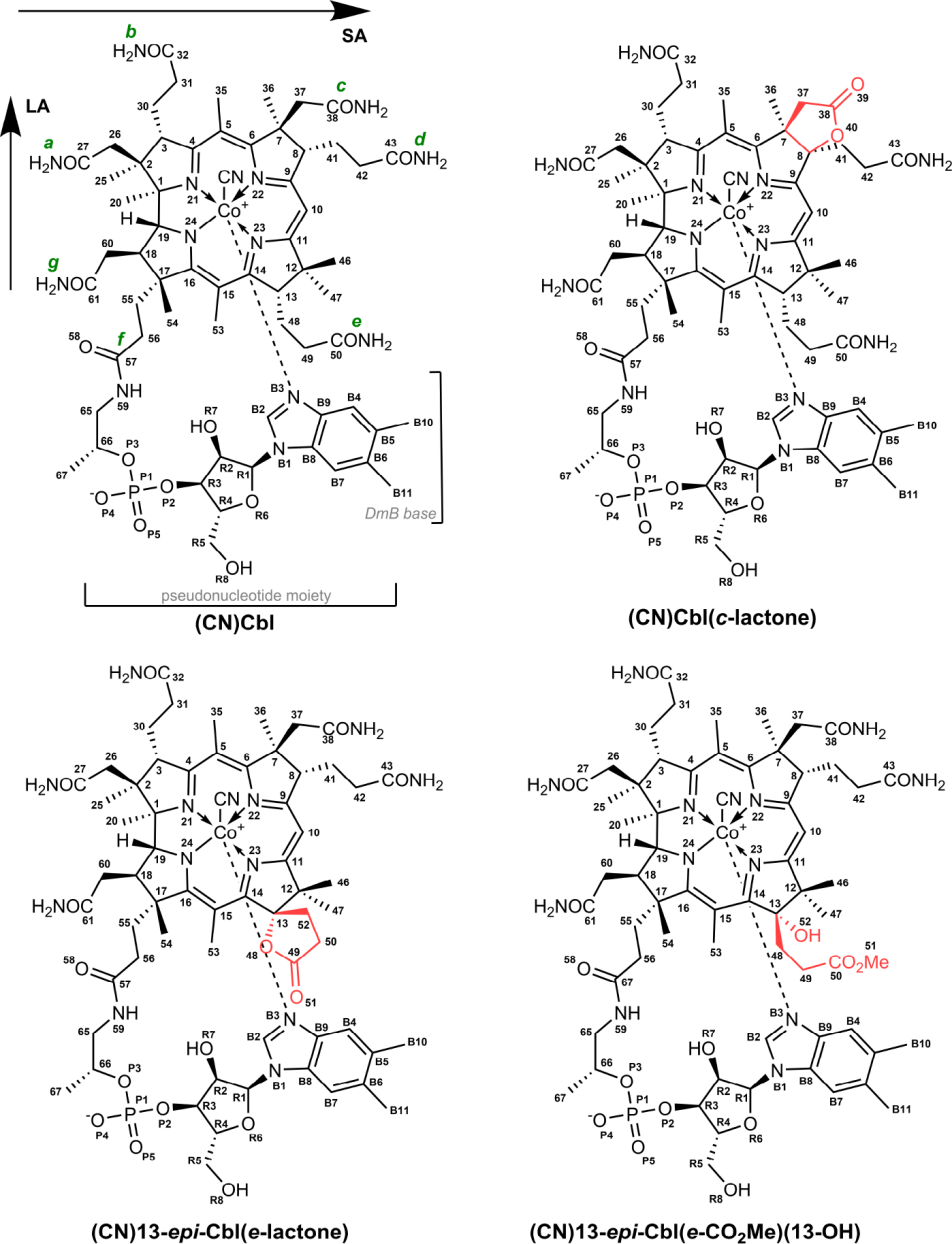
Molecular structures of the studied vitamin B_12_ analogues. Modifications to the native vitamin B_12_ structure are marked in red, while the amide chains of vitamin B_12_ are marked in green.

**Figure 2:**
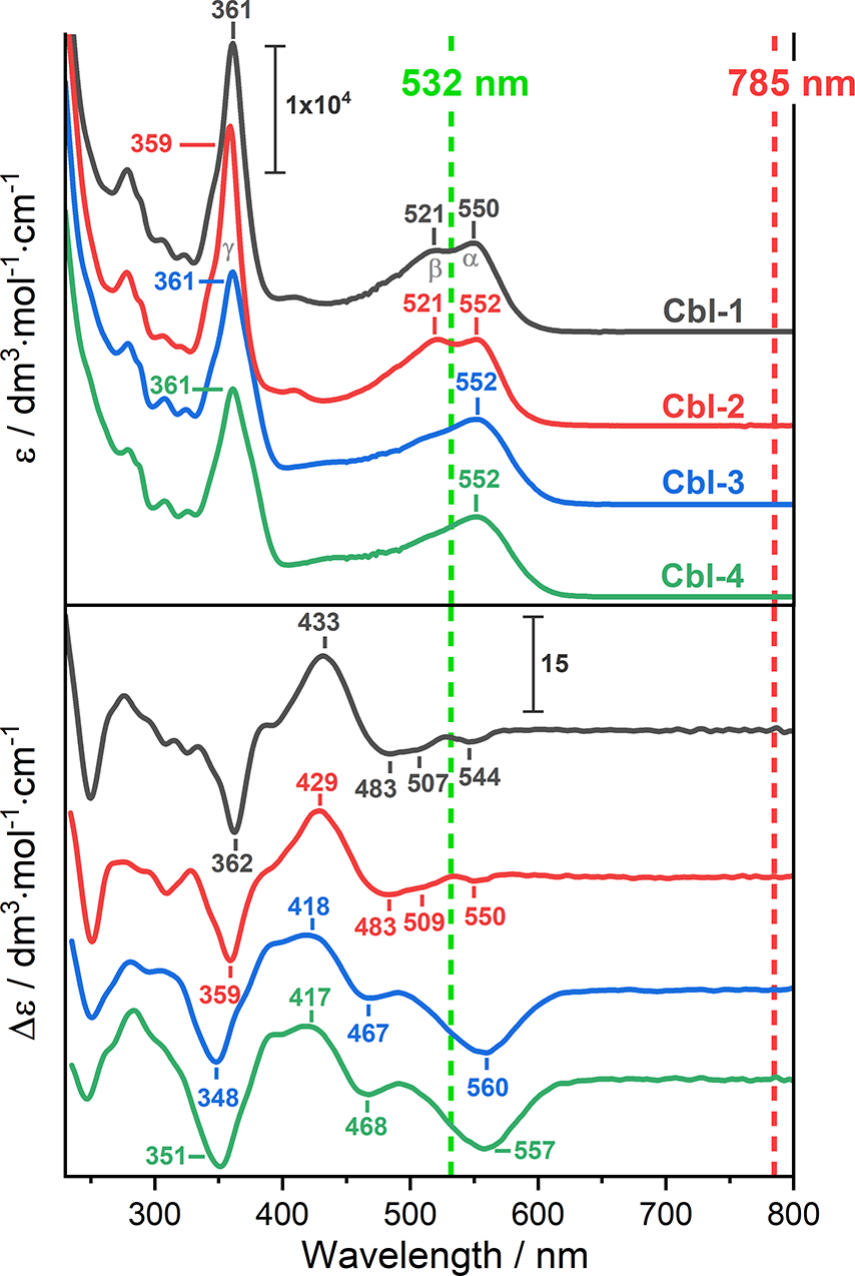
Comparison of experimental UV–vis (upper panel) and ECD (lower panel) spectra of the studied vitamin B_12_ analogues. The green and red dotted lines indicate the excitation wavelengths of 532 and 785 nm, respectively. The α, β, and γ absorption bands are marked in gray in the UV–vis spectrum of unmodified vitamin B_12_.

The modifications to the corrin macrocyclic side chains do not perturb the electronic structure to the extent observed for the upper axial ligand substitutions.^11^ Specifically, the spectral signatures of cobalamin molecules functionalized at the C13 position (**Cbl-3**, **Cbl-4**) of the corrin ring (Figure 1) exhibit almost the same absorption features, though they still preserve some differences compared to the native form (**Cbl-1**) and **Cbl-2**. Further, regarding the **Cbl-2** analogue (lactone modification at the *c* conjugation side), both the UV–vis and ECD signals display similar profiles to those observed for its unmodified counterpart (**Cbl-1**). However, some spectral differences are present in the ECD spectra; that is, **Cbl-3** and **Cbl-4** exhibit significantly different ECD spectral signatures compared to **Cbl-1** and **Cbl-2**. Moreover, based on the UV–vis and ECD spectra, the molecules studied herein can be classified into two groups: (**Cbl-1**, **Cbl-2**) and (**Cbl-3**, **Cbl-4**).

Figure 3 shows the experimental Raman spectra collected using 532 and 785 nm laser lines. The vibrational spectra of the cobalamin analogues reveal numerous and well-resolved spectral bands. Nevertheless, many of the observed Raman and ROA bands are composed of several normal modes (Tables S2 and S3, Supporting Information). This implies that the vibrational assignment of the cobalamin species is not simple without the use of isotope editing experiments. We demonstrated, however, that a successful assignment of many bands is possible by comparing the spectra obtained at different excitation wavelengths since the observed excitation wavelength dependence is well explained by density functional theory (DFT) simulations (Figures 3 and 4); the details of the DFT calculations and assignments are described later.

**Figure 3:**
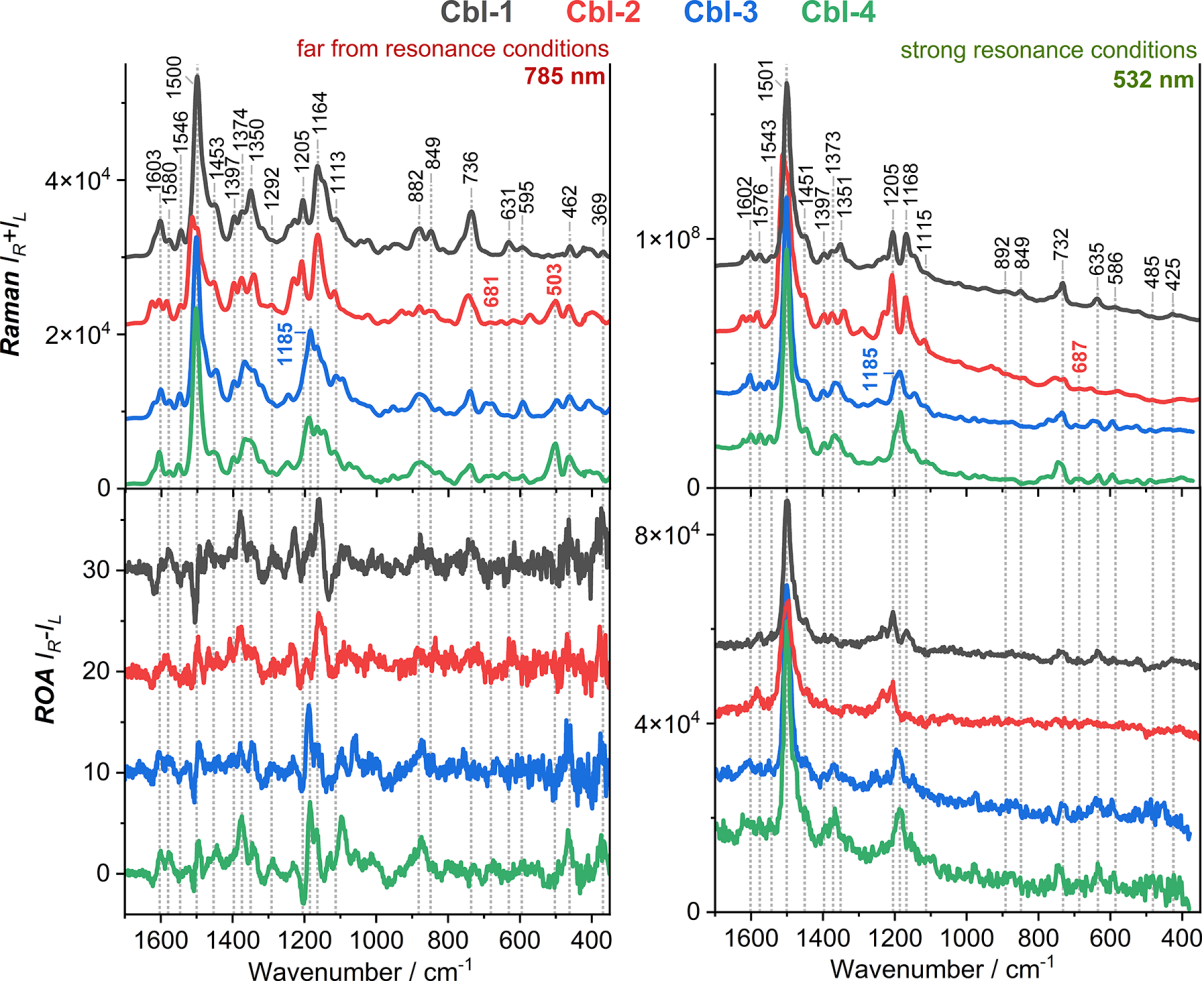
Comparison of experimental Raman (upper panel) and ROA (lower panel) spectra of the studied vitamin B_12_ analogues obtained at excitation wavelengths of 532 and 785 nm.

**Figure 4:**
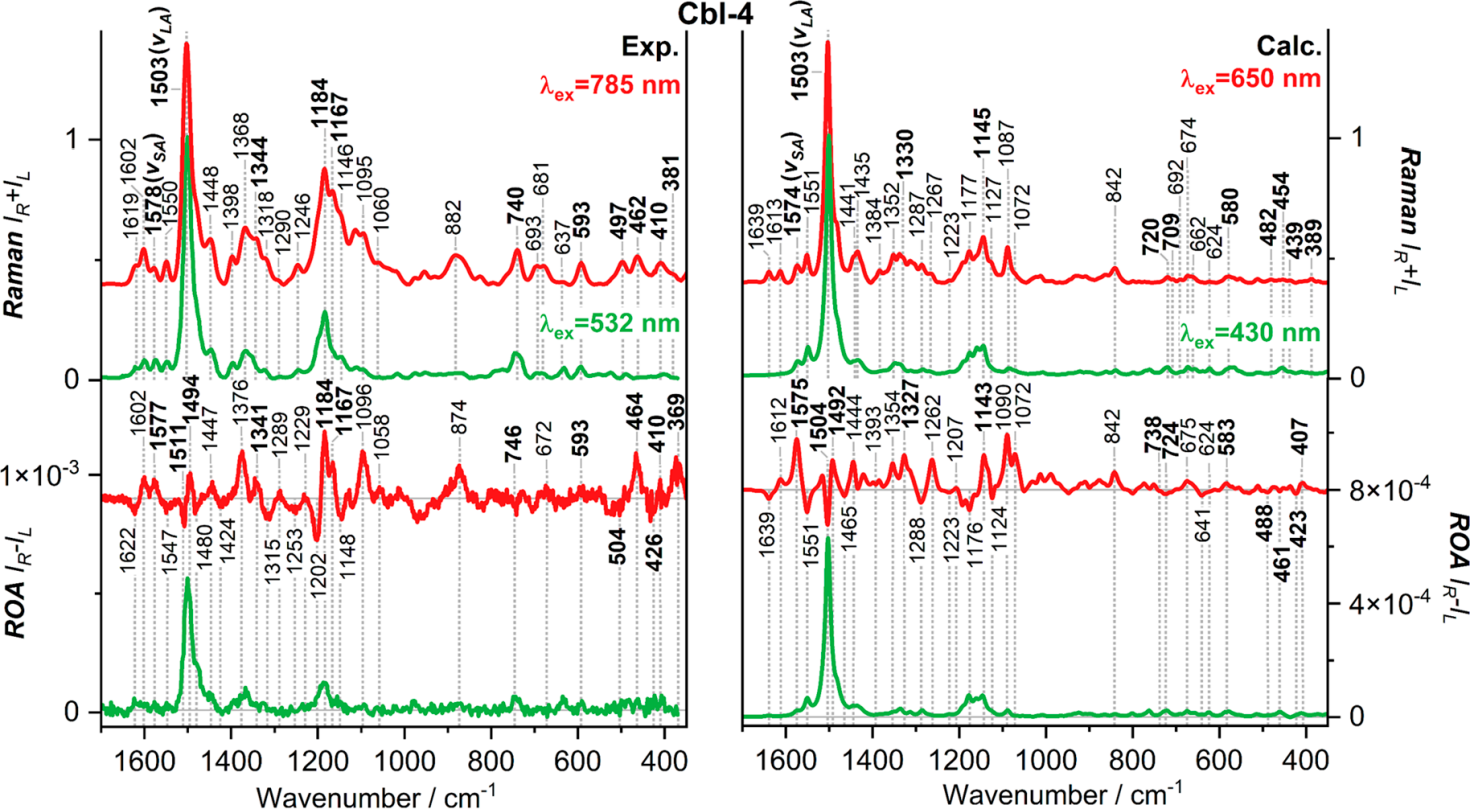
Comparison of the experimental (left panel) and calculated (right panel) Raman and ROA spectra of **Cbl-4**. CAM-B3LYP-GD3/6-31G(d)/MDF10/PCM theory level and Boltzmann-averaging (Δ*G*) of the lowest energy conformer spectra were used for the calculated spectra. The experimental spectra are obtained with excitation wavelengths of 532 and 785 nm, while the calculated spectra employ excitation wavelengths of 430 and 650 nm. The calculated vibrational frequencies are scaled by a factor of 0.941 (785 nm) and 0.940 (532 nm). Spectra normalized preserving ROA/Raman ratios. The most important bands discussed in the text were highlighted.

The most intense Raman band (∼1500 cm^–1^) is attributed to the long-axis polarized corrin macrocycle vibrations (*v*_LA_) and is mostly responsible for the in-phase C=C and C=N stretching modes along the longer axis (C5–Co–C15) of the corrin ring (Figure 1).^1,3,7,10^ Further, the relatively weak band in the range of ∼1540–1550 cm^–1^ is assigned to the short-axis polarized corrin ring motion (*v*_SA_) related to the C=C and C=N stretching modes along the shorter axis (Co–C10).^7^ Our DFT analysis also indicates that the spectral range of 1300–1400 cm^–1^ includes an abundance of CH_3_ and CH_2_ bending vibrations, while the 1100–1300 cm^–1^ region includes CH_2_ twisting and wagging, CH bending, and the corrin ligand C–C and C–N stretching modes (Tables S2 and S3, Supporting Information). In addition, the lower frequency region, below 500 cm^–1^, is distinctive for Co–C≡N stretching and bending vibrations.

Although these spectral features are common to the RR and FFR-Raman spectra, there is some variation between the two cases (Figure 3). Most notably, the RR signals are influenced to a significantly greater extent by macrocycle vibrations than the FFR-Raman ones. This finding is reasonable because the laser line at 532 nm is close in energy to the α-band absorption feature arising from the corrin π → π* transition polarized along the *v*_LA_ axis (Figure 2). Generally, the excitation at 532 nm predominantly enhances RR bands at ∼1500–1515, ∼1200, and ∼1160 cm^–1^ ranges (Figure 3), involving the vibrational modes of the corrin ring and peripheral functional groups (Tables S2 and S3, Supporting Information).

On the other hand, the measurements involving NIR radiation avoid electronic excitations of the corrin ring and, thus, the resonance conditions. The FFR-Raman spectra can sensitively detect not only corrin modes but also the vibrational motions of the 5,6-dimethylbenzimidazole (DmB) base and peripheral functional groups. This is mostly associated with the increase in the relative intensities of many other vibrational bands under FFR conditions compared to the resonance-enhanced signals in the RR spectra (∼1501, ∼1205, ∼1185, and ∼1168 cm^–1^).

As an example, the characteristic modes related to the bulky DmB group are included in the higher (1575–1580 cm^–1^) and lower (460–500 cm^–1^ or 700–780 cm^–1^) frequency regions. In the Raman spectra of all the **Cbl-*x*** species (*x* = 1, 2, 3, 4) obtained with the 785 nm laser line, the signals at ∼500 cm^–1^ (except for **Cbl-1**) and ∼465 cm^–1^ are higher in intensity relative to the *v*_LA_ mode. These bands are mainly assigned to the Co–C≡N bending, C≡N twisting, DmB ring stretching, and Co–N modes. Spectral signatures of **Cbl-*x*** also reveal the increase in the relative intensities of several bands due to CH_3_ vibrations, NH_2_ rocking in the side chains, and C–C and Co–N stretching vibrations under FFR conditions. These signals are included in the range of 700–785 cm^–1^ or 860–900 cm^–1^. Moreover, the bands at 430–480 cm^–1^ and 340–360 cm^–1^, which are mostly associated with the bending modes of Co–C≡N, CCN, and CNC, become more prominent and better separated under FFR conditions compared with the SR regime (Figure 3).

Figure 3 also illustrates the effect of ring modifications on the Raman spectra, with the functionalization at the C13 position or the modification of the corrin ring side chains leading to significant spectral variations. A few important changes under SR conditions can be noted. First, the RR spectra of **Cbl-3** and **Cbl-4** possess a rather prominent band centered at ∼1185 cm^–1^, while **Cbl-1** and **Cbl-2** give rise to two separate signals at ∼1205 and 1168 cm^–1^ due to CH_2_ wagging and twisting, C–H bending, CH_3_ rocking, and C–N and C–C stretching vibrations (Tables S2 and S3, Supporting Information). Second, the conjugation at the *c*-side chain of **Cbl-2** results in a significant upshift of the most intense RR band (1501 → 1514 cm^–1^) associated with motions of the corrin ring.

At the bottom of Figure 3, the ROA spectra recorded at laser excitation wavelengths of 532 and 785 nm are shown. This comparison reveals that in the RROA spectra, most of the vibrational bands have positive signs, while a few negative signals occur, particularly in the range of 1300–1400 cm^–1^ and 300–600 cm^–1^. On the other hand, the FFR-ROA spectra are bisignate over the entire vibrational range, providing richer structural information, often in the form of additional bands that are not clearly visible in the RROA spectra of the **Cbl-*x*** species. Specifically, some of the negative and positive bands at ∼1620(−), ∼1600(+), ∼1350(+), ∼1315(−), and ∼1290(+) are much better pronounced in the FFR-ROA spectra. Two higher-frequency bands are associated with corrin-ring stretching modes, while the remaining three are mainly attributed to the CH_2_ twisting and wagging, C–H bending, and DmB ring breathing modes (Tables S2 and S3, Supporting Information). Moreover, similar to its parent technique of Raman spectroscopy, FFR-ROA shows distinct lower-frequency bands (<400 cm^–1^), generally associated with Co–C≡N stretching vibrations (i.e., for **Cbl-1**: the negative–positive couplet at 497 and 466 cm^–1^, and the positive signal at 370 cm^–1^).

Although many differences are noticeable between the ROA spectra of **Cbl-1** and its modifications at an excitation of 785 nm, the overall spectral profiles are similar. This spectral similarity is especially evident in the range of 800–1600 cm^–1^. For instance, all investigated **Cbl-*x*** species show the negative band at ∼1620 cm^–1^ and the positive–negative couplet at ∼1505(−)/∼1490(+) cm^–1^ (with both features related mainly to stretching motions of the corrin ring), and a negative signal at ∼1315 cm^–1^ or two positive vibrational bands at ∼1160–1190 cm^–1^, assigned to CH_3_ and C–H bending, CH_2_ wagging and twisting, CH_3_ rocking, and C–C and C–N corrin-ring stretching motions.

Furthermore, both RR and RROA spectra are influenced more by the vibrational modes coupling to the α/β transition compared to the FFR Raman and ROA. The observed enhancement involving the contribution of multiple electronic states (e.g., cor-π, CN-π, DmB-π, and Co 3d orbitals) is well reproduced by our calculations (Figures S3 and S4). As has been described for **Cbl-1**,^11^ positive RROA signals arise from the resonance upon the corrin macrocycle π → π* transitions, associated with negative ECD bands; these are located at 544 and 507 nm in the **Cbl-1** spectrum or at 550 and 509 nm in the case of **Cbl-2** (Figure 2). In turn, the negative RROA bands result from resonance involving the positive ECD signals at 433 nm for **Cbl-1** and 429 nm for **Cbl-2**.

As noted above, the DFT-calculated Raman and ROA spectra reproduce the experimental features rather well. As an example, Figure 4 compares the observed and simulated spectra for **Cbl-4** at CAM-B3LYP-GD3/6-31G(d)/MDF10/PCM theory level. **Cbl-x** are rather large molecular systems, composed of rigid corrin ring but also loose side chains and pseudonucleotide moiety. Therefore, we used here the CAM-B3LYP^33^ DFT functional combined with the Grimme’s Dispersion correction^34^ (GD3) to take into account the long-range intramolecular interactions of those parts of the molecule. Note that, we tested both B3LYP^35−38^ and CAM-B3LYP functional with and without GD3 corrections. A similarity analysis in several spectral ranges was performed to determine which theory level is the most sufficient for studied systems.^39,40^ The similarity analysis did not indicate which method is superior, however, CAM-B3LYP-GD3 was slightly better for Raman and preresonance ROA, while B3LYP for FFR ROA (Figures S7–S14, Supporting Information). Note that the excitation wavelengths of 430 and 650 nm were used here for the DFT calculations at the CAM-B3LYP-GD3 theory level. Although the experimental incident laser wavelengths are 532 and 785 nm, the theoretical electronic transition energies are blue-shifted compared to the experimental ones. Thus, the theoretical excitation wavelengths were adapted accordingly to mimic both preresonance and far-from resonance conditions.

The experimental FFR-ROA spectrum of **Cbl-4** displays bands due to the C=C and C=N stretching vibrations of the corrin ring and the CH_3_, CH_2_, and C–H bending motions of the macrocyclic side chains at ∼1622(−), ∼1602(+), ∼1577(+), ∼1547(−), ∼1511(−), and ∼1494(+) cm^–1^. These spectral patterns, including the associated signs, are well reproduced. Notably, intensities of the lower-frequency range (<1000 cm^–1^) in the calculated spectra are significantly lower than those in the experimental FFR-ROA and FFR-Raman spectra, even though the temperature correction was performed (Supporting Information), while the values of the circular intensity difference (CID, the ratio of ROA to Raman intensity) are comparable (Table S4, Supporting Information). It is probably due to the overestimation of the *v*_LA_ mode intensities in the calculated spectra.

Furthermore, the excitation line reveals a notable influence on the CID ratios. As shown earlier, the RROA spectral signals of **Cbl-*x*** are mostly positive in sign due to the resonance with the first electronic transition of **Cbl-*x***, characterized by a negative ECD band (∼550 nm), while some negative RROA bands may be related to the positive ECD band located at ∼430 nm. However, values of the CID ratio and the anisotropy *g*-factor (the quotient of ECD and UV–vis absorption intensities) usually do not obey the SES theory regime (CID = −0.5*g*),^15^ which is rather expected as the resonance here occurs via multiple electronic states (Table S5, Supporting Information). Notably, the CID ratios of the most bands due to the corrin ring have similar or higher absolute values in the resonance regime compared to FFR conditions. For example, the CID of the *v*_LA_ modes of **Cbl-1** is ∼4.2 × 10^–4^ for the resonance regime (1501 cm^–1^), while 1.3 × 10^–4^ and −2.9 × 10^–4^ under FFR conditions (1491 and 1504 cm^–1^, respectively). More notably, other bands from ∼1161–1168 cm^–1^ range exhibit CID ratios a few times higher under FFR conditions (5.6 × 10^–4^ at 1161 cm^–1^), than under resonance ones (2.3 × 10^–4^ at 1168 cm^–1^; Table S4, Supporting Information). In general, both RR and RROA intensities should be enhanced compared to the FFR conditions, however, this is not the case for CID values, which are not necessarily larger in resonance conditions. What is more, in the resonance via multiple electronic states, characterized by both positive and negative ECD and different *g*-factor values, some of the opposite RROA bands may be compensated due to the influence of electronic transitions with opposite ECD, and thus lead to lower CIDs.

The ROA measurements conducted herein by means of two different laser excitations for a single molecular system are notably challenging. This is because SR can result in many complications, namely, sample overheating, laser-induced decomposition, the ECD-Raman effect, or fluorescence. On the other hand, the FFR settings typically require highly concentrated samples and a long accumulation time. However, the unique molecular structure and physical properties of the **Cbl-*x*** analogues provide an appropriate basis for measurements of both SR and FFR-ROA effects. In addition, the interpretations and simulations of RROA spectra are more difficult compared with those of FFR-ROA spectra. The FFR-ROA can be calculated routinely, using commercially available software, while the RROA calculations are still limited. One can use preresonance methodology to model RROA spectra, as conducted herein and in previous studies; however, this involves a strong approximation, and the theoretical excitation line needs to be precisely adjusted to not coincide with the calculated electronic transitions. A few new approaches to calculate the RROA have recently been published.^25,30^

In summary, we measured the Raman and ROA spectra of relatively small chiral metal-containing systems under FFR and SR conditions. To date, this represents a very rare and direct example where ROA spectra are generated for one molecular system using two laser excitations (785 and 532 nm) under both FFR and SR conditions.^16,30^ The DFT calculations, based on simple theoretical models, successfully reproduced the experimental observations and also provided a foundation for interpreting Raman/ROA spectral signatures. The current results reveal that the resonance and FFR Raman and ROA spectra are complementary in probing structural alterations of the cobalamin macrocycle. This novel approach in chiroptical analysis can provide unique structural details about relevant light-absorbing biomolecules.

## Supporting Information Available

Computational and experimental details, band assignments, and comparison of the theoretical and experimental spectra (PDF)
