# Supplementary material for: Why Does One Measure Resonance Raman Optical Activity? A Unique Case of Measurements under Strong Resonance versus Far-from-Resonance Conditions
Source: J Phys Chem Lett. 2024 Apr 29;15(18):4913–9. doi: 10.1021/acs.jpclett.4c00270 (PMC11089565; doi:10.1021/acs.jpclett.4c00270)
Supplement: Supplementary file 1 — jz4c00270_si_003.pdf [file jz4c00270_si_003.pdf]

SUPPORTING INFORMATION

Why Does One Measure Resonance Raman Optical Activity? A Unique Case of Measurements Under Strong Resonance Versus Far-From-Resonance Conditions

Ewa Machalska,[1,2] Monika Halat,[3] Takumi Tani,[4] Tomotsumi Fujisawa,[4] Masashi Unno,[4] Andrzej Kudelski,[5] Malgorzata Baranska,[1,6] Grzegorz Zając*[1]*

[1] Jagiellonian Centre for Experimental Therapeutics (JCET), Jagiellonian University, Bobrzynskiego 14, 30-348 Krakow (Poland)

[2] Laboratory for Spectroscopy, Molecular Modeling and Structure Determination, Institute of Nuclear Chemistry and Technology, Dorodna 16, 03-195 Warsaw (Poland)

[3] Department of Plant Biology and Biotechnology, Faculty of Biotechnology and Horticulture, University of Agriculture, A. Mickiewicza 21, 31-120 Krakow (Poland)

[4] Department of Chemistry and Applied Chemistry, Faculty of Science and Engineering, Saga University, Saga 840-8502 (Japan)

[5] Faculty of Chemistry, University of Warsaw, Ludwika Pasteura 1, 02-093 Warsaw (Poland)

[6] Faculty of Chemistry, Jagiellonian University, Gronostajowa 2, 30-387 Krakow (Poland)

TABLE OF CONTENTS

**Experimental procedures**	S3
Materials	S3
Electronic Absorption (UV-Vis) and Electronic Circular Dichroism (ECD) measurements	S3
Raman and Raman optical activity (ROA) measurements	S3
**Table S1.** Set of Raman and ROA measurement parameters of studied compounds	S3
**Calculations**	S4
MD simulations	S4
Quantum chemical calculations	S4
Similarity index	S4
**Figure S1.** Comparison of experimental and calculated (Boltzmann average) UV-Vis, ECD spectra of Cbl-1, calculated at different theory levels	S5
**Figure S2.** Comparison of experimental and calculated (Boltzmann average) UV-Vis, ECD spectra of Cbl-4, calculated at different theory levels	S6
**Figure S3.** Comparison of experimental and calculated (Boltzmann average) RRaman and RROA spectra of Cbl-1, calculated at different theory levels	S7
**Figure S4.** Comparison of experimental and calculated (Boltzmann average) RRaman and RROA spectra of Cbl-4, calculated at different theory levels	S8
**Figure S5.** Comparison of experimental and calculated (Boltzmann average) FFR-Raman and FFR-ROA spectra of Cbl-1, calculated at different theory levels	S9
**Figure S6.** Comparison of experimental and calculated (Boltzmann average) FFR-Raman and FFR-ROA spectra of Cbl-4, calculated at different theory levels	S10
**Figure S7.** Similarity analysis for experimental FFR Raman/ROA spectra of Cbl-1 (λ_{ex}=785 nm) in the 1650-550 cm^{-1} range and calculated (Boltzmann average, ΔG) spectra	S11
**Figure S8.** Similarity analysis for experimental RRaman/RROA spectra of Cbl-1 (λ_{ex}=532 nm) in the 1650-550 cm^{-1} range and calculated (Boltzmann average, ΔG) spectra	S11
**Figure S9.** Similarity analysis for experimental FFR Raman/ROA spectra of Cbl-4 (λ_{ex}=785 nm) in the 1650-550 cm^{-1} range and calculated (Boltzmann average, ΔG) spectra	S12
**Figure S10.** Similarity analysis for experimental RRaman/RROA spectra of Cbl-4 (λ_{ex}=532 nm) in the 1650-550 cm^{-1} range and calculated (Boltzmann average, ΔG) spectra	S12
**Figure S11.** Similarity analysis for experimental FFR Raman/ROA spectra of Cbl-1 (λ_{ex}=785 nm) in different spectral ranges and calculated (Boltzmann average, ΔG) spectra	S13
**Figure S12.** Similarity analysis for experimental RRaman/RROA spectra of Cbl-1 (λ_{ex}=532 nm) in different spectral ranges and calculated (Boltzmann average, ΔG) spectra	S13
**Figure S13.** Similarity analysis for experimental FFR Raman/ROA spectra of Cbl-4 (λ_{ex}=785 nm) in different spectral ranges and calculated (Boltzmann average, ΔG) spectra	S14
**Figure S14.** Similarity analysis for experimental RRaman/RROA spectra of Cbl-4 (λ_{ex}=532 nm) in different spectral ranges and calculated (Boltzmann average, ΔG) spectra	S14
**Figure S15.** Comparison of the experimental and calculated Raman and ROA spectra of Cbl-1.	S15
**Table S2.** Calculated and experimental frequencies of Cbl-1	S16
**Figure S16.** Experimental FFR Raman/ROA and RRaman/RROA spectra of Cbl-1 in comparison with calculated (Boltzmann average, ΔG) spectra	S17
**Table S3.** Calculated and experimental frequencies of Cbl-4	S18
**Figure S17.** Experimental FFR Raman/ROA and RRaman/RROA spectra of Cbl-4 in comparison with calculated (Boltzmann average, ΔG) spectra	S19
**Table S4.** Circular intensity difference values, obtained from Cbl-1 and Cbl-4 experimental spectra	S20
**Table S5.** Dissymmetry factor values, obtained from Cbl-1 and Cbl-4 experimental spectra	S20
**Table S6.** The relative ΔE_{ZPE}, Gibbs free energies ΔG, and Boltzmann populations at 298 K of the most stable conformers of Cbl-1	S21
**Table S7.** The relative ΔE_{ZPE}, Gibbs free energies ΔG, and Boltzmann populations at 298 K of the most stable conformers of Cbl-4	S21
**Figure S18.** Aligned structures of Cbl-1 and Cbl-4 conformers	S21
**Figure S19.** Comparison of experimental and calculated UV-Vis and ECD spectra of Cbl-1 conformers, calculated at CAM-B3LYP-GD3/6-31G(d)/MDF10/PCM level	S22
**Figure S20.** Comparison of experimental and calculated UV-Vis and ECD spectra of Cbl-4 conformers, calculated at CAM-B3LYP-GD3/6-31G(d)/MDF10/PCM level	S23
**Figure S21.** Comparison of experimental and calculated RRaman and RROA spectra of Cbl-1 conformers, calculated at CAM-B3LYP-GD3/6-31G(d)/MDF10/PCM level	S24
**Figure S22.** Comparison of experimental and calculated FFR-Raman and FFR-ROA spectra of Cbl-1 conformers, calculated at CAM-B3LYP-GD3/6-31G(d)/MDF10/PCM level	S25
**Figure S23.** Comparison of experimental and calculated RRaman and RROA spectra of Cbl-4 conformers, calculated at CAM-B3LYP-GD3/6-31G(d)/MDF10/PCM level	S26
**Figure S24.** Comparison of experimental and calculated FFR-Raman and FFR-ROA spectra of Cbl-4 conformers, calculated at CAM-B3LYP-GD3/6-31G(d)/MDF10/PCM level	S27
**Table S8.** Cartesian coordinates of Cbl-1 conformers optimized at CAM-B3LYP-GD3/6-31G(d)/MDF10/PCM theory level	S28
**Table S9.** Cartesian coordinates of Cbl-4 conformers optimized at CAM-B3LYP-GD3/6-31G(d)/MDF10/PCM theory level	S30
**References**	S32

Experimental Procedures

Materials

Cyanocobalamin (**Cbl-1**, (CN)Cbl, vitamin B_{12}) was purchased from Merck (Sigma-Aldrich). Chemical derivatives of vitamin B_{12}, such as (CN)Cbl(*c*-lactone) (**Cbl-2**), (CN)13-*epi*-Cbl(*e*-lactone) (**Cbl-3**) and (CN)13-*epi*-Cbl(*e*-CO_2Me)(13-OH) (**Cbl-4**) were synthesized following a well described procedures.[1–3] All solutions, were passed through the Millex® (Merck Millipore™) syringe PTFE filters (pore size 0.2 µm) to eliminate solid impurities.

Electronic Absorption (UV-Vis) and Electronic Circular Dichroism (ECD) measurements

UV-Vis and ECD spectra of vitamin B_{12} and its derivatives in water media were recorded in the 230-800 nm spectral range at room temperature in distilled water (0.1 mg/mL). The solutions were measured in the 10 mm quartz optical cells. All spectra were recorded in a single scan using Jasco J−1500 spectropolarimeter with 100 nm min^{-1} scanning speed, step size of 0.1 nm, 1 nm bandwidth, and a response time of 1 s. Spectra were background-corrected using solvents recorded under the same conditions (**Figure 2**, **S1** and **S2**).

Raman and Raman Optical Activity (ROA) measurements

Resonance Raman (RRaman) and ROA (RROA) spectra of cobalamins in aqueous solutions (0.1 mg/mL) were registered on a commercially available SCP-ROA *Chiral*RAMAN-2X™ spectrometer (BioTools Inc.) at 7 cm^{-1} spectral resolution within 250-2500 cm^{-1} employing the excitation wavelength of 532 nm. The RRaman and RROA spectra were collected with a laser power of 200-300 mW and an integration time of 4 s. Far from resonance Raman (FFR Raman) and ROA (FFR ROA) spectra were measured for saturated cobalamin solutions (7-37 mg/mL) on home-built SCP-ROA spectrometer[4,5] at 12.5 cm^{-1} spectral resolution in the range of 200-1900 cm^{-1} using the 785 nm laser source. The laser power of 220 mW and the integration time of 8 s were used to obtain FFR spectra. Other experimental conditions for each sample (i.e. concentration, laser power, and total data collection time) are given in **Table S1**. Moreover, to minimize the ECD-Raman effect on RROA spectra of cobalamin species with a relatively high ECD signal occurring in the range of ROA scattering (i.e. **Cbl-3** and **Cbl-4**), we registered RROA spectra at fairly low concentration (0.1 mg/mL) and we set the L' pathlength to ~0, i.e. by focusing the laser close to the front cell wall. In the case of near-infrared ROA spectra, the ECD signal was sufficiently shifted from the excitation wavelength (785 nm), therefore it allowed us to measure the ROA spectra of cobalamins free from the ECD-Raman interference effect. Minor baseline corrections of both resonance and far-from resonance Raman and ROA spectra were also applied using OriginPro software (**Figure 4** and **S3-17, S21-24**).

Table S1. Set of Raman and ROA measurement parameters of studied compounds.

Compound	λ_{ex}= 532 nm			λ_{ex}= 785 nm	
	c [mg/mL]	laser power [mW]	total data collection time [h]	c [mg/mL]	total data collection time [h]
**Cbl-1**	0.1	200	24	10	44
**Cbl-2**		200	31	7	42
**Cbl-3**		300	94	9	58
**Cbl-4**		300	84	37	41

Calculations

MD simulations

Initial geometries of **Cbl-1** and **Cbl-4** were obtained from the x-ray crystallography structures, deposited in the Cambridge Structural Database (CSD), Refcodes: OBIQEY[6] and FINLAS,[7] respectively. The molecular dynamics (MD) conformational search tool implemented in Gabedit software[8] was used to generate 10 lowest-energy conformers of the studied molecular systems, employing the Universal force field (UFF).[9] A simulated annealing procedure was used, including heating (T=1000 K), equilibration, and production runs that lasted 1, 1, and 10 ps, respectively; the time step was 1 fs. Velocity Verlet algorithm was employed in trajectory calculations. At the end of the molecular dynamics calculations, the geometries of the lowest energy conformers were optimized, and similar structures were discarded.

Quantum chemical calculations

The obtained set of conformers of **Cbl-1** and **Cbl-4** were optimized at four theory levels: B3LYP[10–13], B3LYP-GD3, CAM-B3LYP[14] and CAM-B3LYP-GD3 and 6-31G(d) basis set using Gaussian G16.C01 software.[15] The MDF10 pseudopotential and basis set were used for the Co atom. As **Cbl-x** are rather large molecular systems, composed of rigid corrin ring but also rather loose side chains and pseudonucleotide moiety, we used here the Grimme's Dispersion correction[16] to take into account the long-range intramolecular interactions of those parts of the molecule. The solvent (water) was modeled using the PCM model (IEFPCM).[17] Electronic absorption energies and intensities (oscillator and rotatory strengths) were calculated using TD-DFT, for the first 100 electronic states, at the same level of theory as for the geometry optimizations. Vibrational frequencies and Raman and ROA intensities were calculated employing the same level. The excitation wavelengths used in the polarizability calculations were selected in a way to mimics the experimental conditions. Although the experimental incident laser wavelengths are 532 and 785 nm, the theoretical electronic transition energies are blue-shifted compared to the experiment, and the excitation wavelengths were adapted accordingly, to mimic both (pre-)resonance and far from resonance conditions (FFR). The pre-resonance Raman calculations available in the Gaussian G16 software were employed to mimic the resonance conditions. It is a strong approximation, however, it gives relatively good results (sign and CID, ROA/Raman ratios) for many systems. In this approach, Raman excitation energies need to be selected carefully to not coincide with the theoretical electronic absorption energies. The values of 430 nm and 650 nm were used to mimic the experimental conditions for 532 nm and 785 nm in CAM-B3LYP calculations, while 480 and 700 nm for B3LYP. Smooth theoretical UV-Vis/ECD spectra of the lowest energy conformers were obtained by convolution with Gaussian functions of 0.1 eV full width at half maximum (FWHM). Smoothed theoretical ROA and Raman spectra were convoluted using Lorentzian functions (FWHM, γ=15 cm^{-1}) and a temperature correction factor (T=298 K):

$$S(\tilde{\nu}) = \frac{1}{\tilde{\nu}} \cdot \frac{1}{1 - e^{-\frac{\hbar\tilde{\nu}}{k_B T}}} \cdot \sum_i I_i \cdot \frac{2}{\pi} \cdot \frac{\gamma}{4(\tilde{\nu} - \tilde{\nu}_i)^2 + \gamma^2} \quad (1)$$

Where $\tilde{\nu}$ is a vibrational frequency, k_B is Boltzmann constant, $\tilde{\nu}_i$ and I_i are frequencies and intensities of i-th transition.

Final averaged spectra were obtained using Boltzmann-averaging of the lowest energy conformer spectra using relative Gibbs free (ΔG) and zero-point corrected energies (ΔE_{ZPE}).

Similarity index

To examine what theory level is the most suitable for the ROA and Raman calculations of **Cbl-x** systems, we took into account several theory levels used in the past studies of **Cbl-x** and we did the similarity analysis in a few selected, reliable spectral regions. We used two programs of Andrushchenko, V.: Spectra Similarity (SpecSim), Prague, 2020, and Spectra Scaling (SpecScale), Prague, 2021, that perform Polavarapu's similarity analysis.[18,19] The *Sim*RAM and *Sim*ROA are defined as:

$$Sim\text{RAM} = \frac{I_{co}}{I_{cc} + I_{oo} - I_{co}} \qquad Sim\text{ROA} = \frac{I_{co}}{I_{cc} + I_{oo} - |I_{co}|} \quad (2)$$

here the c and o denotes calculated and experimental spectra, and I_{ij} define overlap of two spectra:

$$I_{ij} = \int F_i(\tilde{\nu}) F_j(\tilde{\nu}) d\tilde{\nu} \quad (3)$$

*Sim*RAM ranges from 0 (zero overlap) to 1 ($F_i(\tilde{\nu}) = F_j(\tilde{\nu})$), and *Sim*ROA from -1 ($F_i(\tilde{\nu}) = -F_j(\tilde{\nu})$), to 1 ($F_i(\tilde{\nu}) = F_j(\tilde{\nu})$).[18,19]

Calculated and experimental Raman spectra used in the similarity analysis as well as used in **Figures 4** and **S15** were normalized in the range of 0 to 1, then ROA spectra were normalized accordingly to Raman to keep the original CID values. We determined the best scaling factors for Boltzmann averaged (ΔG) spectra (both vibrational frequencies and intensities, f_X and f_Y scaling factors) to get the best similarity overlap of experimental and calculated spectra in four spectra ranges: 1650-550, 1650-1420, 1420-1000 and 1000-550 cm^{-1}. We used f_X factors obtained from the similarity analysis of the Raman spectra in the range of 1650-550 cm^{-1} as scaling factors for spectra presented in **Figures 4**, **S3-S6**, **S15-S17**, and **S21-24** as well as for scaled calculated frequencies in the **Table S2** and **S3**.

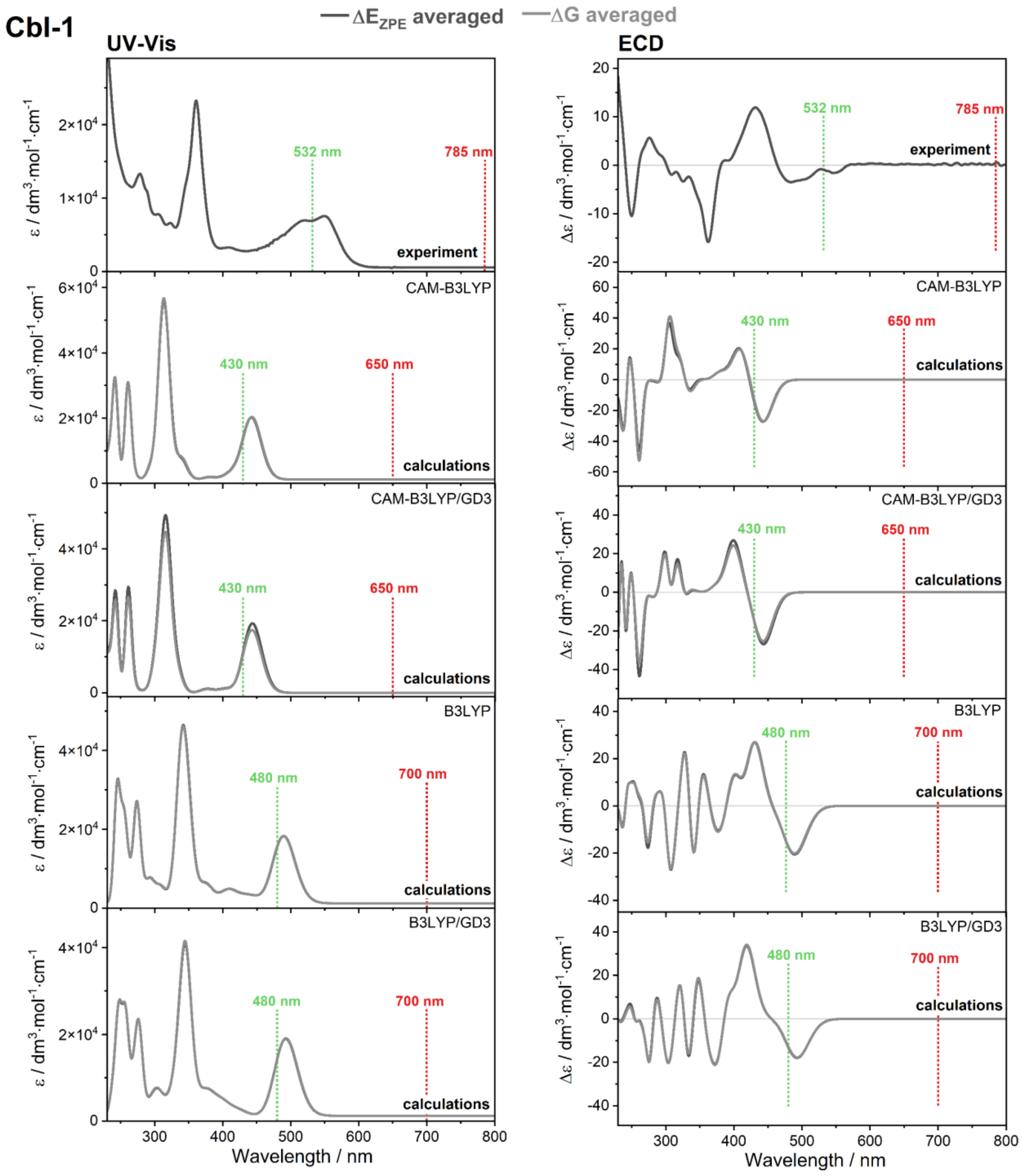

Figure S1. Comparison of experimental and calculated (Boltzmann average) UV-Vis (left panel) and ECD (right panel) spectra of **Cbl-1**, calculated at different theory levels. The 532 and 785 nm excitation wavelengths and the four used in the calculations (430/480 and 650/700 nm) are indicated by the green and red dotted lines, respectively.

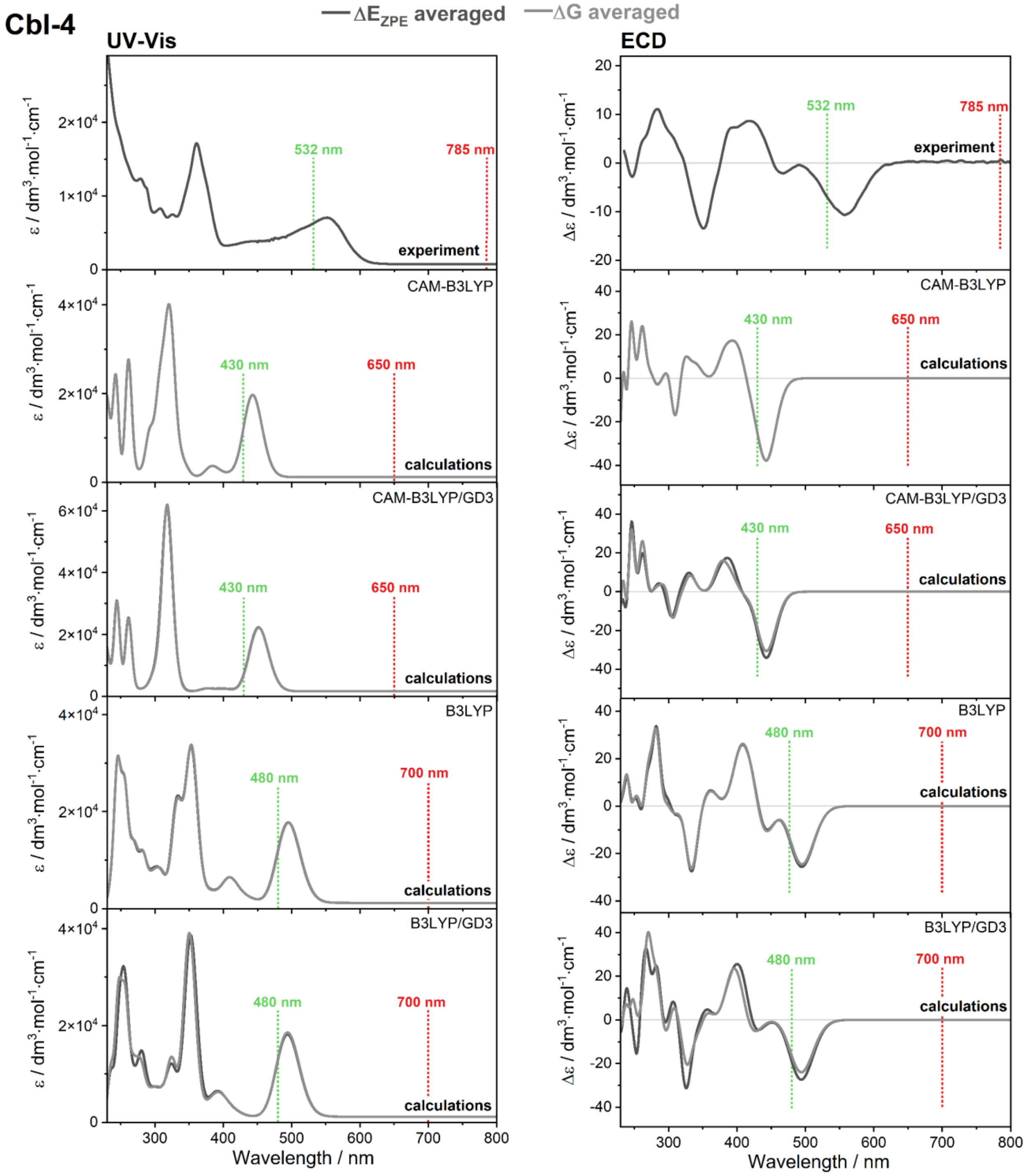

Figure S2. Comparison of experimental and calculated (Boltzmann average) UV-Vis (left panel) and ECD (right panel) spectra of **Cbl-4**, calculated at different theory levels. The 532 and 785 nm excitation wavelengths and the four used in the calculations (430/480 and 650/700 nm) are indicated by the green and red dotted lines, respectively.

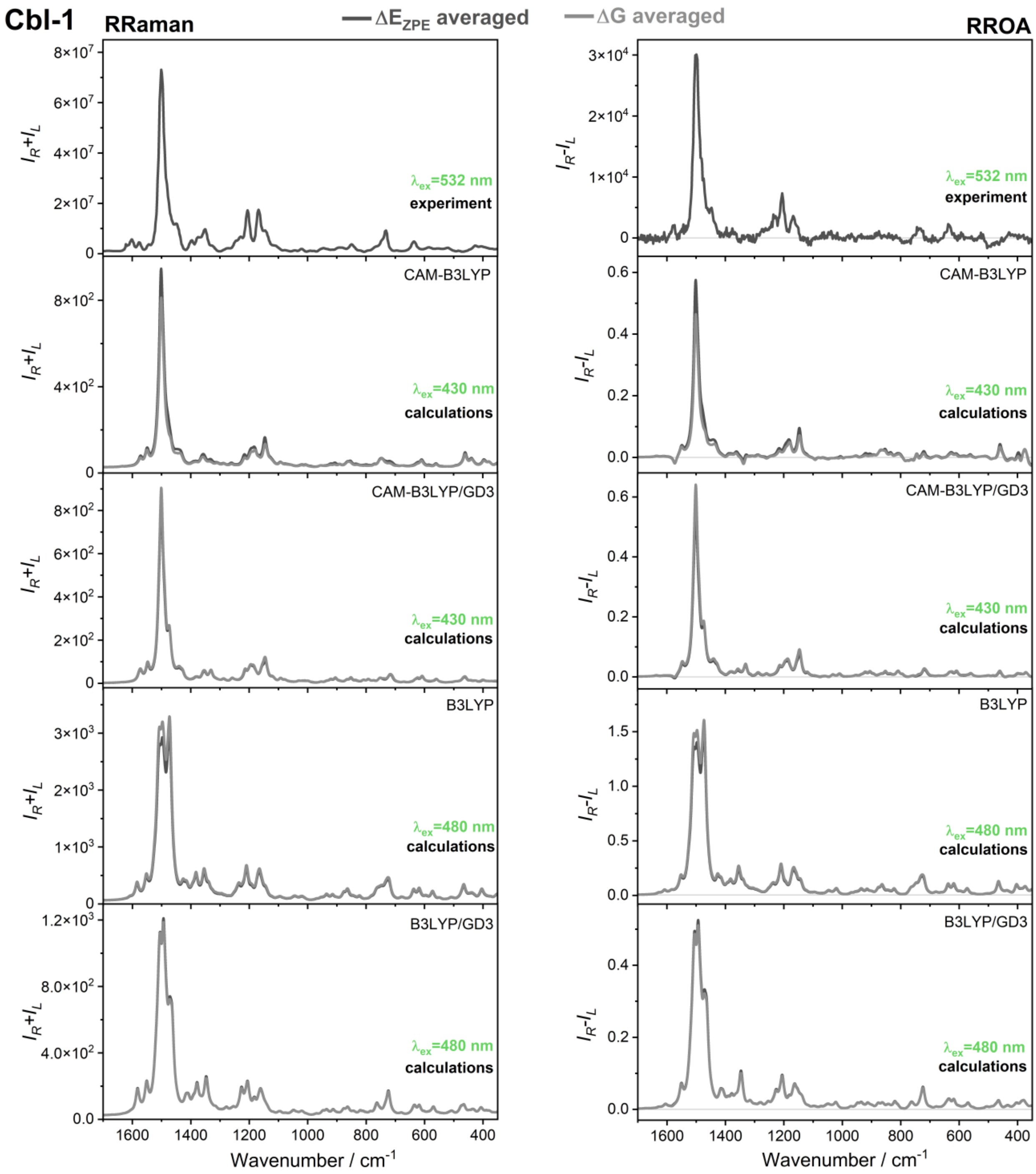

Figure S3. Comparison of experimental and calculated (Boltzmann average) RRaman (left panel) and RROA (right panel) spectra of **Cbl-1**, calculated at different theory levels. The experimental spectra were obtained with an excitation wavelength of 532 nm, while the calculated spectra with excitation wavelengths of 430 or 480 nm. The calculated vibrational frequencies were scaled by a factor of 0.946, 0.941, 0.979, and 0.971 for spectra obtained with CAM-B3LYP, CAM-B3LYP-GD3, B3LYP, and B3LYP-GD3 theory level, respectively.

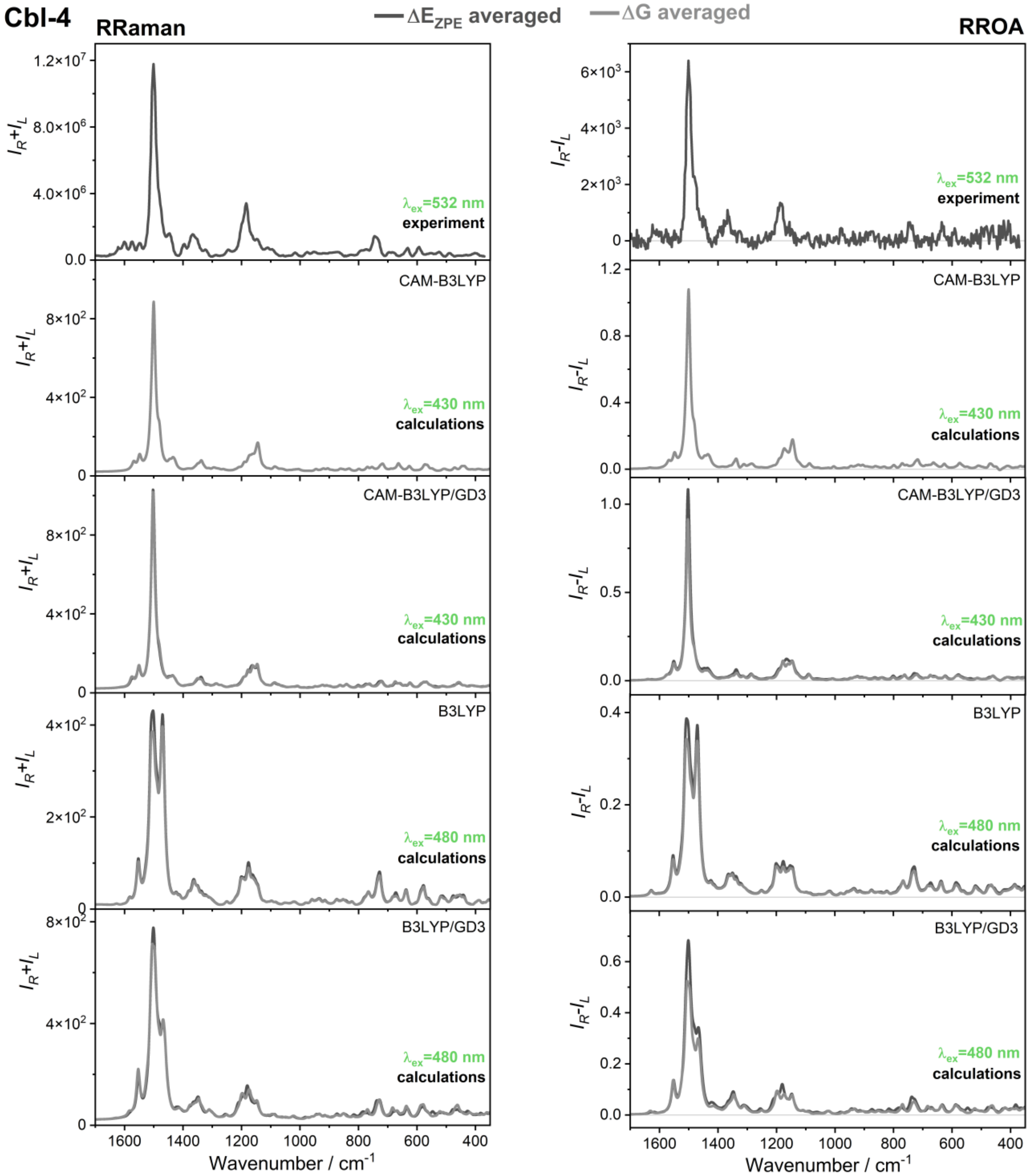

Figure S4. Comparison of experimental and calculated (Boltzmann average) RRaman (left panel) and RROA (right panel) spectra of **Cbl-4**, calculated at different theory levels. The experimental spectra were obtained with an excitation wavelength of 532 nm, while the calculated spectra with excitation wavelengths of 430 or 480 nm. The calculated vibrational frequencies were scaled by a factor of 0.943, 0.940, 0.977, and 0.970 for spectra obtained with CAM-B3LYP, CAM-B3LYP-GD3, B3LYP, and B3LYP-GD3 theory level, respectively.

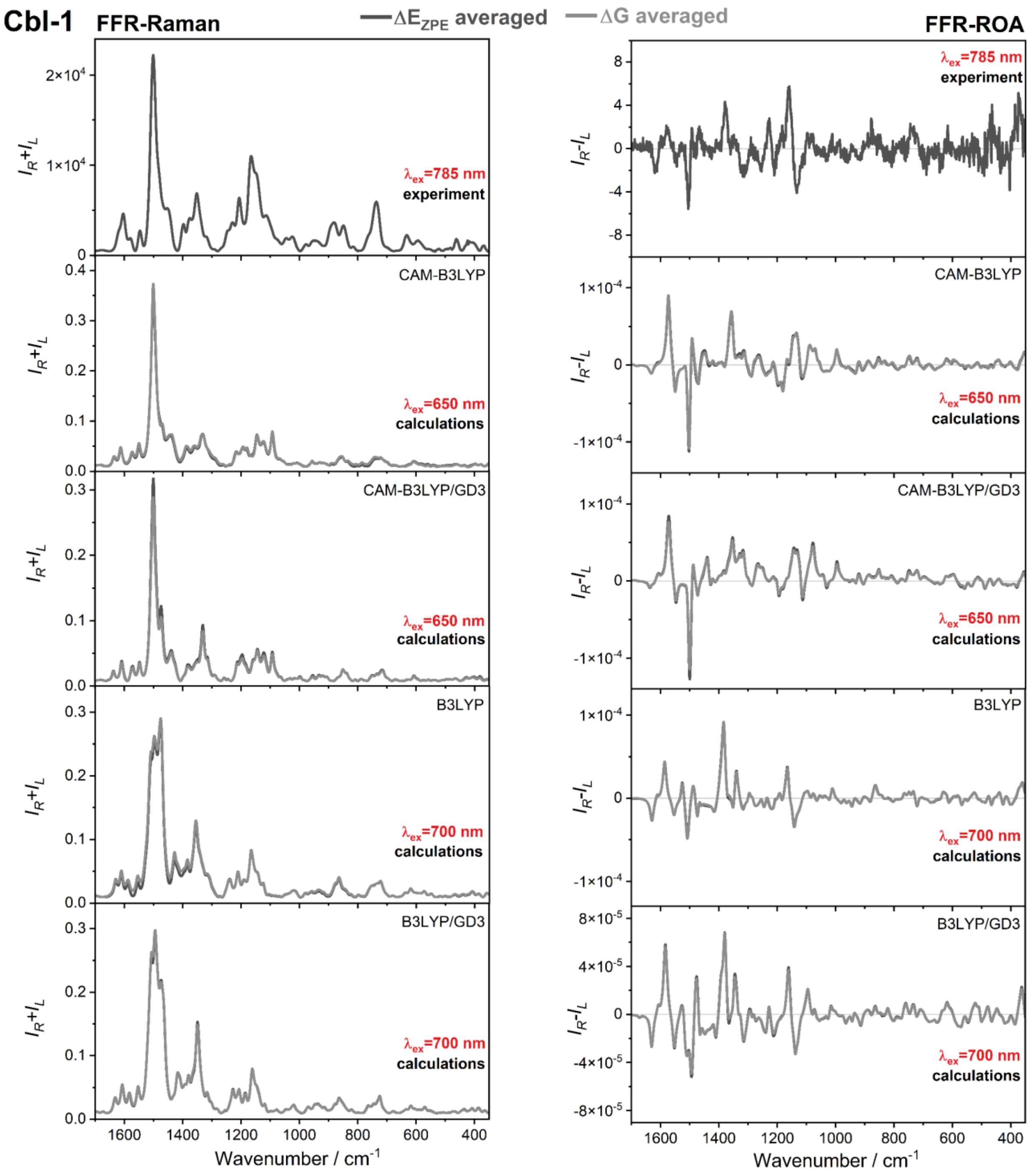

Figure S5. Comparison of experimental and calculated (Boltzmann average) FFR-Raman (left panel) and FFR-ROA (right panel) spectra of **Cbl-1**, calculated at different theory levels. The experimental spectra were obtained with an excitation wavelength of 785 nm, while the calculated spectra with excitation wavelengths of 650 or 700 nm. The calculated vibrational frequencies were scaled by a factor of 0.946, 0.941, 0.980, and 0.972 for spectra obtained with CAM-B3LYP, CAM-B3LYP-GD3, B3LYP, and B3LYP-GD3 theory level, respectively.

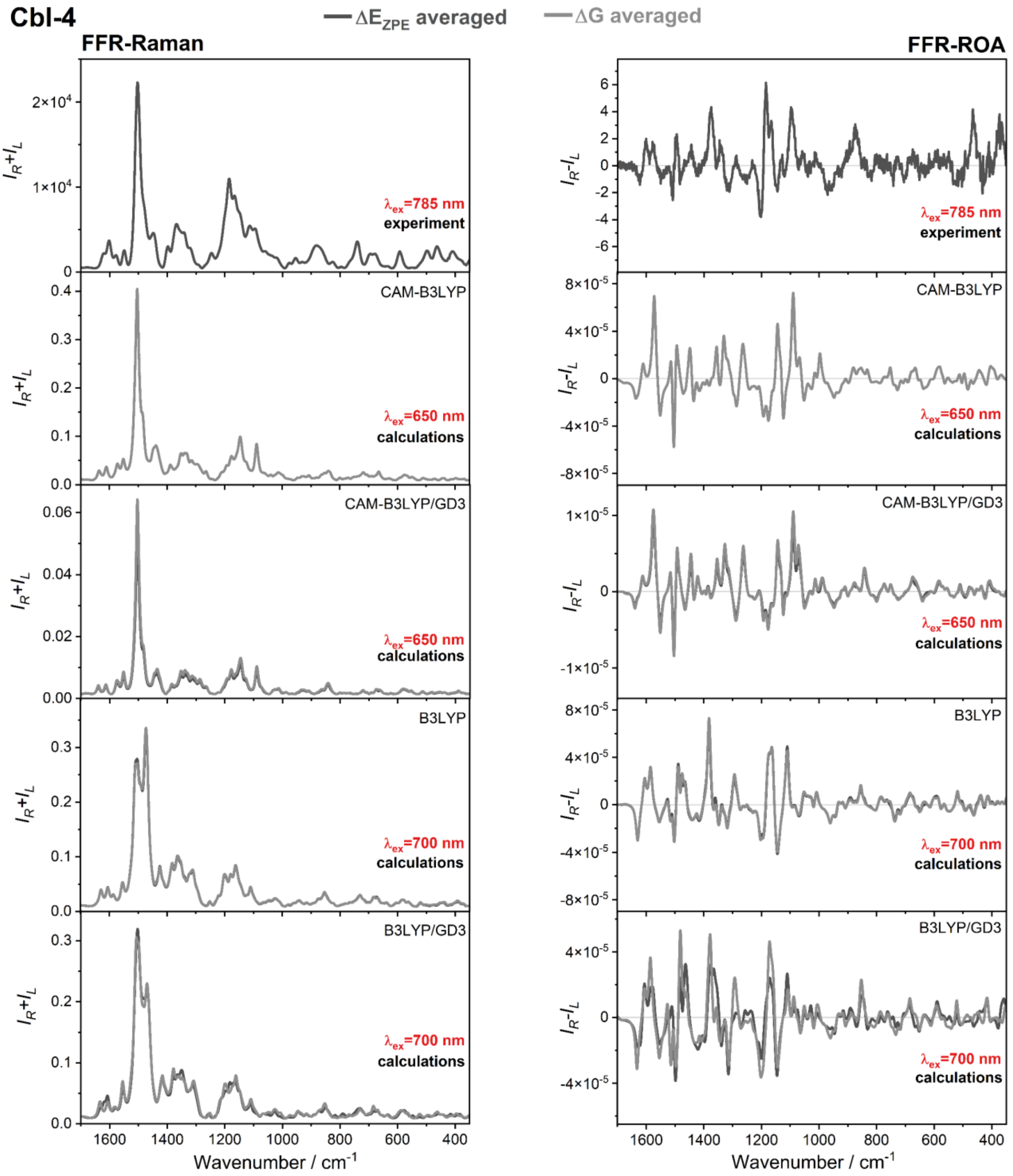

Figure S6. Comparison of experimental and calculated (Boltzmann average) FFR-Raman (left panel) and FFR-ROA (right panel) spectra of **Cbl-4**, calculated at different theory levels. The experimental spectra were obtained with an excitation wavelength of 785 nm, while the calculated spectra with excitation wavelengths of 650 or 700 nm. The calculated vibrational frequencies were scaled by a factor of 0.945, 0.941, 0.978, and 0.971 for spectra obtained with CAM-B3LYP, CAM-B3LYP-GD3, B3LYP, and B3LYP-GD3 theory level, respectively.

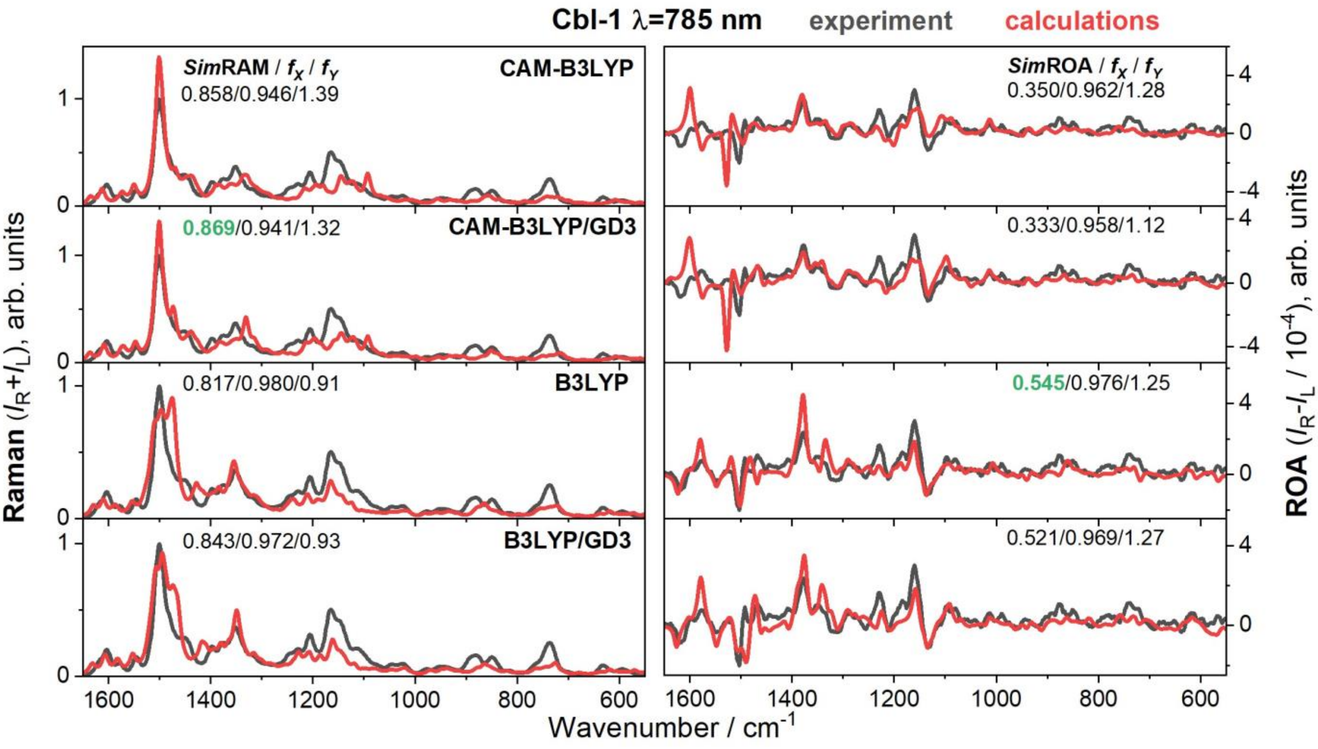

Figure S7. Similarity analysis (*Sim*RAM, *Sim*ROA) for experimental FFR Raman/ROA spectra of Cbl-1 (λ_{ex}=785 nm) in the 1650-550 cm^{-1} range and calculated (Boltzmann average, ΔG) spectra at CAM-B3LYP, CAM-B3LYP-GD3, B3LYP, and B3LYP-GD3. Similarities and scaling factors are provided as *Sim*RAM / f_X / f_Y, or *Sim*ROA / f_X / f_Y. The highest similarity indexes for Raman and ROA are highlighted in green.

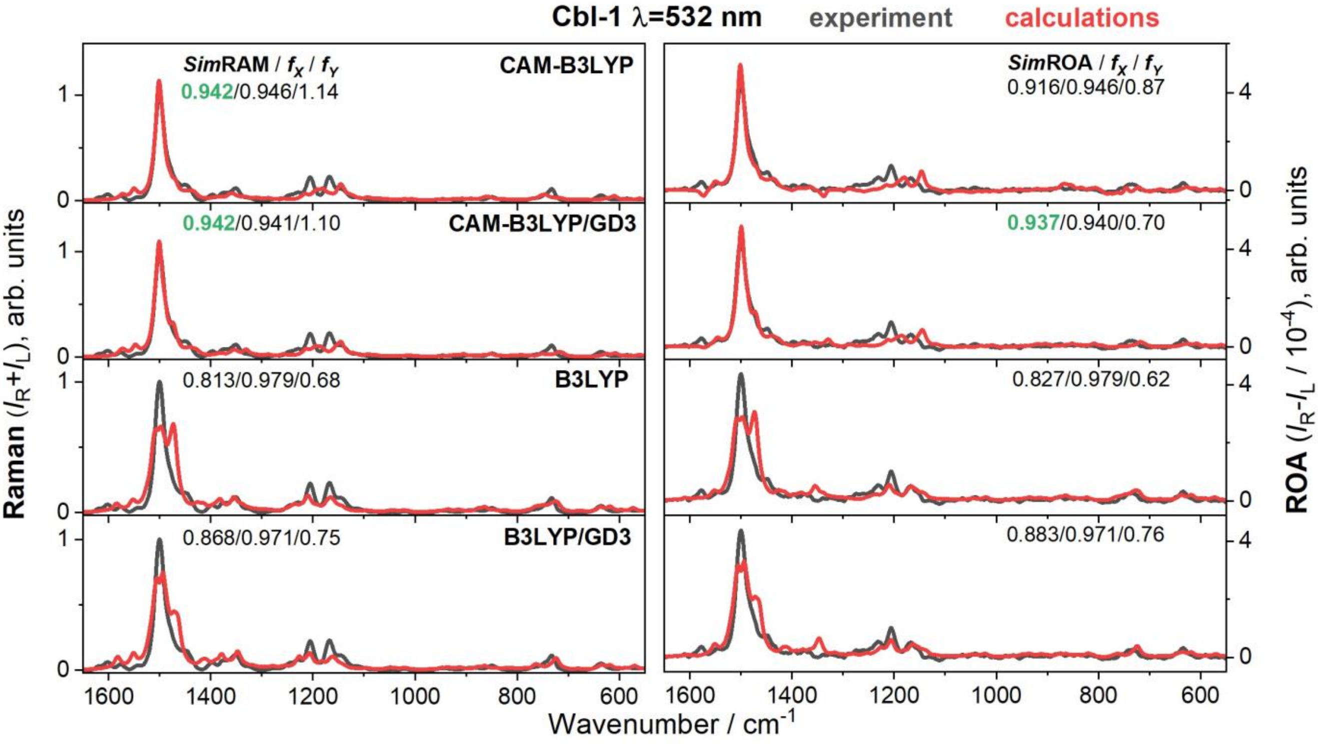

Figure S8. Similarity analysis (*Sim*RAM, *Sim*ROA) for experimental RRaman/RROA spectra of Cbl-1 (λ_{ex}=532 nm) in the 1650-550 cm^{-1} range and calculated (Boltzmann average, ΔG), pre-resonance spectra at CAM-B3LYP, CAM-B3LYP-GD3, B3LYP, and B3LYP-GD3. Similarities and scaling factors are provided as *Sim*RAM / f_X / f_Y, or *Sim*ROA / f_X / f_Y. The highest similarity indexes for Raman and ROA are highlighted in green.

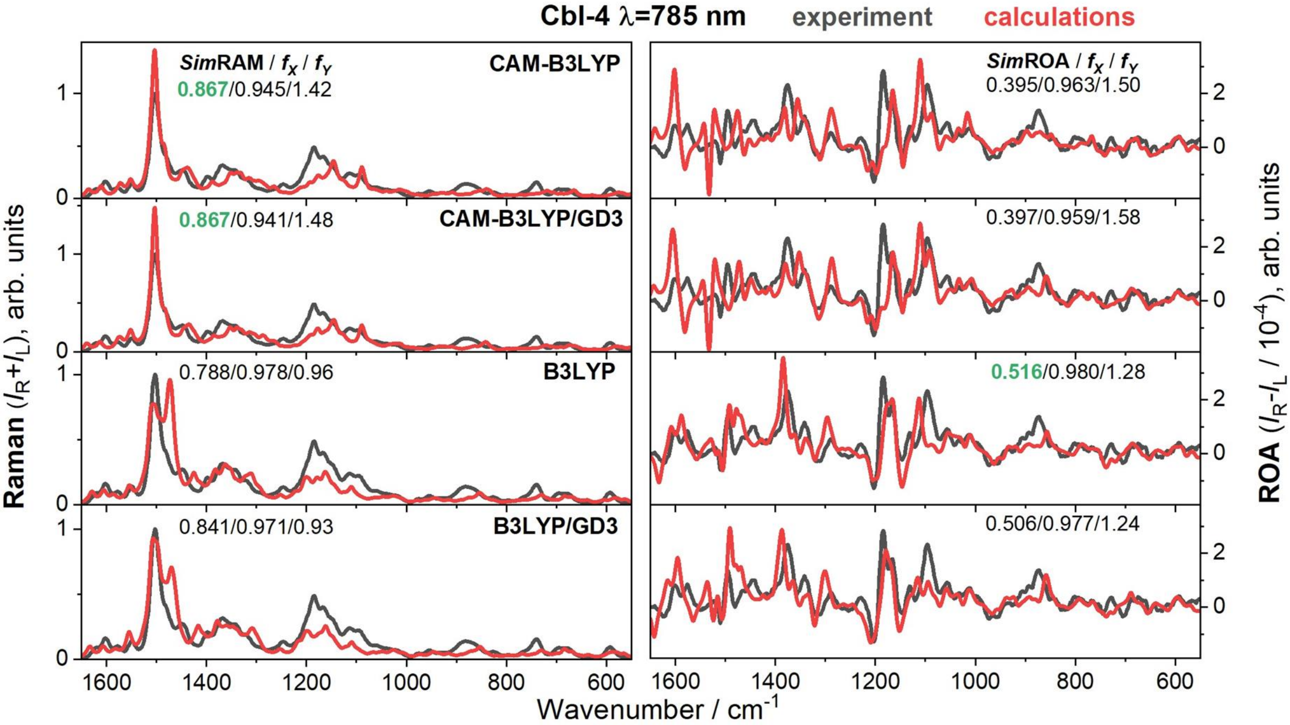

Figure S9. Similarity analysis (*Sim*RAM, *Sim*ROA) for experimental FFR Raman/ROA spectra of Cbl-4 (λ_{ex}=785 nm) in the 1650-550 cm^{-1} range and calculated (Boltzmann average, ΔG) spectra at CAM-B3LYP, CAM-B3LYP-GD3, B3LYP, and B3LYP-GD3. Similarities and scaling factors are provided as *Sim*RAM / f_X / f_Y, or *Sim*ROA / f_X / f_Y. The highest similarity indexes for Raman and ROA are highlighted in green.

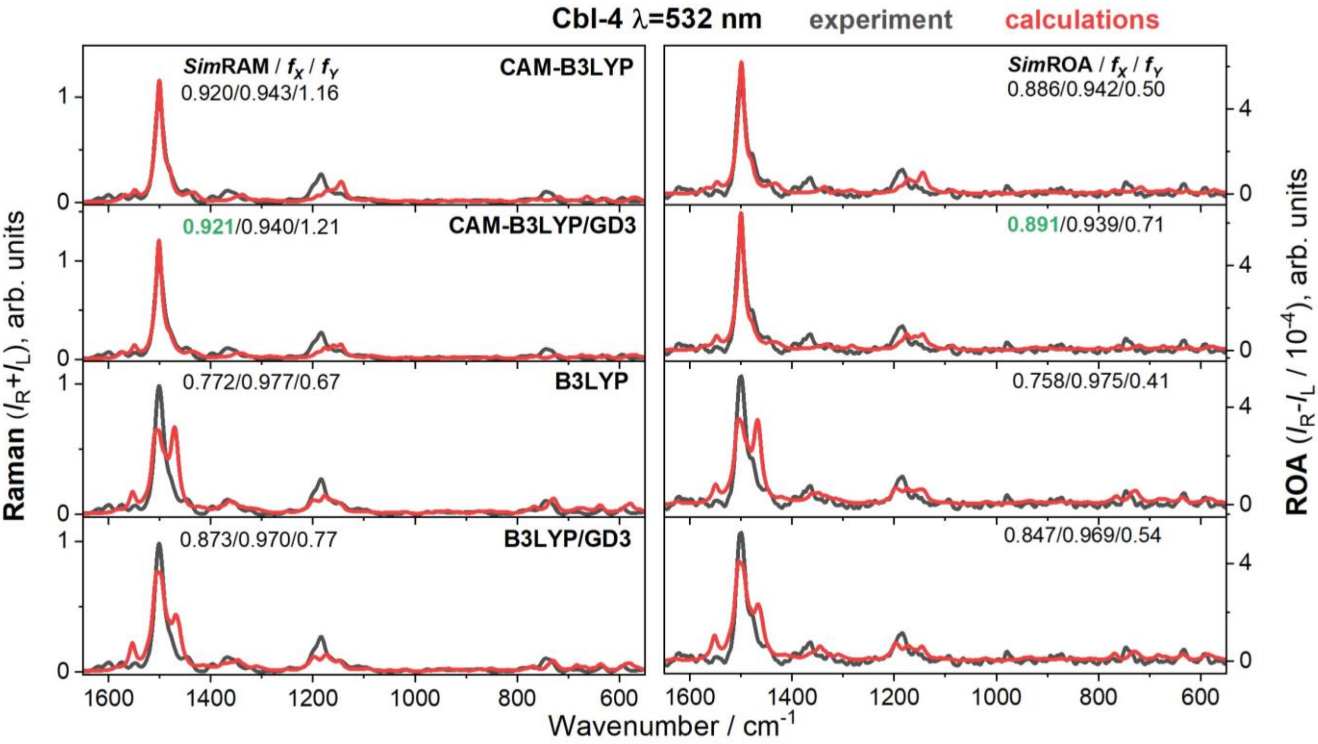

Figure S10. Similarity analysis (*Sim*RAM, *Sim*ROA) for experimental RRaman/RROA spectra of Cbl-4 (λ_{ex}=532 nm) in the 1650-550 cm^{-1} range and calculated (Boltzmann average, ΔG), pre-resonance spectra at CAM-B3LYP, CAM-B3LYP-GD3, B3LYP, and B3LYP-GD3. Similarities and scaling factors are provided as *Sim*RAM / f_X / f_Y, or *Sim*ROA / f_X / f_Y. The highest similarity indexes for Raman and ROA are highlighted in green.

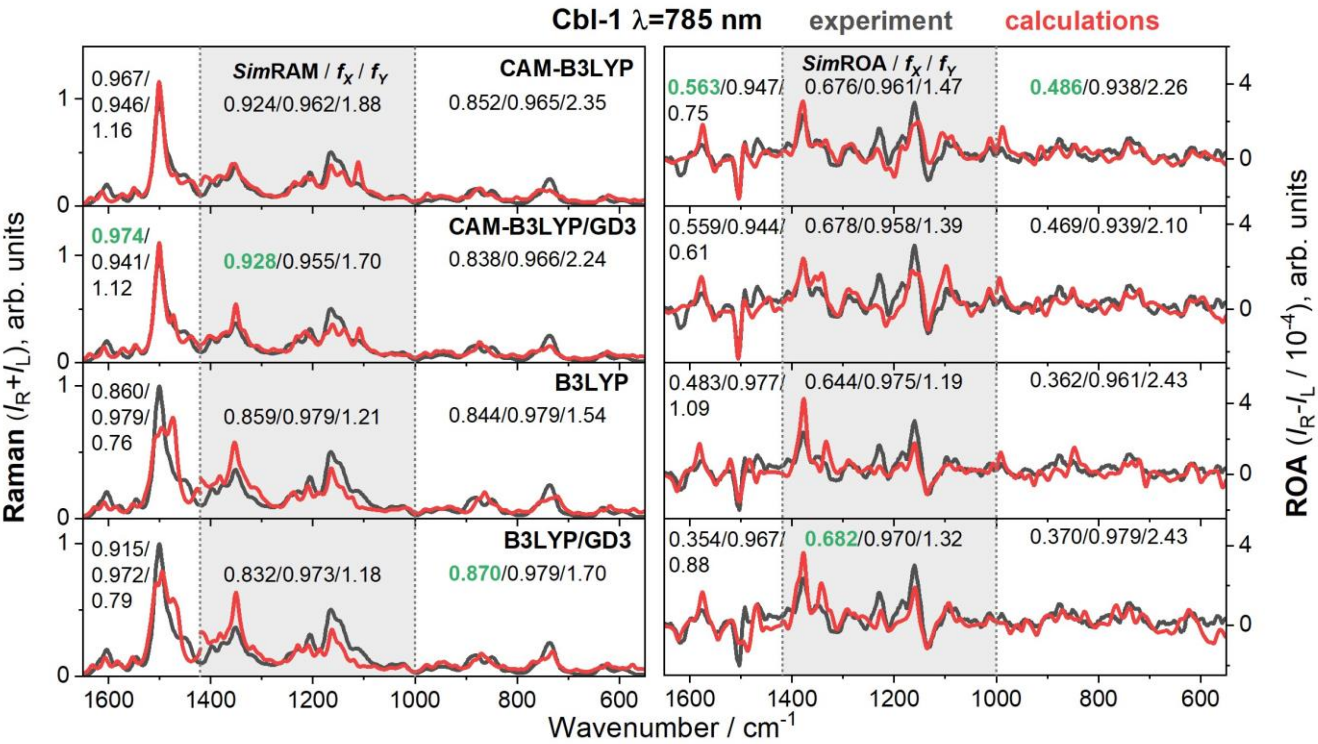

Figure S11. Similarity analysis (*Sim*RAM, *Sim*ROA) for experimental FFR Raman/ROA spectra of Cbl-1 (λ_{ex}=785 nm) in the ranges of 1650-1420, 1420-1000, and 1000-550 cm^{-1} and calculated (Boltzmann average, ΔG) spectra at CAM-B3LYP, CAM-B3LYP-GD3, B3LYP, and B3LYP-GD3. Similarities and scaling factors are provided for each spectral range as *Sim*RAM / f_X / f_Y, or *Sim*ROA / f_X / f_Y. The highest similarity indexes for Raman and ROA at each spectral range are highlighted in green.

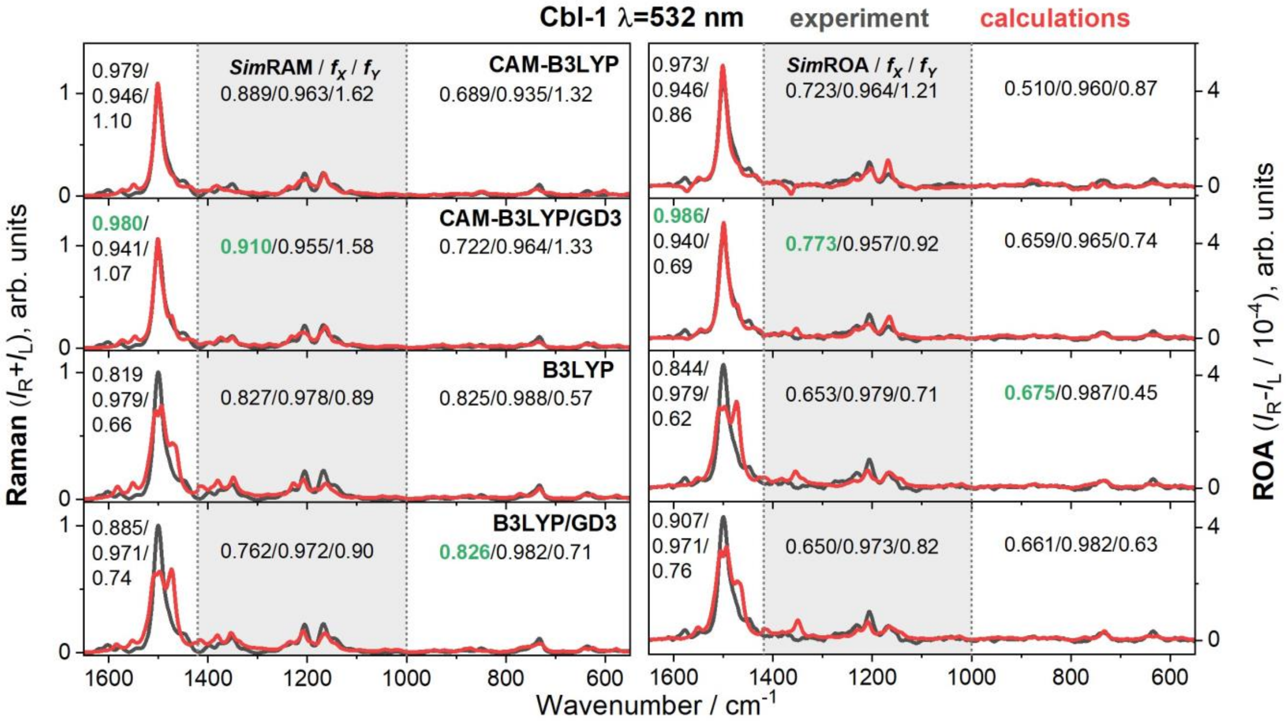

Figure S12. Similarity analysis (*Sim*RAM, *Sim*ROA) for experimental RRaman/RROA spectra of Cbl-1 (λ_{ex}=532 nm) in the ranges of 1650-1420, 1420-1000, and 1000-550 cm^{-1} and calculated (Boltzmann average, ΔG) pre-resonance spectra at CAM-B3LYP, CAM-B3LYP-GD3, B3LYP, and B3LYP-GD3. Similarities and scaling factors are provided for each spectral range as *Sim*RAM / f_X / f_Y, or *Sim*ROA / f_X / f_Y. The highest similarity indexes for Raman and ROA at each spectral range are highlighted in green.

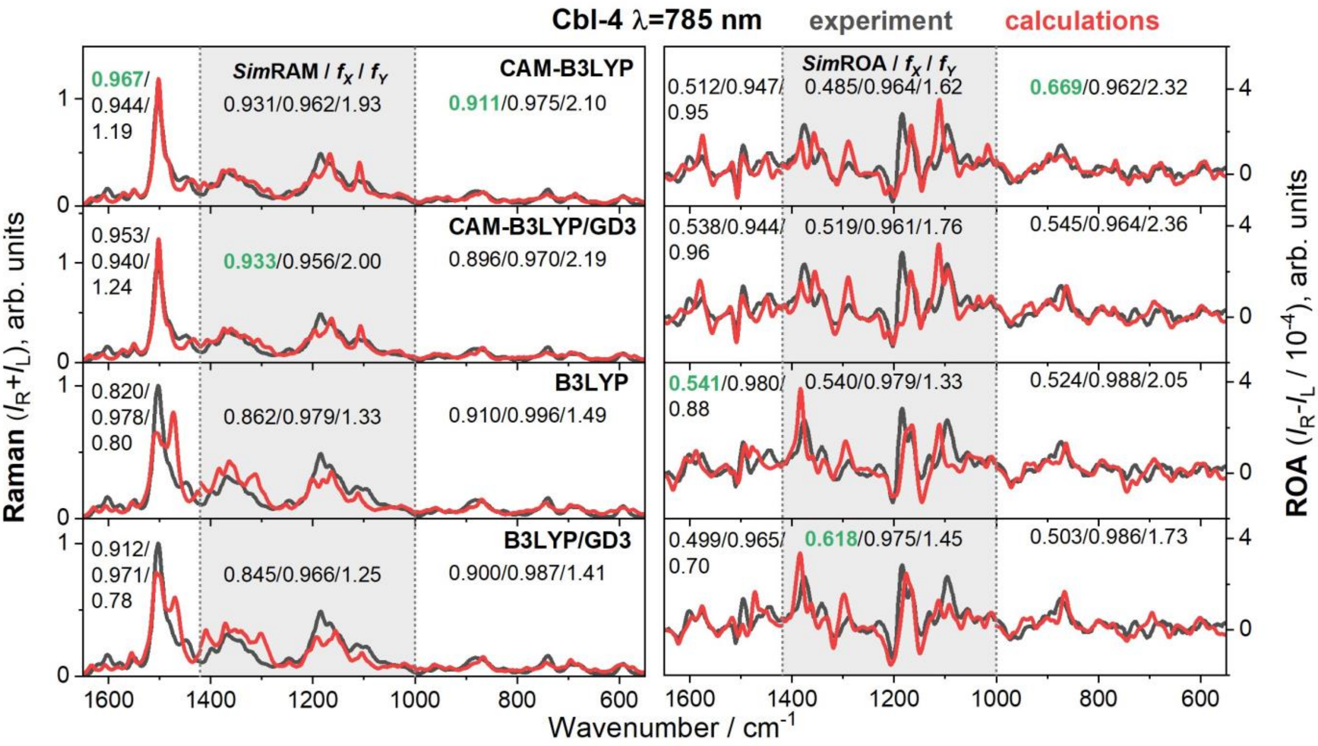

Figure S13. Similarity analysis (*Sim*RAM, *Sim*ROA) for experimental FFR Raman/ROA spectra of Cbl-4 (λ_{ex}=785 nm) in the ranges of 1650-1420, 1420-1000, and 1000-550 cm^{-1} and calculated (Boltzmann average, ΔG) spectra at CAM-B3LYP, CAM-B3LYP-GD3, B3LYP, and B3LYP-GD3. Similarities and scaling factors are provided for each spectral range as *Sim*RAM / f_X / f_Y, or *Sim*ROA / f_X / f_Y. The highest similarity indexes for Raman and ROA at each spectral range are highlighted in green.

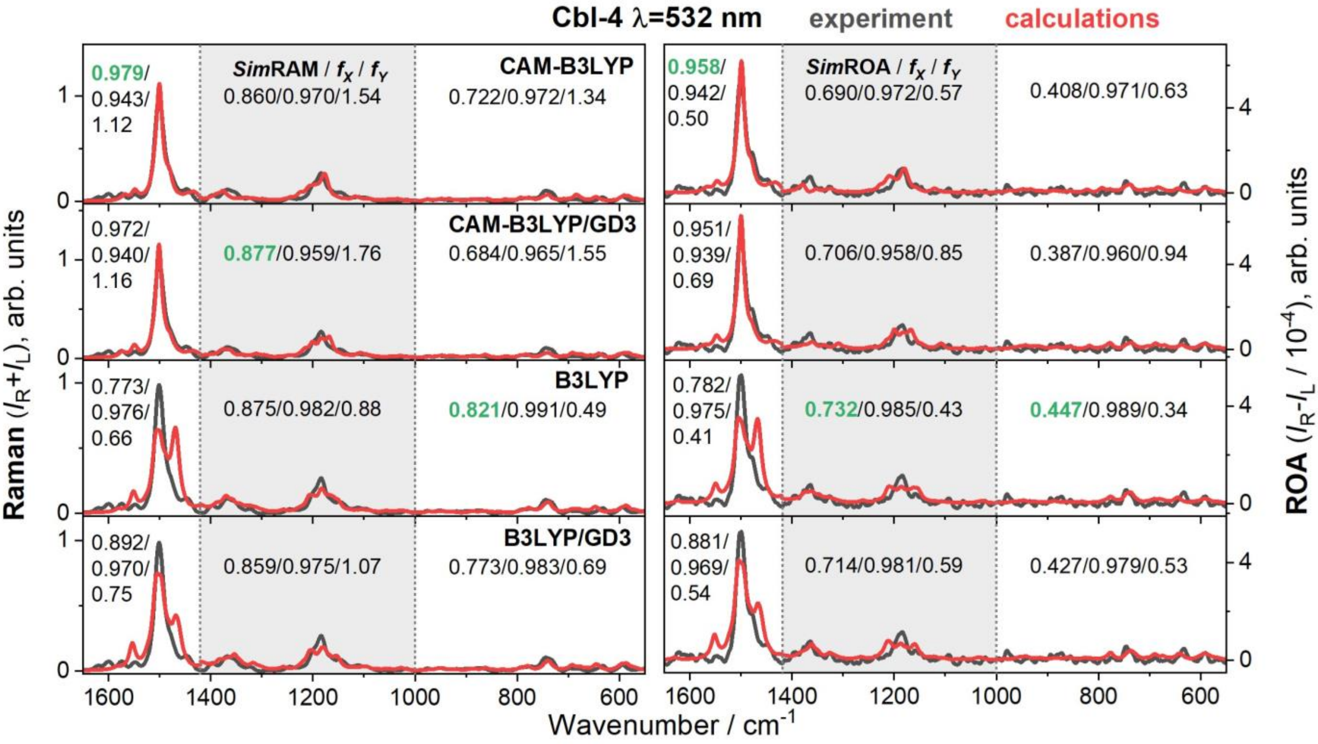

Figure S14. Similarity analysis (*Sim*RAM, *Sim*ROA) for experimental RRaman/RROA spectra of Cbl-4 (λ_{ex}=532 nm) in the ranges of 1650-1420, 1420-1000, and 1000-550 cm^{-1} and calculated (Boltzmann average, ΔG) pre-resonance spectra at CAM-B3LYP, CAM-B3LYP-GD3, B3LYP, and B3LYP-GD3. Similarities and scaling factors are provided for each spectral range as *Sim*RAM / f_X / f_Y, or *Sim*ROA / f_X / f_Y. The highest similarity indexes for Raman and ROA at each spectral range are highlighted in green.

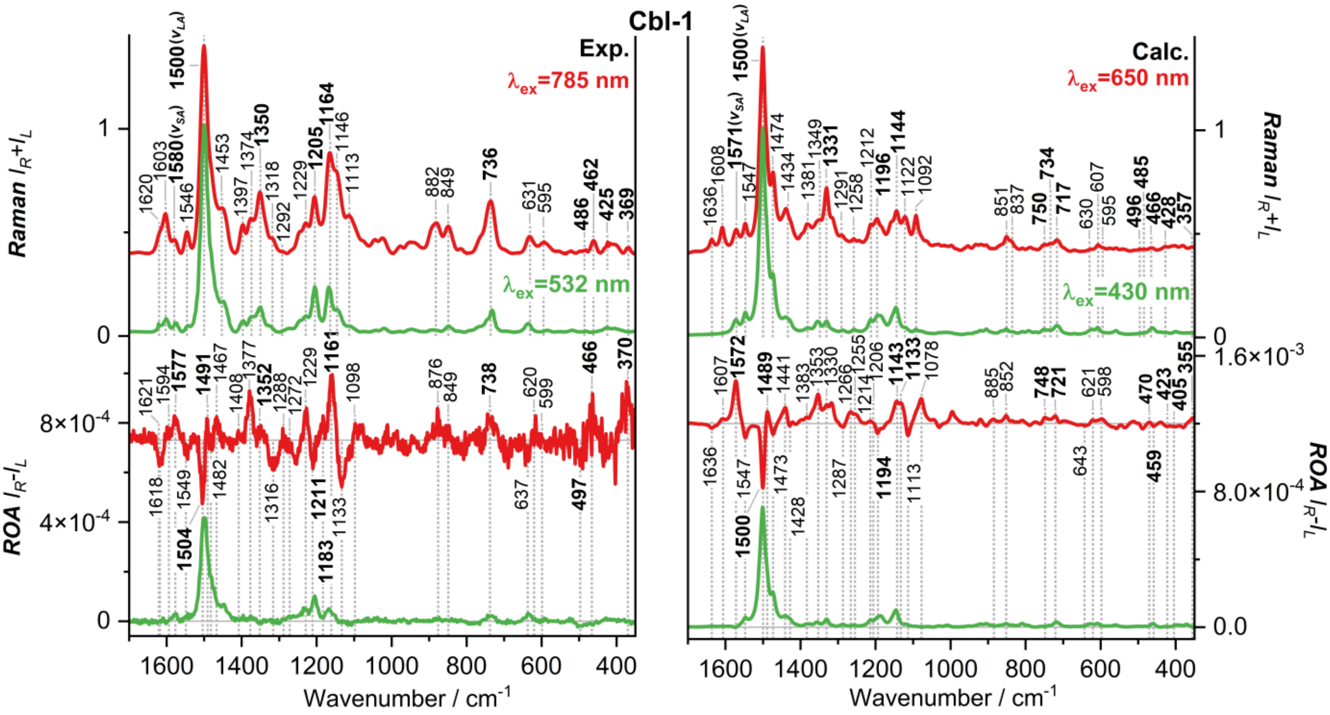

Figure S15. Comparison of the experimental (left panel) and calculated (right panel) Raman and ROA spectra of Cbl-1. CAM-B3LYP-GD3/6-31G(d)/MDF10/PCM theory level and Boltzmann-averaging (ΔG) of the lowest energy conformer spectra were used for the calculated spectra. The experimental spectra are obtained with excitation wavelengths of 532 and 785 nm, while the calculated spectra employ excitation wavelengths of 430 and 650 nm. The calculated vibrational frequencies were scaled by a factor of 0.9410. Spectra normalized preserving CID ratios. The most important bands discussed in the manuscript were highlighted.

Table S2. Calculated (CAM-B3LYP-GD3/6-31G(d)/MDF10/PCM) and experimental frequencies of **Cbl-1**.

	**Calculations**				**Experimental**				
	**650 nm**		**430 nm**		**785 nm**		**532 nm**		**Assignment**
**No**	**Raman** *x0.9410*	**ROA** *x0.9410*	**Raman** *x0.9410*	**ROA** *x0.9410*	**Raman**	**ROA**	**Raman**	**ROA**	
1	1636	1636(-)	1636	1636(+)	1620s	1618(-)	1620s	-	C=C str (5-6), C=N str (4-21)
2	1608	1607(+)	1608	1607(+)	1603	1594(+)	1602	-	C=C str (14-15), C=N str (16-24)
3	1571	1572(+)	1572	1572(-)	1580	1577(+)	1576	1579(+)	C=C str (5-6;10-11), C-H bend (10), C=N str (16-24;4-21), NH_2 bend (scissoring), DmB C=C str
4	1547	1547(-)	1547	1547(+)	1546	1549(-)	1543	1542(+)	(ν_{SA}) C=C str (5-6;10-11;14-15), C=N str (9-22;11-23;16-24;4-21)
5	1500	1500(-)<br>1489(+)	1500	1501(+)	1500	1504(-)<br>1491(+)	1501	1501(+)	(ν_{LA}) C=C str (5-6;9-10;14-15), C=N str (4-21;11-23;16-24, B2-B1;B2-B3), C-H bend, CH_3 bend asym<br>CH_3 bend asym, C-H bend (10), CH_2 bend (scissoring), C=C str (14-15), C=N str (16-24;9-22)
6	1474	1473(-)	1474	1474(+)	1480s	1482(-)	1480s	1480s(+)	CH_3 bend asym, CH_2 bend (scissoring), C=C str (14-15)
7	1434	1441(+)<br>1428(-)	1440	1441(+)	1453	1467(+)<br>1450(-)	1451	1449(+)	CH_3 bend asym, CH_2 bend (scissoring)
8	1381	1383(+)	1380	1380(+)	1397	1408(+)	1397	1397(+)	CH_3 bend sym, CH bend
9	1349	1353(+)	1354	1356(+)	1374	1377(+)	1373	1376(+)	CH_3 bend sym, CH bend, CH_2 wagg
10	1331	1330(+)	1330	1330(+)	1350	1352(+)	1351	1355(-)	CH_2 twist, CH bend, DmB ring breathing
11	1291	1287(-)	1287	1287(+)	1318s	1316(-)	1318s	-	CH bend, CH_2 twist, CH_2 wagg
12	1258	1266(+)<br>1255(+)	1257	1259(+)	1292s	1288(+)<br>1272(+)	1273	-	CH_2 twist, CH_2 wagg, CH bend
13	1212	1214(+)<br>1206(+)	1213	1213(+)	1229	1229(+)	1228	1227(+)	CH_2 wagg, CH bend, NH bend, CH_3 rock
14	1196	1194(-)	1190	1187(+)	1205	1211(-)	1205	1204(+)	CH_2 wagg, CH bend, CH_3 rock, C-N str, C-C str, CH_2 twist
15	1144	1143(+)<br>1133(+)	1146	1146(+)	1164	1183(+)<br>1161(+)	1168	1168(+)	CH_2 twist, CH bend, CH_3 rock, C-N str (6-22;14-23), C-C str (corr. ring)
16	1122	1113(-)	1121s	1120(+)	1146s	1133(-)	1146s	-	CH_2 twist, CH bend, CH_3 rock, C-C str,
17	1092	1078(+)	1092	1075(-)	1113	1098(+)	1115s	1115(-)	C-N str (6-22;14-23,1-21,65-59), C-C str, CH_2 twist, CH bend, CH_3 rock, NH_2 rock
18	851	885(+)	852	852(+)	882	876(+)	892	881(+)	CH_3 rock, C-C str, Co-N str
19	837s	852(+)	837s	837(s)	849	849(+)	849	-	CH_3 rock, C-C str, NH_2 rock
20	750<br>734<br>717	748(+)<br>721(+)	752<br>739<br>717	757(+)<br>719(+)	736	738(+)	732	730(+)	CH_3 rock,CH_2 rock, corrin ring tors and bend, C-C str, NH_2 twist, DmB ring str
21	630	643(-)<br>621(+)	624	627(+)	631	637(-)<br>620(+)	635	639(+)	NH_2 twist, corrin ring tors and bend
22	607<br>595s	598(+)	609	609(+)	595	599(+)	586	585(+)	NH_2 twist, corrin ring tors and bend
23	496<br>485<br>466	488(-)<br>470(+)<br>459(-)<br>441(+)	462	460(+)<br>440(-)	486<br>462	497(-)<br>466(+)	485	497(-)	Co-C≡N bend, C≡N twist, DmB ring str, Co-N bend
24	428<br>409<br>402	423(+)<br>405 (+)	428	426(+)	425	-	425	429(+)	Co-C≡N bend, corrin ring tors and bend, CCN, CNC bend, CC, CN tors, NH_2 twist, NH_2 wagg
25	357	355(+)	359	372(+)	369	370(+)	333	-	Co-C≡N bend, corrin ring tors and bend, CCN, CNC bend, CC, CN tors, NH_2 twist, NH_2 wagg, CH_3 tors

Abbreviations: str – stretching; bend – bending; sym – symmetric; asym – asymmetric; wagg – wagging; twist – twisting; rock– rocking; tors – torsion; s – shoulder, (+) and (–) – positive and negative ROA intensities.

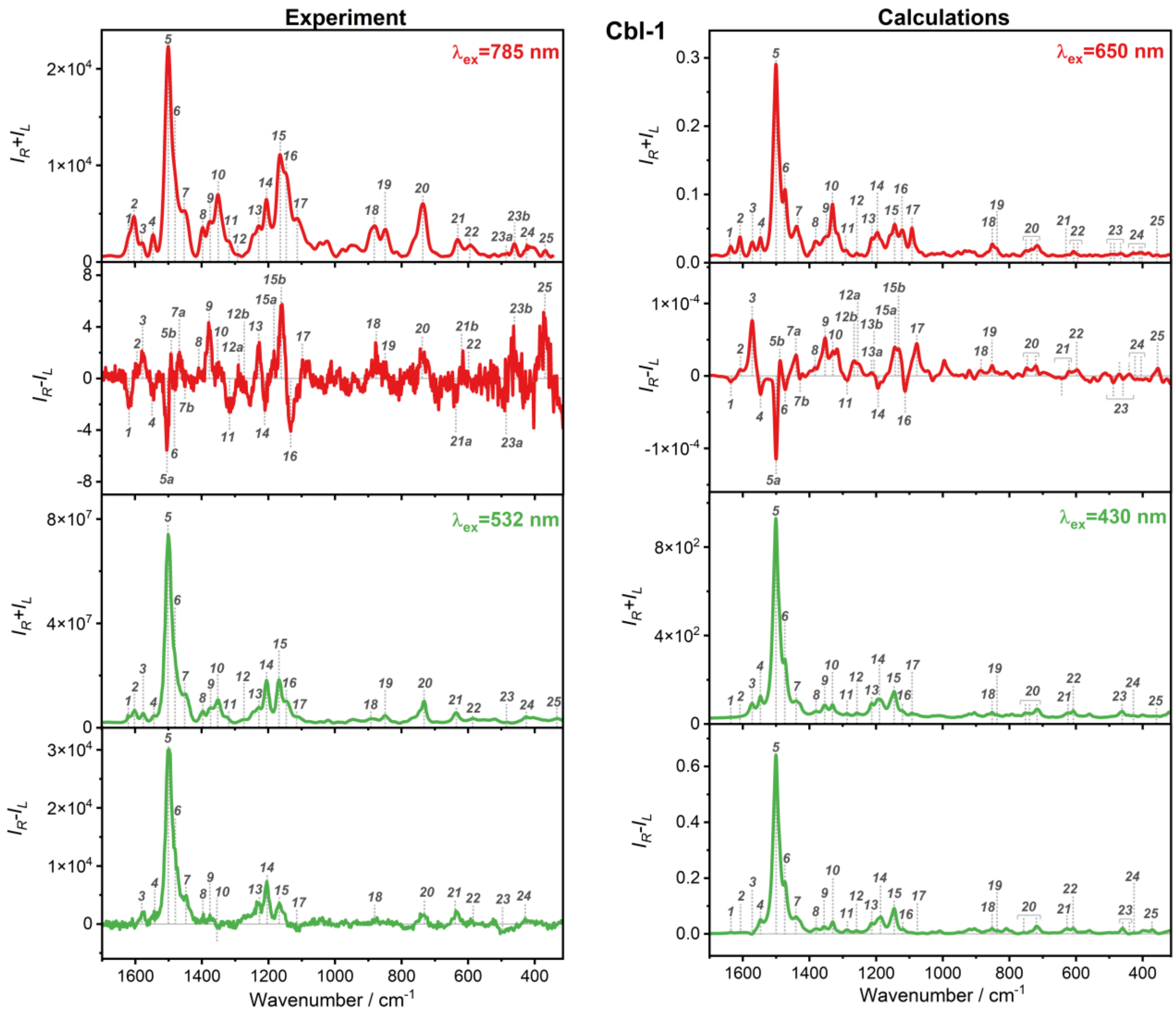

Figure S16. Experimental FFR-ROA/FFR-Raman and RROA/RRaman spectra (left panel) of **Cbl-1** in comparison with calculated (Boltzmann average, ΔG) spectra (right panel) using CAM-B3LYP-GD3/6-31G(d)/MDF10/PCM level. The experimental spectra were obtained with excitation wavelengths of 785 and 532 nm, while the calculated spectra with excitation wavelengths of 650 and 430 nm, respectively. The calculated vibrational frequencies were scaled by a factor of 0.9410. The numbering of vibrational bands corresponds to the band assignments in **Table S2**.

Table S3. Calculated (CAM-B3LYP-GD3/6-31G(d)/MDF10/PCM) and experimental frequencies of **Cbl-4**.

	**Calculations**				**Experimental**				
	**650 nm**		**430 nm**		**785 nm**		**532 nm**		**Assignment**
**No**	**RS**<br>*x0.9410*	***ROA***<br>*x0.9410*	**RS**<br>*x0.9400*	***ROA***<br>*x0.9400*	***RS***	***ROA***	***RRS***	***RROA***	
1	1639	1639(-)	1625	1639 (+)	1619s	1622(-)	1618s	1621(+)	C=C str (5-6), C=N str (4-21)
2	1613	1612(+)	1611	-	1602	1602(+)	1600	-	C=C str (14-15), C=N str (16-24)
3	1574	1575(+)	1573	1572(+)	1578	1577(+)	1574	1577(+)	C=C str (5-6;10-11), C-H bend (10), C=N str (16-24;4-21), NH_2 bend (scissoring), DmB C=C str
4	1551	1551(-)	1549	1549(+)	1550	1547(-)	1549	1547(+)	(ν_{SA}) C=C str (5-6;10-11;14-15), C=N str (9-22;11-23;16-24;4-21)
5	1503	1504(-)<br>1492(+)	1501	1498(+)	1503	1511(-)<br>1494(+)	1501	1501(+)	(ν_{LA}) C=C str (5-6;9-10;14-15), C=N str (4-21;11-23;16-24, B2-B1;B2-B3), C-H bend, CH_3 bend asym<br>CH_3 bend asym, C-H bend (10), CH_2 bend (scissoring), C=C str (14-15), C=N str (16-24;9-22)
6	1485s	1465(-)	1480	1476(+)	1476s	1480(-)	1476s	1480s (+)	CH_3 bend asym, CH_2 bend (scissoring)
7	1441	1444(+)<br>1433(-)	1434	1439(+)	1448	1447(+)<br>1424(-)	1447	1451(+)	CH_3 bend asym, CH_2 bend (scissoring)
8	1384	1393(+)	1380	1370(+)	1398	-	1396	1395(+)	CH_3 bend sym, CH bend
9	1352	1354(+)	1349	1336(+)	1368	1376(+)	1367	1365(+)	CH_3 bend sym, CH bend, CH_2 wagg
10	1330	1327(+)	1338	1325(+)	1344	1341(+)	1352s	1345s (+)	CH_2 twist, CH bend, DmB ring breathing
11	1287	1288(-)	1285	1285(+)	1318s	1315(-)	1324s	1326s (+)	CH bend, CH_2 twist, DmB ring breathing, CH_2 wagg
12	1267	1262(+)	1269	1269(+)	1290s	1289(+)	1287	-	CH_2 twist, CH_2 wagg, CH bend
13	1223	1223(-)<br>1207(+)	1222	1222(+)	1246	1253(-)<br>1229(+)	1244	1238(+)<br>1259(+)	CH_2 wagg, CH bend, NH bend
14	1177	1176(-)	1177	1177(+)	1202s	1202(-)	1200s	1200s (+)	CH_2 wagg, CH bend, CH_3 rock, C-N str, C-C str, CH_2 twist
15	1145	1143(+)	1162<br>1147	1166(+)<br>1146(+)	1184<br>1167	1184(+)<br>1167(+)	1184<br>1168(s)	1186(+)	CH_2 twist, CH bend, CH_3 rock, C-N str, C-C str (corr. ring)
16	1127	1124(-)	1127s	1127s(+)	1146s	1148(-)	1146s	1152(+)	CH_2 twist, CH bend, CH_3 rock (51), C-C str
17	1087	1090(+)	1086	1085(+)	1095	1096(+)	1093s	1096(+)	C-N str (6-22;14-23,1-21,65-59), C-C str, CH_2 twist, CH bend, CH_3 rock, NH_2 rock
18	1072s	1072(+)	1071s	-	1060	1058(+)	1060	-	C-C str, CH_3 rock, NH_2 rock
19	842	842(+)	840s	841s(+)	882	874(+)	876	882(+)	CH_3 rock, C-C str, Co-N str
20	720<br>709	738(+)s<br>724(+)	721	720(+)	740	746(+)	742	746(+)	CH_3 rock,CH_2 rock, corrin ring tors and bend, C-C str, NH_2 twist, DmB ring str
21	692<br>674<br>662	675(+)	689s<br>674<br>658	689(+)<br>676(+)<br>656(+)	693<br>681	672(+)	696<br>683	-	NH_2 twist, NH bend, corrin ring tors and bend
22	624	641(-)<br>624(+)s	623	625(+)	637	-	634	634(+)	NH_2 twist, corrin ring tors and bend
23	580	583(+)	570	580(+)	593	593(+)	594	593(+)	NH_2 twist, corrin ring tors and bend, DmB ring tors and bend
24	482<br>454<br>439	488(-)<br>475(+)<br>461(-)	457<br>437s	460(+)<br>436(-)	497<br>462	504(-)<br>464(+)	489	-	Co-C≡N bend, C≡N twist, DmB ring str, Co-N bend
25	414<br>401<br>389<br>373	423(-)<br>407(+)	413	474(+)	410<br>381s	426(-)<br>410(+)	434	-	Co-C≡N bend, corrin ring tors and bend, CCN, CNC bend, CC, CN tors, NH_2 twist, CH_3 tors
26	344<br>325	345(+)<br>313(-)	322	326(+)	337	369(+)<br>319(-)	333	324(+)	Co-C≡N bend, corrin ring tors and bend, CCN, CNC bend, CC, CN tors, NH_2 twist, NH_2 wagg, CH_3 tors

Abbreviations: str – stretching; bend – bending; sym – symmetric; asym – asymmetric; wagg – wagging; twist – twisting; rock– rocking; tors – torsion; s – shoulder, (+) and (–) – positive and negative ROA intensities.

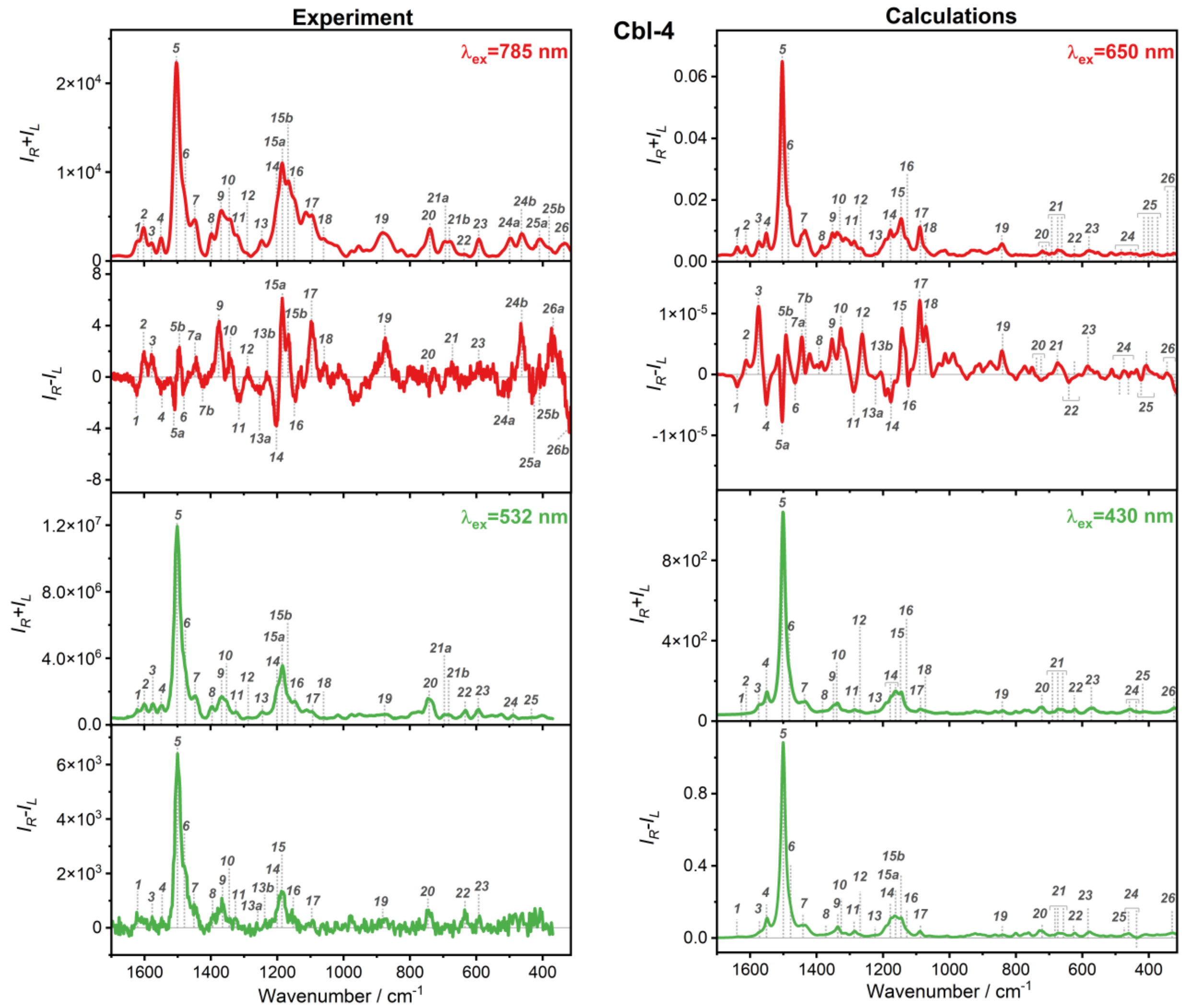

Figure S17. Experimental FFR-ROA/FFR-Raman and RROA/RRaman spectra (left panel) of **Cbl-4** in comparison with calculated (Boltzmann average, ΔG) spectra (right panel) using CAM-B3LYP-GD3/6-31G(d)/MDF10/PCM level. The experimental spectra were obtained with excitation wavelengths of 785 and 532 nm, while the calculated spectra with excitation wavelengths of 650 and 430 nm, respectively. The calculated vibrational frequencies were scaled by a factor of 0.9400 (532 nm) and 0.9410 (785 nm). The numbering of vibrational bands corresponds to the band assignments in **Table S3**.

Table S4. Circular intensity difference (CID, the ratio of ROA to Raman intensity) values, plotted for selected vibrational bands, obtained from **Cbl-1** and **Cbl-4** experimental spectra.

**Cbl-1**				**Cbl-4**			
**far from resonance**		**resonance**		**far from resonance**		**resonance**	
v / cm^{-1}	CID / 10^{-4}	*v* / cm^{-1}	CID / 10^{-4}	*v* / cm^{-1}	CID / 10^{-4}	*v* / cm^{-1}	CID / 10^{-4}
1549	-1.8	1542	9.5	1547	-2.9	1547	5.0
1504<br>1491	-2.9<br>1.3	1501	4.2	1511<br>1494	-1.6<br>1.6	1501	5.7
1183<br>1161	6.4<br>5.6	1168	2.3	1184<br>1167	5.9<br>3.5	1186	5.0
497<br>466	-126<br>13	497	-40	369	55	-	-

Table S5. Dissymmetry factor (g-factor, ECD/UV-Vis) values, plotted for selected bands, obtained from **Cbl-1** and **Cbl-4** experimental spectra.

**Cbl-1**		**Cbl-4**	
λ / nm	*g* / 10^{-4}	λ / nm	*g* / 10^{-4}
544	-2.4	577	-19
507	-0.5	468	-2.1
483	-8.5	417	31
433	50	-	-

Table S6. The relative ΔE_{ZPE} (kJ mol^{-1}), Gibbs free energies ΔG (kJ mol^{-1}), and Boltzmann populations (%), at 298 K of the most stable conformers of **Cbl-1**, calculated at CAM-B3LYP-GD3/6-31G(d)/MDF10/PCM level.

Conformer	ΔE_{ZPE}	ΔG	Population ΔE_{ZPE}	Population ΔG
1	0.00^a	0.00^b	83.8	63.4
2	5.15	2.19	10.5	26.2
3	6.67	4.49	5.7	10.4

^{a} E_{ZPE}= -12490801.16 kJ mol^{-1}, ^{b} G= -12491135.90 kJ mol^{-1}

Table S7. The relative ΔE_{ZPE} (kJ mol^{-1}), Gibbs free energies ΔG (kJ mol^{-1}), and Boltzmann populations (%), at 298 K of the most stable conformers of **Cbl-4**, calculated at CAM-B3LYP-GD3/6-31G(d)/MDF10/PCM level.

Conformer	ΔE_{ZPE}	ΔG	Population ΔE_{ZPE}	Population ΔG
1	0.00^a	0.00^b	52.3	83.4
2	1.08	18.91	33.8	0
3	4.59	5.18	8.2	10.3
4	6.25	11.41	4.2	0.8
5	8.93	6.79	1.4	5.4

^{a} E_{ZPE}= -12843463.91 kJ mol^{-1}, ^{b} G= -12843809.33kJ mol^{-1}

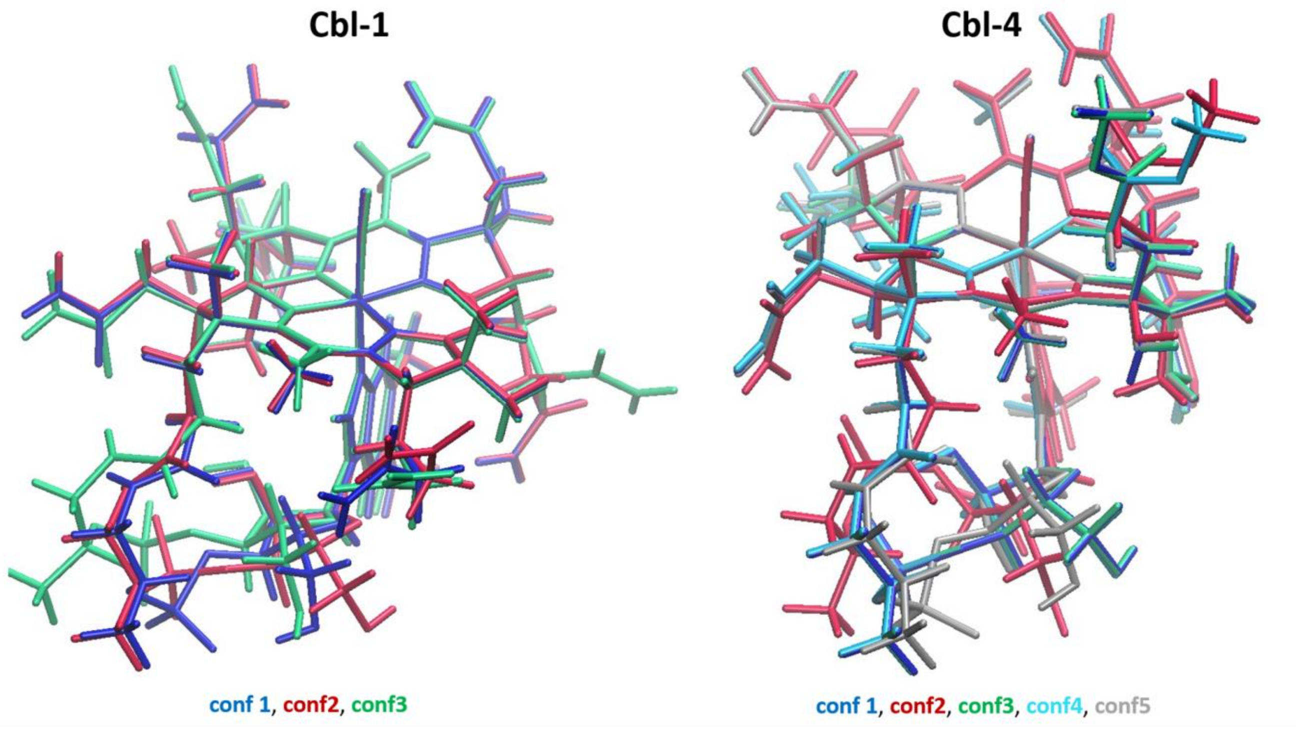

Figure S18. Aligned structures of Cbl-1 and Cbl-4 conformers, calculated at CAM-B3LYP-GD3/6-31G(d)/MDF10/PCM level.

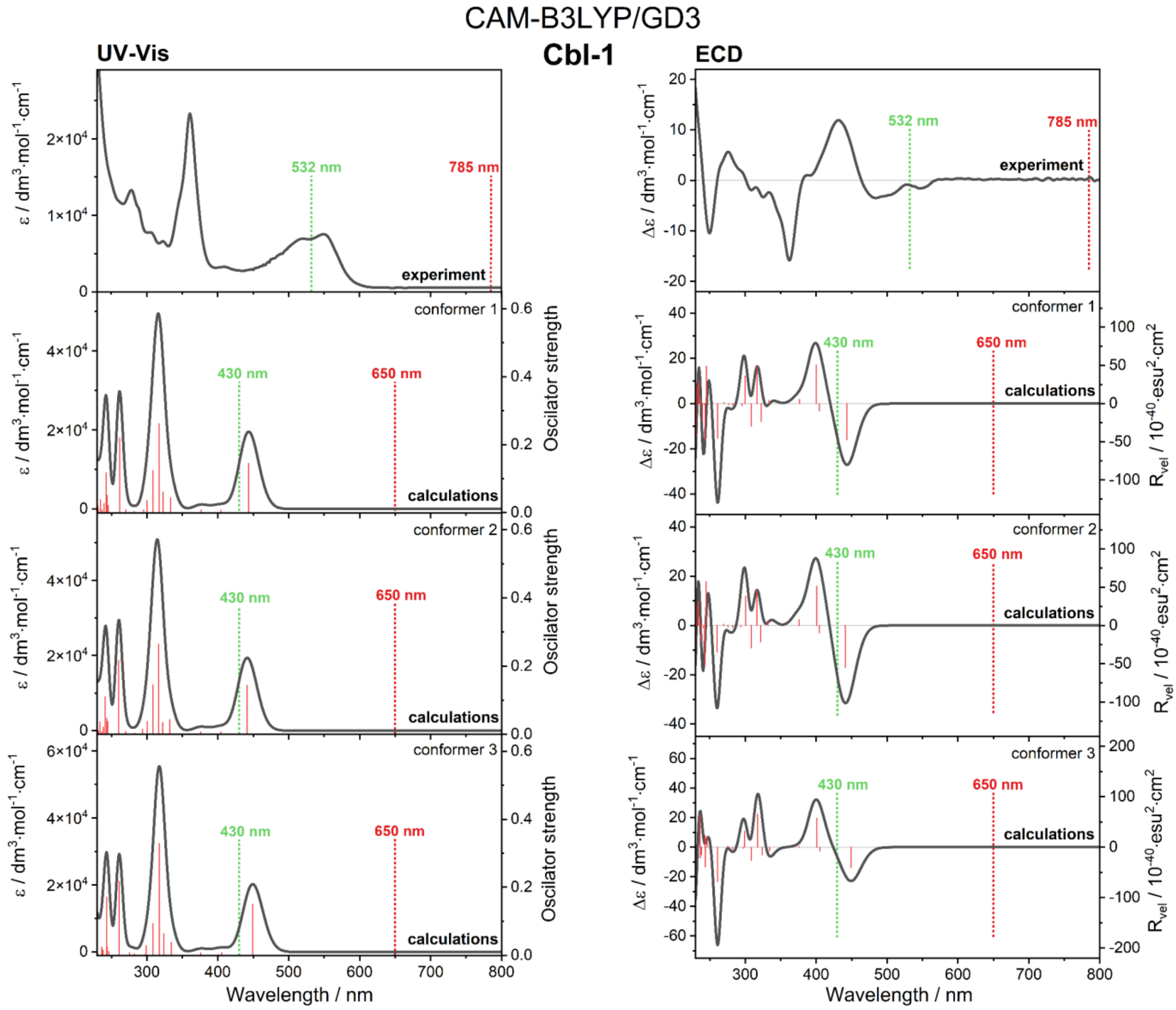

Figure S19. Comparison of experimental and calculated UV-Vis (left panel) and ECD (right panel) spectra of **Cbl-1** conformers, calculated at CAM-B3LYP-GD3/6-31G(d)/MDF10/PCM level. Individual transition energies and their oscillatory and rotatory strengths are indicated by the red vertical lines. The 532 and 785 nm excitation wavelengths and the two used in the calculations (430 and 650 nm) are indicated by the green and red dotted lines, respectively.

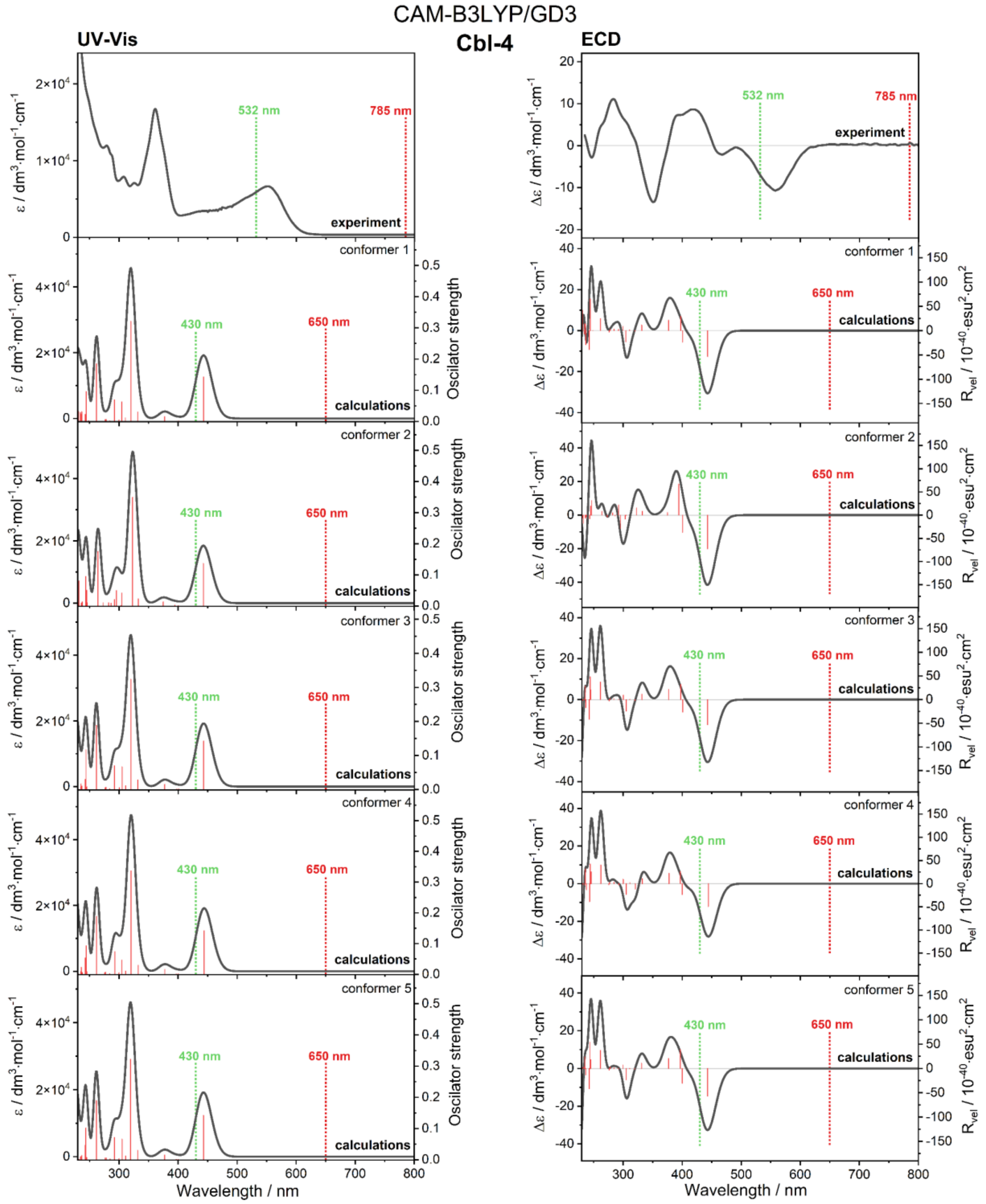

Figure S20. Comparison of experimental and calculated UV-Vis (left panel) and ECD (right panel) spectra of **Cbl-4** conformers, calculated at CAM-B3LYP-GD3/6-31G(d)/MDF10/PCM level. Individual transition energies and their oscillatory and rotatory strengths are indicated by the red vertical lines. The 532 and 785 nm excitation wavelengths and the two used in the calculations (430 and 650 nm) are indicated by the green and red dotted lines, respectively.

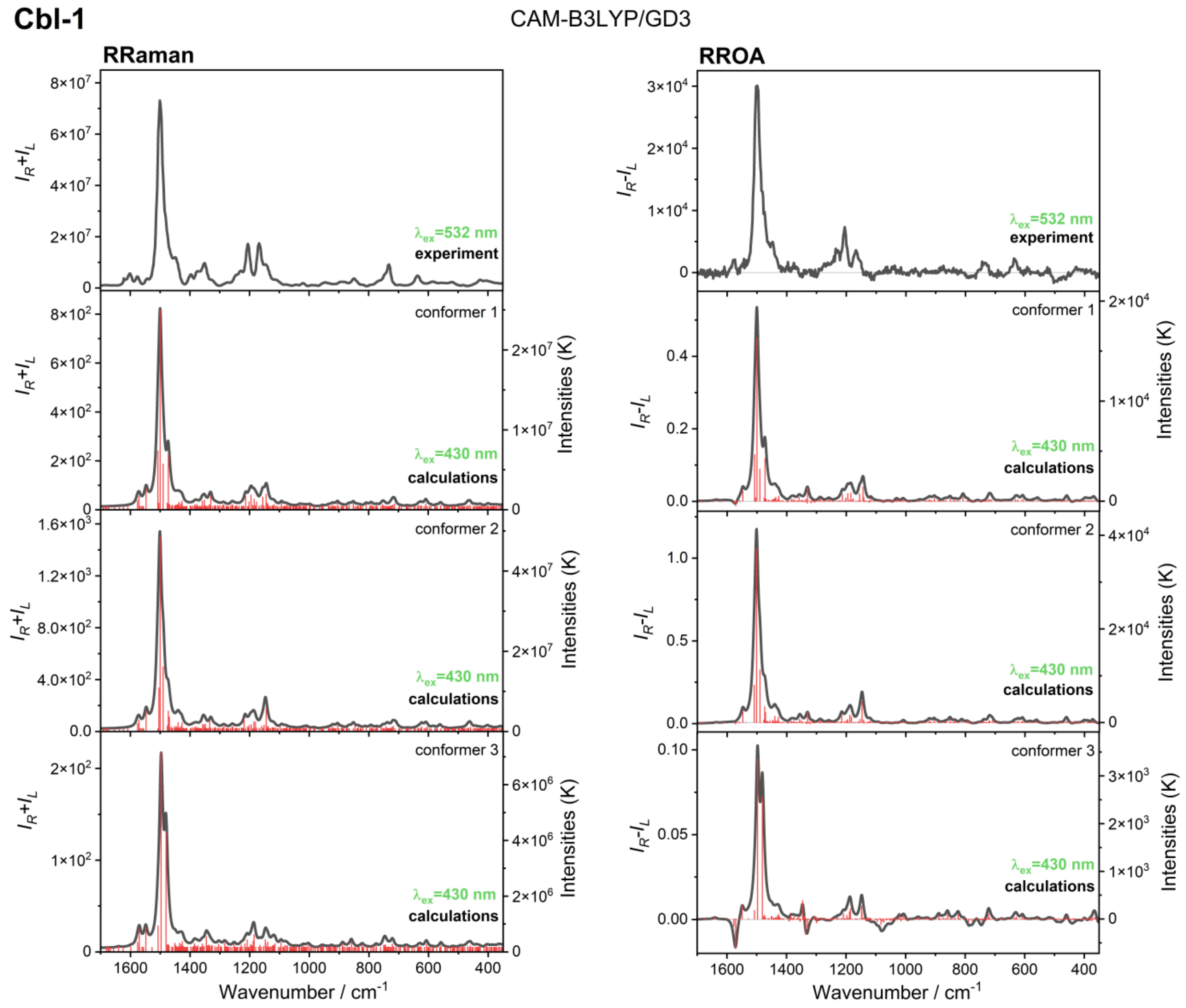

Figure S21. Comparison of experimental and calculated RRaman (left panel) and RROA (right panel) spectra of **Cbl-1** conformers, calculated at CAM-B3LYP-GD3/6-31G(d)/MDF10/PCM level. Red vertical lines represent computed frequencies and intensities. The experimental spectra were obtained with an excitation wavelength of 532 nm, while the calculated spectra with an excitation wavelength of 430 nm. All calculated vibrational frequencies were scaled by a factor of 0.9410.

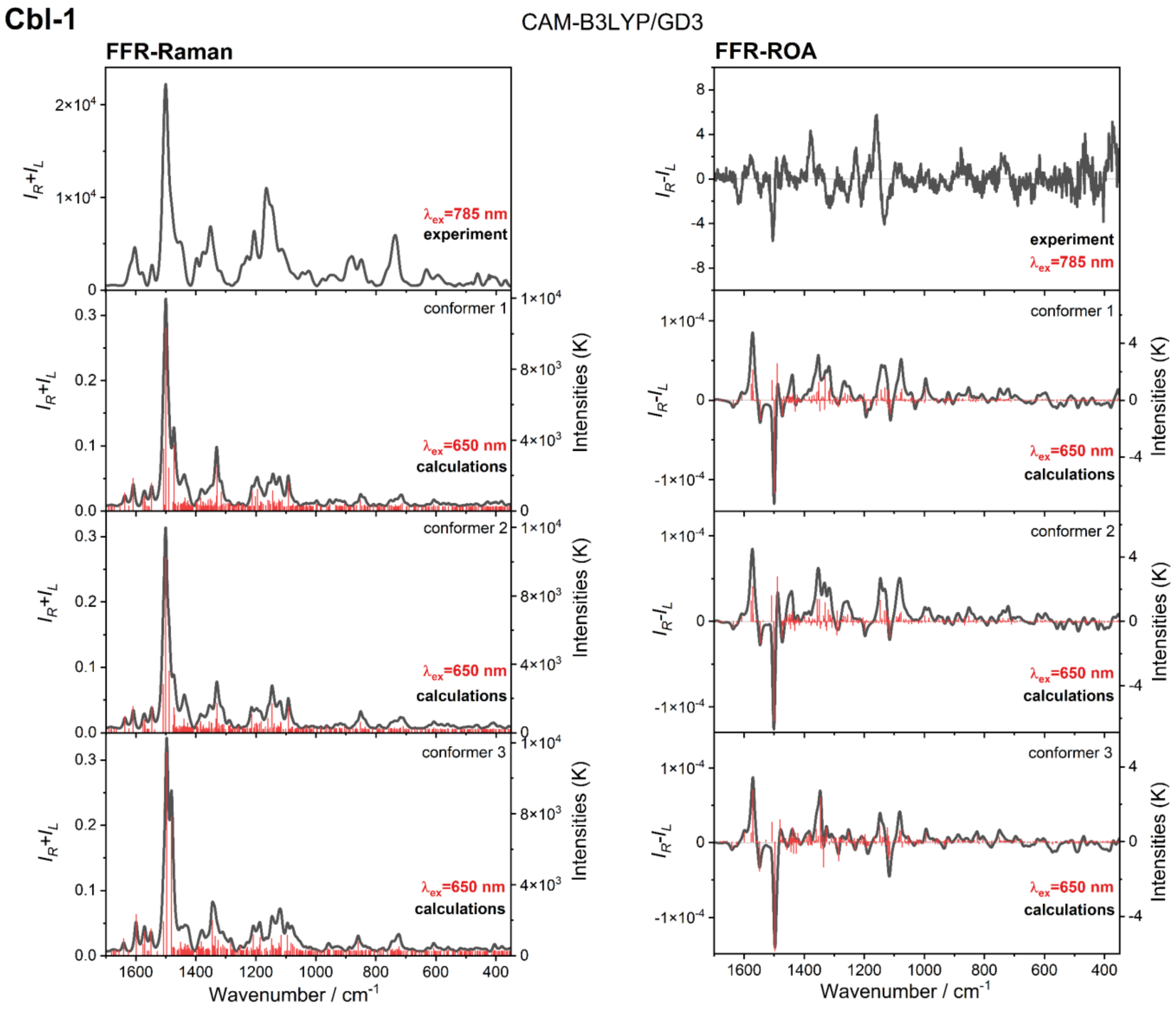

Figure S22. Comparison of experimental and calculated FFR-Raman (left panel) and FFR-ROA (right panel) spectra of **Cbl-1** conformers, calculated at CAM-B3LYP-GD3/6-31G(d)/MDF10/PCM level. Red vertical lines represent computed frequencies and intensities. The experimental spectra were obtained with an excitation wavelength of 785 nm, while the calculated spectra with an excitation wavelength of 650 nm. All calculated vibrational frequencies were scaled by a factor of 0.9410.

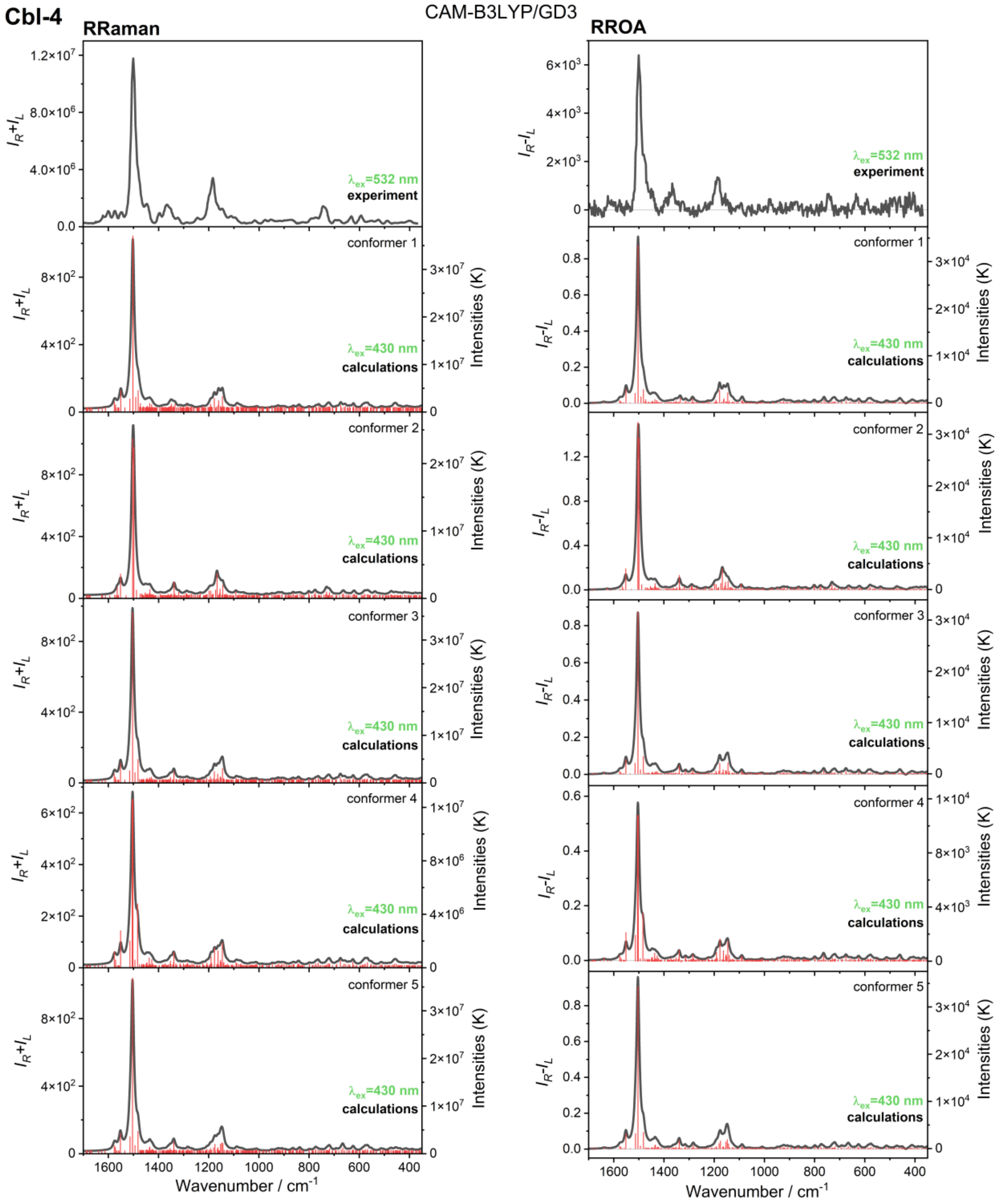

Figure S23. Comparison of experimental and calculated RRaman (left panel) and RROA (right panel) spectra of **Cbl-4** conformers, calculated at CAM-B3LYP-GD3/6-31G(d)/MDF10/PCM level. Red vertical lines represent computed frequencies and intensities. The experimental spectra were obtained with an excitation wavelength of 532 nm, while the calculated spectra with an excitation wavelength of 430 nm. All calculated vibrational frequencies were scaled by a factor of 0.9400.

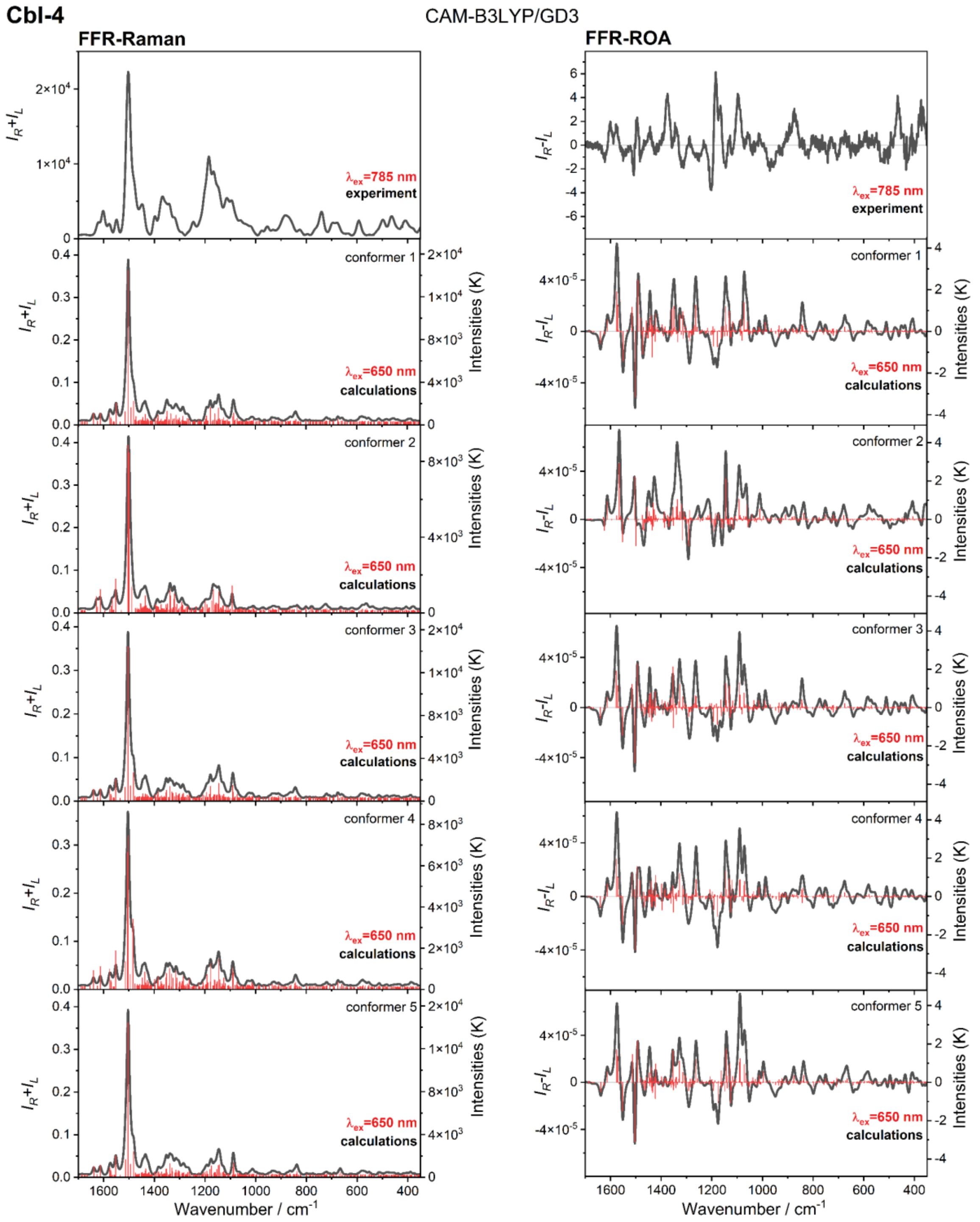

Figure S24. Comparison of experimental and calculated FFR-Raman (left panel) and FFR-ROA (right panel) spectra of **Cbl-4** conformers, calculated at CAM-B3LYP-GD3/6-31G(d)/MDF10/PCM level. Red vertical lines represent computed frequencies and intensities. The experimental spectra were obtained with an excitation wavelength of 785 nm, while the calculated spectra with an excitation wavelength of 650 nm. All calculated vibrational frequencies were scaled by a factor of 0.9410.

Table S8. Cartesian coordinates of Cbl-1 conformers optimized at CAM-B3LYP-GD3/6-31G(d)/MDF10/PCM theory level.

Conformer 1				Conformer 2				Conformer 3			
Co	0.002796	-0.105663	0.067178	Co	0.004649	-0.106873	0.074837	Co	0.006222	-0.109058	0.071168
P	-5.284781	-4.700082	5.922376	P	-4.438109	-4.896788	5.300196	P	-3.416244	-5.755816	4.908492
C	0.831301	-2.864927	-0.078617	C	0.826758	-2.863072	-0.091615	C	0.836127	-2.870242	-0.111638
C	2.020998	-3.618447	-0.803483	C	2.018599	-3.615270	-0.813219	C	2.022787	-3.624928	-0.846233
C	3.232914	-2.690915	-0.469056	C	3.231640	-2.696371	-0.459982	C	3.231944	-2.676958	-0.551523
H	3.945993	-2.697989	-1.293808	H	3.953603	-2.702125	-1.276823	H	3.904893	-2.685520	-1.405445
C	2.563641	-1.342139	-0.375917	C	2.566892	-1.345012	-0.365979	C	2.557482	-1.336845	-0.417469
C	3.275629	-0.090607	-0.551326	C	3.282310	-0.095190	-0.536796	C	3.264096	-0.075869	-0.569441
C	2.702802	1.092289	-0.189905	C	2.707509	1.088934	-0.181792	C	2.698713	1.098555	-0.169443
C	3.311135	2.504894	-0.230227	C	3.314723	2.502107	-0.229383	C	3.310608	2.509423	-0.175560
C	2.432915	3.199041	0.851123	C	2.430758	3.203672	0.841910	C	2.418474	3.208085	0.892563
H	2.304691	4.264722	0.642461	H	2.302208	4.267597	0.624596	H	2.271292	4.263519	0.658225
C	1.144581	2.448833	0.690818	C	1.144179	2.450957	0.681365	C	1.139681	2.437647	0.743084
C	-0.103510	3.008833	0.915518	C	-0.104641	3.012880	0.891783	C	-0.110670	2.989813	0.995241
H	-0.122606	4.044419	1.225621	H	-0.125100	4.052957	1.186211	H	-0.111530	4.015799	1.337456
C	-1.317173	2.386740	0.660312	C	-1.319201	2.387718	0.645001	C	-1.317507	2.366785	0.735370
C	-2.643421	3.120391	0.659975	C	-2.640355	3.126878	0.634346	C	-2.654109	3.085801	0.756042
C	-3.642170	1.937312	0.706258	C	-3.646667	1.950989	0.687159	C	-3.625082	1.881575	0.821162
H	-4.484789	2.146064	0.047280	H	-4.455875	2.144560	-0.018051	H	-4.529369	2.079732	0.241443
C	-2.806545	0.777937	0.191297	C	-2.812600	0.772711	0.209409	C	-2.792619	0.769267	0.208824
C	-3.330154	-0.431057	-0.185680	C	-3.335599	-0.437552	-0.163668	C	-3.319072	-0.405814	-0.263860
C	-2.476376	-1.574342	-0.436468	C	-2.476389	-1.572622	-0.440003	C	-2.475260	-1.552322	-0.504261
C	-2.911110	-2.955878	-0.961619	C	-2.899597	-2.932252	-1.025807	C	-2.905074	-2.935221	-1.032386
C	-1.625481	-3.789292	-0.662459	C	-1.621277	-3.774288	-0.724376	C	-1.640355	-3.766023	-0.649028
H	-1.664291	-4.084521	0.387454	H	-1.675920	-4.086497	0.319952	H	-1.707531	-3.972892	0.420917
C	-0.527958	-2.743277	-0.811912	C	-0.523208	-2.726873	-0.836862	C	-0.529186	-2.742111	-0.832032
H	-0.334829	-2.551384	-1.869713	H	-0.317478	-2.508479	-1.886971	H	-0.338622	-2.562839	-1.892711
C	0.593725	-3.356374	1.352614	C	0.570423	-3.366939	1.333013	C	0.616793	-3.374382	1.315234
H	-0.260344	-2.855006	1.803937	H	1.428540	-3.193646	1.980889	H	0.373710	-4.438871	1.305515
H	1.459480	-3.175092	1.988015	H	0.355814	-4.436880	1.321945	H	-0.211064	-2.861715	1.795386
H	0.383266	-4.427340	1.354568	H	-0.288886	-2.865404	1.776463	H	1.502419	-3.227857	1.932895
C	2.213097	-5.064729	-0.349846	C	2.196400	-5.067399	-0.372444	C	2.235035	-5.062119	-0.364952
H	2.992238	-5.535504	-0.948422	H	2.973813	-5.538832	-0.972794	H	1.337105	-5.668132	-0.507325
H	1.301102	-5.649674	-0.490958	H	1.278944	-5.642283	-0.520556	H	2.496618	-5.115964	0.691939
H	2.482795	-5.134481	0.704528	H	2.461276	-5.148832	0.682448	H	3.038070	-5.525501	-0.937605
C	1.822441	-3.561966	-2.343933	C	1.837444	-3.540131	-2.355264	C	1.794831	-3.632032	-2.371645
H	1.030212	-4.254063	-2.641848	H	1.053864	-4.232523	-2.674456	H	0.879540	-4.178805	-2.614467
H	1.506056	-2.558281	-2.639378	H	1.517860	-2.535125	-2.642119	H	1.645391	-2.608330	-2.738011
C	3.059476	-3.941507	-3.144603	C	3.088098	-3.898544	-3.144795	C	2.917394	-4.233695	-3.215141
C	4.039001	-2.952047	0.822735	C	4.024404	-2.969384	0.837255	C	4.082454	-2.944839	0.709709
H	4.445770	-2.003571	1.185736	H	4.436662	-2.025982	1.206903	H	4.482769	-1.996354	1.080214
H	3.399700	-3.312928	1.627563	H	3.374897	-3.327214	1.635516	H	3.473495	-3.329861	1.527508
C	5.201023	-3.916265	0.625262	C	5.179380	-3.942792	0.643560	C	5.253802	-3.885375	0.460674
H	4.857704	-4.878384	0.231728	H	4.829467	-4.903575	0.252954	H	4.914781	-4.839129	0.045749
H	5.906168	-3.519520	-0.114190	H	5.886972	-3.553579	-0.097738	H	5.932854	-3.459992	-0.286731
C	5.936507	-4.177117	1.930619	C	5.916417	-4.202718	1.948086	C	6.022427	-4.171956	1.739932
C	4.650058	-0.207429	-1.169257	C	4.659778	-0.215617	-1.147148	C	4.630201	-0.179786	-1.208015
H	5.044884	0.749476	-1.489256	H	4.619791	-0.836958	-2.046731	H	5.028108	0.783061	-1.504069
H	4.608422	-0.836115	-2.063433	H	5.376284	-0.678800	-0.460025	H	4.572038	-0.780032	-2.120459
H	5.374325	-0.660570	-0.483567	H	5.063026	0.741119	-1.456608	H	5.360614	-0.659970	-0.548042
C	4.804218	2.664052	0.074740	C	4.806097	2.666552	0.081447	C	4.799942	2.647938	0.150433
H	4.982861	3.684324	0.427000	H	4.980559	3.689432	0.428292	H	4.993441	3.671557	0.485047
H	5.418638	2.529451	-0.812016	H	5.424915	2.528313	-0.801728	H	5.428773	2.484734	-0.720958
H	5.139732	1.972588	0.850302	H	5.139815	1.980684	0.862759	H	5.105416	1.964263	0.946048
C	2.952763	3.150548	-1.602998	C	2.962292	3.136287	-1.609103	C	2.970866	3.185062	-1.539296
H	1.866190	3.150703	-1.733283	H	1.876343	3.134622	-1.744584	H	1.885063	3.194635	-1.676349
H	3.287905	4.190882	-1.577375	H	3.296846	4.176914	-1.590344	H	3.310999	4.222845	-1.487232
C	3.630718	2.498703	-2.797540	C	3.646552	2.474684	-2.794654	C	3.650823	2.560809	-2.745534
C	2.990594	3.020009	2.278698	C	2.981299	3.036866	2.273910	C	2.950603	3.087116	2.336298
H	3.345822	1.992814	2.402882	H	3.341794	2.012676	2.407488	H	3.139848	2.034683	2.566085
H	3.854725	3.674562	2.405723	H	3.840537	3.697953	2.401232	H	3.910829	3.604700	2.401274
C	1.993166	3.322263	3.397359	C	1.974561	3.340408	3.384392	C	2.021197	3.642553	3.427228
H	1.564596	4.321641	3.261613	H	1.533130	4.331764	3.233445	H	1.045473	3.152146	3.382012
H	1.165174	2.609111	3.387224	H	1.157276	2.614864	3.379848	H	2.457806	3.409177	4.402180
C	2.709394	3.317688	4.736392	C	2.686019	3.364407	4.725466	C	1.793437	5.134658	3.281071
C	-2.763836	3.821643	-0.709385	C	-2.765416	3.822073	-0.737345	C	-2.809612	3.785903	-0.609627
H	-2.687763	3.101039	-1.529991	H	-2.682613	3.100184	-1.556515	H	-2.735719	3.068972	-1.433641
H	-1.976358	4.570094	-0.833877	H	-1.985452	4.578019	-0.864159	H	-2.035112	4.545990	-0.744083
H	-3.734341	4.321771	-0.780174	H	-3.743649	4.307479	-0.798330	H	-3.787971	4.272744	-0.664309
C	-2.770548	4.164979	1.767746	C	-2.764411	4.179395	1.734076	C	-2.774483	4.126350	1.867435
H	-3.748705	4.648735	1.717044	H	-3.750527	4.642729	1.674118	H	-3.751881	4.613463	1.821883
H	-2.022038	4.950980	1.639555	H	-2.018732	4.966507	1.594817	H	-2.020119	4.907344	1.741793
H	-2.642134	3.739944	2.765609	H	-2.622898	3.756496	2.732216	H	-2.643980	3.695726	2.862733
C	-4.199648	1.567205	2.102217	C	-4.263168	1.608262	2.062339	C	-4.022792	1.426833	2.247429
H	-3.437252	1.718848	2.870639	H	-3.483279	1.546681	2.827455	H	-3.168585	1.530615	2.921756
H	-4.425283	0.496809	2.122852	H	-4.687625	0.603639	1.990932	H	-4.265728	0.362159	2.227265
C	-5.482719	2.321497	2.491874	C	-5.376164	2.558242	2.552798	C	-5.240021	2.143639	2.833501
H	-5.696863	2.148105	3.550371	H	-5.972644	2.036716	3.306754	H	-5.338145	1.857340	3.885996
H	-5.365356	3.396223	2.350278	H	-4.963259	3.448937	3.021780	H	-5.131363	3.228469	2.800813
C	-6.636267	1.867245	1.619079	C	-6.254857	3.021879	1.408622	C	-6.509888	1.687542	2.137850
C	-4.832949	-0.568632	-0.308825	C	-4.836269	-0.588286	-0.306147	C	-4.814198	-0.454141	-0.505218
H	-5.323702	0.402335	-0.317638	H	-5.097203	-0.983494	-1.290515	H	-5.089761	-1.268547	-1.168567
H	-5.272772	-1.151386	0.506670	H	-5.347183	0.368651	-0.219307	H	-5.148160	0.462729	-0.997155
H	-5.107878	-1.053094	-1.247684	H	-5.276003	-1.259128	0.437907	H	-5.402707	-0.551783	0.412657
C	-3.170519	-2.811351	-2.473711	C	-3.110963	-2.715240	-2.538281	C	-3.088145	-2.815036	-2.557690
H	-2.278194	-2.485260	-3.015749	H	-3.891754	-1.972950	-2.717090	H	-3.414314	-3.770459	-2.977971
H	-3.959213	-2.081064	-2.665700	H	-3.430241	-3.645460	-3.015216	H	-2.167940	-2.514413	-3.066089
H	-3.497814	-3.765423	-2.895532	H	-2.202970	-2.367750	-3.039319	H	-3.854748	-2.073755	-2.795704
C	-4.149656	-3.574307	-0.294052	C	-4.155897	-3.589515	-0.437683	C	-4.174204	-3.568575	-0.429904
H	-5.039087	-3.016032	-0.578539	H	-5.047749	-3.051503	-0.752876	H	-5.068838	-3.169530	-0.911866
H	-4.287220	-4.576138	-0.709180	H	-4.241560	-4.583366	-0.889657	H	-4.160994	-4.625830	-0.703442
C	-4.067009	-3.675981	1.234656	C	-4.168118	-3.735076	1.083180	C	-4.316546	-3.421330	1.084075

H	-3.460501	-4.543048	1.506386	H	-3.453666	-4.496864	1.397943	H	-3.381984	-3.664113	1.599540
H	-3.592638	-2.786492	1.655071	H	-3.883362	-2.797770	1.571517	H	-4.572781	-2.390705	1.325116
C	-5.456560	-3.766024	1.822822	C	-5.563694	-4.082715	1.558641	C	-5.415599	-4.291661	1.665711
C	-1.444439	-5.063254	-1.478235	C	-1.436865	-5.037759	-1.555996	C	-1.474837	-5.115162	-1.336412
H	-1.559969	-4.886953	-2.550025	H	-1.608988	-4.863036	-2.620175	H	-1.683930	-5.073624	-2.407914
H	-0.427217	-5.436747	-1.335513	H	-0.403607	-5.381060	-1.464390	H	-0.438057	-5.446893	-1.238516
C	-2.351856	-6.181705	-0.986035	C	-2.281644	-6.185738	-1.021644	C	-2.305323	-6.186770	-0.643593
C	0.043612	0.246781	-1.781106	C	0.044274	0.253663	-1.772760	C	0.046576	0.295460	-1.768209
C	-7.196560	-5.231049	2.713894	C	-6.890975	-5.615675	2.918951	C	-6.000575	-6.572121	2.309330
H	-7.419409	-6.298944	2.651455	H	-6.943238	-6.706880	2.981604	H	-5.803389	-7.567440	1.900454
H	-7.955736	-4.686311	2.146142	H	-7.731447	-5.260175	2.319840	H	-7.055020	-6.335908	2.148430
C	-7.239710	-4.775646	4.165394	C	-6.984275	-5.026563	4.322217	C	-5.698834	-6.563838	3.806214
H	-7.009853	-3.709059	4.194631	H	-6.886601	-3.940297	4.256322	H	-5.989129	-5.589231	4.205233
C	-8.581518	-5.036262	4.821135	C	-8.282975	-5.400074	5.009585	C	-6.404317	-7.678246	4.553460
H	-8.808325	-6.107197	4.814926	H	-8.372741	-6.487978	5.090087	H	-6.091259	-8.653187	4.166389
H	-9.381910	-4.505366	4.296439	H	-9.138257	-5.020860	4.442817	H	-7.489868	-7.591545	4.442584
H	-8.547949	-4.689192	5.856084	H	-8.314333	-4.974782	6.015620	H	-6.155594	-7.628996	5.616204
C	-2.001305	-1.117617	5.008159	C	-1.981954	-1.039710	5.030765	C	-1.940734	-1.358492	4.982524
H	-1.667036	-0.793138	5.997361	H	-1.571772	-0.824584	6.020921	H	-1.659183	-1.001047	5.976163
C	-2.252384	-2.634221	4.978200	C	-2.469192	-2.492014	4.940239	C	-2.026187	-2.900429	4.928838
H	-1.651182	-3.160011	5.727377	H	-1.872592	-3.149141	5.582846	H	-1.483711	-3.348804	5.768274
C	-3.746025	-2.674377	5.263406	C	-3.907103	-2.316241	5.447022	C	-3.546206	-3.127091	5.023012
H	-3.919754	-2.497953	6.327327	H	-3.901528	-2.203907	6.536732	H	-3.878067	-3.111819	6.065673
C	-4.256281	-1.466657	4.486937	C	-4.312626	-0.999939	4.796975	C	-4.090385	-1.915679	4.279694
H	-4.333389	-1.705821	3.422007	H	-4.641380	-1.192404	3.769372	H	-4.000987	-2.105596	3.209268
C	-5.601667	-0.933491	4.960696	C	-5.365063	-0.225181	5.564243	C	-5.519908	-1.550627	4.583847
H	-5.669514	0.116662	4.655804	H	-5.635696	0.682080	5.008498	H	-5.799440	-0.653320	4.016674
H	-6.376669	-1.492277	4.417575	H	-6.260675	-0.844876	5.660218	H	-6.137941	-2.378932	4.213212
C	-1.182047	-0.530521	2.737888	C	-1.164521	-0.540088	2.746043	C	-1.156670	-0.633634	2.735964
H	-2.167164	-0.528912	2.304889	H	-2.151832	-0.544741	2.318296	H	-2.141179	-0.650308	2.302509
C	2.351980	-0.431121	2.891504	C	2.370897	-0.431072	2.888420	C	2.375439	-0.475194	2.872545
H	2.818802	-0.253351	1.935831	H	2.833887	-0.253198	1.931234	H	2.835336	-0.257402	1.921615
C	3.140629	-0.616190	4.015171	C	3.164829	-0.606518	4.010004	C	3.173555	-0.705841	3.981444
C	2.544798	-0.859711	5.282543	C	2.574789	-0.847849	5.280172	C	2.588179	-1.009421	5.240980
C	1.163563	-0.905109	5.411020	C	1.194091	-0.899180	5.413276	C	1.208988	-1.069028	5.375963
H	0.707066	-1.098905	6.375688	H	0.742396	-1.092463	6.380280	H	0.760405	-1.309347	6.334090
C	0.390350	-0.710640	4.266997	C	0.415949	-0.713830	4.271253	C	0.426269	-0.833871	4.247387
C	0.963594	-0.480477	3.011898	C	0.983147	-0.487341	3.012989	C	0.988313	-0.544720	2.999842
C	4.640011	-0.578354	3.874842	C	4.663553	-0.560513	3.864768	C	4.672480	-0.668706	3.826953
H	5.068337	-1.583048	3.961074	H	5.098271	-1.561677	3.958447	H	4.951572	-0.216702	2.870629
H	5.102627	0.054165	4.638568	H	5.124382	0.081560	4.621608	H	5.094799	-1.679844	3.845560
H	4.927207	-0.184347	2.895815	H	4.945450	-0.173328	2.881493	H	5.150934	-0.089890	4.622825
C	3.416289	-1.057047	6.493603	C	3.451189	-1.038811	6.488777	C	3.470078	-1.277322	6.431828
H	3.986252	-0.148783	6.720688	H	4.020897	-0.129174	6.710593	H	4.062295	-0.394308	6.697220
H	4.145230	-1.858177	6.333846	H	4.180583	-1.839532	6.328860	H	4.180564	-2.085061	6.226051
H	2.820220	-1.308503	7.373769	H	2.859001	-1.288163	7.372176	H	2.878798	-1.559917	7.306008
N	-0.980936	-0.713269	4.057611	N	-0.955886	-0.722920	4.065646	N	-0.945803	-0.850129	4.048517
N	-0.059790	-0.367790	2.071423	N	-0.045262	-0.377651	2.076212	N	-0.042560	-0.419650	2.069322
N	1.311071	-1.456072	-0.095823	N	1.312475	-1.456391	-0.092999	N	1.310522	-1.458717	-0.123247
N	1.388821	1.208545	0.266370	N	1.392504	1.206428	0.270674	N	1.386966	1.207013	0.296670
N	-1.467434	1.108398	0.300162	N	-1.471580	1.103131	0.310871	N	-1.461319	1.094017	0.341192
N	-1.188694	-1.529151	-0.298830	N	-1.189409	-1.526048	-0.299388	N	-1.187954	-1.521177	-0.328987
N	3.569481	-2.953115	-3.917508	N	3.577568	-2.906132	-3.925599	N	2.549387	-4.528728	-4.480796
H	4.354733	-3.166128	-4.517447	H	4.370661	-3.105879	-4.519741	H	3.253905	-4.853436	-5.127843
H	3.101841	-2.068569	-4.047584	H	3.091321	-2.032964	-4.063989	H	1.626888	-4.329710	-4.837874
N	7.112146	-4.831914	1.805913	N	7.066928	-4.901407	1.826230	N	7.222770	-4.770230	1.567093
H	7.617674	-5.092766	2.640381	H	7.572472	-5.161292	2.660946	H	7.746413	-5.058854	2.381024
H	7.449974	-5.167277	0.916416	H	7.382333	-5.268268	0.940947	H	7.551215	-5.061724	0.658831
N	2.871041	1.666466	-3.535823	N	2.890689	1.635908	-3.529479	N	2.886007	1.761165	-3.514064
H	3.314617	1.182615	-4.304644	H	3.338065	1.145408	-4.291857	H	3.331657	1.288024	-4.288237
H	1.914801	1.414334	-3.303552	H	1.931680	1.389994	-3.301868	H	1.930807	1.501554	-3.284358
N	2.212185	2.478894	5.673768	N	2.227750	2.498713	5.658123	N	2.416443	5.920695	4.182644
H	2.712086	2.387576	6.547483	H	2.729156	2.427975	6.532845	H	2.321725	6.923787	4.106489
H	1.527088	1.770992	5.453384	H	1.571830	1.764062	5.436379	H	2.988616	5.548322	4.924707
N	-7.439112	0.916277	2.142536	N	-7.223258	2.159603	1.024644	N	-7.283947	2.653398	1.603639
H	-8.172407	0.524077	1.568718	H	-7.833004	2.416501	0.260787	H	-8.151610	2.394281	1.154989
H	-7.314194	0.547117	3.072414	H	-7.448483	1.331576	1.555372	H	-7.059085	3.633347	1.681833
N	-5.900684	-5.006596	2.103730	N	-5.664032	-5.242615	2.246594	N	-5.180054	-5.621148	1.589711
H	-5.241110	-5.769975	2.085496	H	-4.802182	-5.588584	2.667153	H	-4.236719	-5.918995	1.351535
N	-2.897991	-6.960429	-1.941151	N	-2.865541	-6.971189	-1.949194	N	-2.846681	-7.126072	-1.434912
H	-3.448448	-7.760962	-1.662876	H	-3.383608	-7.784064	-1.646100	H	-3.363188	-7.886148	-1.013713
H	-2.691545	-6.839939	-2.921192	H	-2.744575	-6.822114	-2.939363	H	-2.716648	-7.131218	-2.435561
N	0.100668	0.510006	-2.912411	N	0.103263	0.520705	-2.903088	N	0.107863	0.601748	-2.888582
O	-4.327986	-3.882037	4.826273	O	-4.822891	-3.313493	5.057764	O	-3.982257	-4.299330	4.371189
O	-6.216686	-5.500114	4.866580	O	-5.916226	-5.540326	5.139937	O	-4.276134	-6.718443	3.927483
O	-4.466883	-5.701712	6.663075	O	-3.545236	-5.305634	4.136611	O	-1.976328	-5.878068	4.465793
O	-6.091295	-3.653380	6.652908	O	-3.958522	-5.121630	6.693945	O	-3.787146	-5.928191	6.347398
O	3.576595	-5.052054	-3.088365	O	3.632804	-4.994966	-3.073462	O	4.060892	-4.427923	-2.811478
O	5.495470	-3.824572	3.020797	O	5.499438	-3.811256	3.034349	O	5.588727	-3.888543	2.853374
O	4.812960	2.718929	-3.061217	O	4.830179	2.692586	-3.053874	O	4.837236	2.776784	-2.994348
O	3.675062	4.044280	4.947995	O	3.617141	4.133638	4.941243	O	1.103328	5.605817	2.375770
O	-6.788748	2.295731	0.475874	O	-6.045851	4.077941	0.816337	O	-6.800704	0.494635	2.060470
O	-6.162254	-2.763927	1.971294	O	-6.530616	-3.366597	1.290113	O	-6.447105	-3.819784	2.144283
O	-2.555161	-6.363008	0.211887	O	-2.398571	-6.385379	0.184050	O	-2.456454	-6.185738	0.580949
O	-3.219389	-0.486621	4.687114	O	-3.107238	-0.215889	4.786937	O	-3.205767	-0.845677	4.649035
O	-1.991596	-3.125226	3.681038	O	-2.438076	-2.906169	3.600814	O	-1.525863	-3.359776	3.702716
H	-2.543575	-3.919457	3.578844	H	-2.786250	-3.835903	3.602227	H	-1.581358	-4.349720	3.770217
O	-5.790234	-0.987145	6.351363	O	-4.921700	0.088277	6.868531	O	-5.658980	-1.356521	5.982069
H	-5.967472	-1.935730	6.566207	H	-4.087719	0.573014	6.760917	H	-6.601402	-1.255783	6.174355

Table S9. Cartesian coordinates of Cbl-4 conformers optimized at CAM-B3LYP-GD3/6-31G(d)/MDF10/PCM theory level.

Conformer 1	Conformer 2	Conformer 3	Confomer 4	Confomer 5
Co 0.011025 -0.099370 0.058866	Co 0.017056 -0.107325 0.095189	Co 0.010813 -0.099453 0.058796	Co 0.009307 -0.099660 0.054921	Co 0.009386 -0.098754 0.056229
C -0.138884 0.016593 -1.816200	C -0.176291 -0.012000 -1.775891	C -0.137684 0.017592 -1.816470	C -0.137703 0.011327 -1.821096	C -0.137817 0.014804 -1.819383
N -0.218847 0.149848 -2.968730	N -0.311628 0.096796 -2.925668	N -0.216399 0.152096 -2.968957	N -0.216294 0.140407 -2.974216	N -0.215043 0.146154 -2.972307
C 0.995656 -2.797921 0.212320	C 1.020460 -2.794886 0.290183	C 0.995123 -2.797606 0.208896	C 0.999341 -2.797153 0.215310	C 0.993993 -2.796103 0.214115
C 2.156976 -3.590768 -0.519365	C 2.162694 -3.580237 -0.477910	C 2.156846 -3.589864 -0.522574	C 2.157896 -3.591034 -0.519984	C 2.162128 -3.586279 -0.509399
C 3.325380 -2.558449 -0.480676	C 3.340112 -2.551736 -0.422438	C 3.325835 -2.558604 -0.479821	C 3.324142 -2.556049 -0.493226	C 3.334539 -2.557112 -0.442179
H 3.917311 -2.661475 -1.388273	H 3.975326 -2.693219 -1.294877	H 3.919510 -2.660439 -1.386374	H 3.906912 -2.660021 -1.406669	H 3.953298 -2.663626 -1.331202
C 2.582283 -1.249992 -0.492398	C 2.581752 -1.245313 -0.496323	C 2.583492 -1.249688 -0.491037	C 2.580443 -1.248447 -0.499054	C 2.586950 -1.249857 -0.478544
C 3.192300 0.002410 -0.900362	C 3.118032 0.003395 -1.020228	C 3.194815 0.002197 -0.897241	C 3.190011 0.004312 -0.905374	C 3.193050 0.000324 -0.898172
C 2.597480 1.189765 -0.596891	C 2.528633 1.191429 -0.701382	C 2.599282 1.189387 -0.594119	C 2.595934 1.191117 -0.597830	C 2.595869 1.188973 -0.603972
C 3.123597 2.617229 -0.817792	C 3.014690 2.619924 -0.989267	C 3.126105 2.616848 -0.813348	C 3.123518 2.618810 -0.813372	C 3.121563 2.616099 -0.828171
C 2.332129 3.366637 0.298081	C 2.304845 3.385974 0.176623	C 2.333262 3.365505 0.301935	C 2.333042 3.364943 0.305514	C 2.332563 3.366527 0.289702
H 2.117155 4.399565 0.010080	H 2.035666 4.401634 -0.127113	H 2.119606 4.398956 0.014849	H 2.118360 4.398766 0.020495	H 2.116984 4.399148 0.001237
C 1.076317 2.545662 0.346129	C 1.074531 2.541708 0.355697	C 1.076940 2.545144 0.346514	C 1.076775 2.544520 0.351615	C 1.076631 2.545875 0.341029
C -0.170808 3.052232 0.684489	C -0.155307 3.028866 0.788558	C -0.170541 3.051622 0.680609	C -0.169289 3.049234 0.693774	C -0.169444 3.050892 0.683657
H -0.217759 4.093741 0.972089	H -0.199086 4.065927 1.091372	H -0.217798 4.093294 0.967240	H -0.215967 4.088715 0.988432	H -0.215999 4.091582 0.974081
C -1.367682 2.362139 0.570897	C -1.349835 2.335351 0.681303	C -1.367801 2.361291 0.565494	C -1.366094 2.359090 0.573764	C -1.366353 2.359212 0.574120
C -2.704317 2.999045 0.852616	C -2.702898 2.946234 0.946756	C -2.705307 2.998511 0.841704	C -2.705499 2.990559 0.853907	C -2.703221 2.992342 0.863443
C -3.646255 2.010176 0.098698	C -3.572397 2.046659 0.009955	C -3.642461 2.006575 0.088118	C -3.636296 2.010393 0.077429	C -3.642096 2.006797 0.103944
C -2.836939 0.694817 0.184311	C -2.809200 0.715160 0.098130	C -2.841255 0.695137 0.186734	C -2.837956 0.697400 0.173288	C -2.841079 0.694663 0.191171
C -3.314567 -0.588969 0.146763	C -3.294229 -0.556453 -0.051867	C -3.316566 -0.589952 0.154300	C -3.314739 -0.586585 0.139376	C -3.316401 -0.589788 0.152270
C -2.407158 -1.730586 0.035617	C -2.399439 -1.711276 -0.013852	C -2.406977 -1.730037 0.037973	C -2.407285 -1.730607 0.043073	C -2.406976 -1.729751 0.031661
C -2.804743 -3.196168 -0.244239	C -2.798835 -3.197579 -0.150051	C -2.804167 -3.195437 -0.248128	C -2.803806 -3.203368 -0.214169	C -2.803743 -3.193427 -0.262249
C -1.443931 -3.919140 0.015012	C -1.446992 -3.889969 0.235588	C -1.442575 -3.919057 0.004156	C -1.439872 -3.919583 0.044255	C -1.443146 -3.918674 -0.008893
H -1.359879 -4.075076 1.093387	H -1.392435 -3.928801 1.327528	H -1.356755 -4.081729 1.081327	H -1.346887 -4.061732 1.123757	H -1.360955 -4.086114 1.067743
C -0.434282 -2.848183 -0.381389	C -0.428898 -2.867927 -0.254230	C -0.433864 -2.845794 -0.386979	C -0.434126 -2.852065 -0.370158	C -0.431950 -2.844505 -0.391463
H -0.353635 -2.797900 -1.470214	H -0.377546 -2.898987 -1.345191	H -0.352004 -2.790886 -1.475519	H -0.360136 -2.812803 -1.459932	H -0.344057 -2.786969 -1.479336
C 0.935414 -3.080016 1.717311	C 1.006501 -3.075597 1.794156	C 0.932428 -3.082079 1.713373	C 0.949916 -3.075762 1.721134	C 0.917438 -3.082144 1.718136
H 0.796049 -4.147292 1.898427	H 0.789532 -4.127044 1.983513	H 0.792352 -4.149526 1.892807	H 0.811336 -4.142869 1.905313	H 0.787089 -4.151199 1.895939
H 0.102407 -2.552812 2.180173	H 0.221380 -2.503438 2.282142	H 0.099032 -2.555036 2.175839	H 0.122191 -2.546563 2.190553	H 0.072070 -2.565899 2.170941
H 1.848477 -2.763227 2.220491	H 1.953223 -2.824743 2.269581	H 1.844900 -2.766436 2.218314	H 1.867875 -2.759493 2.215915	H 1.819499 -2.756171 2.234063
N 1.377268 -1.387002 -0.058909	N 1.390002 -1.386511 -0.023531	N 1.377770 -1.386466 -0.059041	N 1.377253 -1.386342 -0.060345	N 1.378122 -1.385744 -0.054822
N 1.331827 1.291029 -0.017554	N 1.316899 1.289496 -0.016515	N 1.333063 1.290224 -0.016924	N 1.330563 1.291118 -0.019045	N 1.330902 1.291224 -0.023442
N -1.500524 1.069176 0.252250	N -1.480271 1.056503 0.307251	N -1.502393 1.068597 0.250889	N -1.499727 1.069897 0.245543	N -1.502235 1.068489 0.252224
N -1.121181 -1.604576 0.014727	N -1.113132 -1.599540 0.056803	N -1.121080 -1.604056 0.013932	N -1.121085 -1.605489 0.016061	N -1.121086 -1.603906 0.010168
C 2.520816 -4.927090 0.126315	C 2.535863 -4.942594 0.105297	C 2.518405 -4.927826 0.120890	C 2.531817 -4.922499 0.130172	C 2.501906 -4.933584 0.125779
H 1.677412 -5.622549 0.123964	H 1.754572 -5.683993 -0.076440	H 1.674259 -5.622377 0.116099	H 1.692911 -5.622997 0.135279	H 1.641498 -5.607244 0.129204
H 2.848283 -4.812257 1.159879	H 2.715712 -4.911948 1.179120	H 2.844480 -4.815244 1.155133	H 2.864341 -4.801359 1.161329	H 2.840766 -4.831845 1.157100
H 3.332515 -5.405393 -0.426823	H 3.445458 -5.315403 -0.374347	H 3.330392 -5.405883 -0.432018	H 3.343741 -5.398549 -0.424720	H 3.293820 -5.431371 -0.438293
C 1.793195 -3.817739 -2.014237	C 1.772436 -3.767142 -1.972509	C 1.795635 -3.812795 -2.018744	C 1.786097 -3.828796 -2.010609	C 1.814498 -3.791180 -2.011997
H 0.910665 -4.454248 -2.089601	H 0.912313 -4.436844 -2.039182	H 0.913070 -4.448720 -2.097587	H 0.897175 -4.457195 -2.076666	H 0.939575 -4.436041 -2.105121
H 1.551582 -2.860549 -2.485991	H 1.481482 -2.807616 -2.409280	H 1.555293 -2.854251 -2.488330	H 1.551546 -2.873729 -2.490633	H 1.565797 -2.829028 -2.469337
C 2.918680 -4.421031 -2.839881	C 2.895519 -4.298315 -2.848067	C 2.922337 -4.414247 -2.844065	C 2.902621 -4.453936 -2.832549	C 2.951506 -4.364980 -2.842394
O 3.878389 -3.754673 -3.224402	O 3.790284 -3.568874 -3.273236	O 3.881686 -3.746653 -3.227257	O 3.885010 -3.811808 -3.204480	O 3.891586 -3.672962 -3.229675
N 2.800733 -5.734719 -3.122813	N 2.853194 -5.616735 -3.128986	N 2.806054 -5.727872 -3.128035	N 2.749540 -5.760448 -3.131508	N 2.866822 -5.681131 -3.126641
H 3.525584 -6.185044 -3.664127	H 3.581939 -6.017921 -3.702674	H 3.531364 -6.176837 -3.669833	H 3.465556 -6.224598 -3.672916	H 3.599278 -6.111239 -3.674116
H 2.015066 -6.289792 -2.820109	H 2.117610 -6.221771 -2.797759	H 2.020077 -6.283658 -2.827496	H 1.944065 -6.295736 -2.845578	H 2.093787 -6.255366 -2.826986
C 4.305210 -2.563351 0.710300	C 4.240513 -2.604969 0.834987	C 4.303530 -2.566182 0.712827	C 4.315133 -2.565805 0.688603	C 4.271275 -2.566173 0.786139
H 3.793019 -2.782233 1.650155	H 4.419626 -3.650155 1.093115	H 3.789494 -2.785354 1.651589	H 3.814821 -2.791408 1.631560	H 3.735899 -2.812695 1.703021
H 4.709110 -1.554863 0.824427	H 3.731148 -2.169200 1.696480	H 4.708601 -1.558365 0.828595	H 4.707884 -1.546334 0.806923	H 4.650629 -1.552053 0.943290
C 5.505038 -3.490245 0.538535	C 5.586436 -1.891010 0.687198	C 5.502336 -3.494628 0.542070	C 5.524895 -3.468900 0.497984	C 5.474581 -3.487914 0.636542
H 5.873435 -3.431128 -0.493454	H 6.129064 -1.978619 1.635871	H 5.872152 -3.435405 -0.489384	H 5.870458 -3.412205 -0.541912	H 6.057936 -3.215652 -0.250457
H 5.247980 -4.536701 0.719213	H 5.451420 -0.824653 0.504526	H 5.243591 -4.540831 0.721768	H 5.287166 -4.517806 0.691347	H 5.167963 -4.528577 0.490727
C 6.655823 -3.056006 1.436056	C 6.499834 -2.411142 -0.410831	C 6.652638 -3.062577 1.441221	C 6.688127 -3.008898 1.366284	C 6.369598 -3.432828 1.865473
N 7.449244 -4.049545 1.889839	N 6.595798 -3.757721 -0.525707	N 7.443244 -4.057692 1.896507	N 7.503297 -3.985686 1.817122	N 7.577857 -4.022522 1.721715
H 8.273369 -3.812557 2.423784	H 7.272659 -4.129757 -1.177282	H 8.266850 -3.822380 2.432001	H 8.333413 -3.731492 2.333587	H 8.195477 -4.074358 2.518997
H 7.303459 -5.014999 1.637551	H 6.235236 -4.385225 0.176815	H 7.295348 -5.022979 1.644864	H 7.361650 -4.957007 1.586007	H 7.847667 -4.498353 0.874135
O 6.860722 -1.876313 1.708405	O 7.115682 -1.652042 -1.151925	O 6.859779 -1.883255 1.713377	O 6.884015 -1.822752 1.617290	O 6.021156 -2.897802 2.913322
C 4.492427 -0.122339 -1.662840	C 4.282284 -0.119860 -1.980173	C 4.496146 -0.122437 -1.657673	C 4.489899 -0.117750 -1.668642	C 4.488838 -0.132099 -1.665462
H 4.409878 -0.913753 -2.413154	H 5.257411 -0.200167 -1.495486	H 4.414483 -0.913043 -2.408937	H 4.741441 0.792294 -2.202457	H 5.331145 -0.395519 -1.015989
H 4.744583 0.785679 -2.199936	H 4.154641 -1.017794 -2.592283	H 4.749594 0.785982 -2.193461	H 5.333677 -0.374070 -1.018237	H 4.396120 -0.921592 -2.416828
H 5.335580 -0.376041 -1.010538	H 4.322705 0.720774 -2.666704	H 5.338128 -0.377160 -1.004280	H 4.407036 -0.905707 -2.422444	H 4.747567 0.775712 -2.199910
C 4.632441 2.847903 -0.696061	C 4.527265 2.843631 -0.989504	C 4.634816 2.847226 -0.689471	C 4.632502 2.847860 -0.691061	C 4.630564 2.847100 -0.709739
H 4.810622 3.904527 -0.474901	H 4.730139 3.913008 -0.876858	H 4.812794 3.903541 -0.466653	H 4.811618 3.904063 -0.468884	H 4.808853 3.904334 -0.491858
H 5.071159 2.252240 0.107102	H 5.010147 2.315165 -0.164809	H 5.072524 2.250454 0.113399	H 5.070363 2.251219 0.111795	H 5.071353 2.254045 0.094153
H 5.154150 2.630252 -1.624619	H 4.986319 2.529493 -1.923152	H 5.157643 2.630763 -1.617695	H 5.154430 2.630671 -1.619562	H 5.150603 2.626800 -1.638509
C 2.588264 3.137602 -2.185763	C 2.361113 3.131055 -2.308786	C 2.592790 3.138135 -2.181731	C 2.588561 3.144490 -2.179469	C 2.584878 3.134364 -2.196193
H 1.494886 3.089934 -2.191743	H 1.273372 3.034550 -2.233824	H 1.499384 3.091324 -2.188950	H 1.495142 3.099012 -2.184831	H 1.491621 3.084873 -2.201820
H 2.878798 4.187631 -2.276837	H 2.596497 4.195051 -2.399425	H 2.884313 4.187943 -2.272215	H 2.880775 4.194321 -2.267435	H 2.873657 4.184756 -2.288691
C 3.157056 2.417222 -3.396594	C 2.865586 2.471038 -3.580580	C 3.162305 2.417481 -3.392020	C 3.154906 2.426639 -3.393058	C 3.155299 2.412395 -3.405607
O 4.290342 2.660306 -3.811837	O 3.960809 2.763934 -4.060528	O 4.295949 2.660159 -3.806458	O 4.287674 2.669742 -3.809631	O 4.288040 2.657052 -3.821497
N 2.358890 1.500077 -3.976969	N 2.052531 1.557954 -4.146068	N 2.364220 1.500563 -3.972914	N 2.354888 1.511861 -3.974811	N 2.359409 1.491788 -3.983485
H 2.742534 0.962896 -4.742622	H 2.400484 1.062212 -4.955537	H 2.748540 0.962778 -4.737804	H 2.737408 0.975806 -4.741817	H 2.744407 0.953271 -4.747526
H 1.459792 1.212103 -3.602847	H 1.197877 1.216404 -3.715457	H 1.465034 1.212534 -3.599042	H 1.457106 1.221788 -3.599269	H 1.460734 1.202973 -3.608886
C 3.059145 3.353963 1.659611	C 3.138298 3.455107 1.472260	C 3.057933 3.350368 1.664658	C 3.061180 3.348377 1.666267	C 3.065144 3.353399 1.649027
H 3.483974 2.364421 1.835546	H 3.601146 2.486423 1.662265	H 3.482062 2.360364 1.839745	H 3.480477 2.356378 1.842090	H 3.499734 2.367132 1.817692
H 3.900654 4.047405 1.615500	H 3.953765 4.166307 1.328533	H 3.899793 4.043561 1.623307	H 3.906423 4.037162 1.620861	H 3.900637 4.054066 1.603830
C 2.182934 3.714185 2.851425	C 2.335983 3.855583 2.703650	C 2.179567 3.708824 2.855395	C 2.187824 3.715149 2.858202	C 2.193195 3.697496 2.849041
H 1.371869 2.988798 2.973302	H 1.568722 3.106984 2.927260	H 1.367493 2.984016 2.973794	H 1.369228 2.998049 2.978559	H 1.374556 2.978172 2.955568
H 1.703203 4.690516 2.710598	H 1.804312 4.800574 2.537284	H 1.701107 4.685936 2.715666	H 1.718029 4.696402 2.717750	H 1.723770 4.681733 2.730252
C 2.979223 3.753494 4.146264	C 3.211621 4.014788 3.935509	C 2.973170 3.744608 4.151958	C 2.983454 3.745538 4.153773	C 2.990835 3.698691 4.144364
O 4.202030 3.651446 4.183107	O 4.438019 3.969562 3.897409	O 4.196003 3.643787 4.190984	O 4.205309 3.632967 4.191504	O 4.212402 3.585333 4.178391
N 2.238748 3.940589 5.264511	N 2.536803 4.242745 5.087146	N 2.230241 3.927526 5.269374	N 2.244081 3.937460 5.272001	N 2.251831 3.864622 5.267179
H 2.698184 3.863461 6.160965	H 3.056637 4.254008 5.953253	H 2.688055 3.846335 6.166306	H 2.702602 3.853213 6.168315	H 2.710969 3.762435 6.161266
H 1.232890 3.860010 5.247115	H 1.536686 4.120701 5.144154	H 1.224702 3.843438 5.249787	H 1.237666 3.864143 5.254478	H 1.245534 3.792375 5.248733
C -2.824167 4.454784 0.406732	C -2.793103 4.443547 0.662791	C -2.813912 4.454220 0.389789	C -2.813242 4.453336 0.425521	C -2.816080 4.452475 0.427133
H -2.460920 4.615590 -0.610157	H -2.360886 4.718825 -0.301275	H -2.453511 4.608691 -0.629192	H -2.445690 4.624634 -0.588225	H -2.455938 4.619008 -0.590074
H -3.871922 4.764786 0.459237	H -3.838659 4.767614 0.681507	H -3.854918 4.788598 0.445576	H -3.854993 4.786069 0.479072	H -3.857846 4.783858 0.486052
H -2.255329 5.106777 1.074745	H -2.269939 5.007956 1.439318	H -2.236028 5.101947 1.053877	H -2.240842 5.090943 1.104071	H -2.239897 5.094469 1.098187
C -2.953513 2.925433 2.370972	C -3.080757 2.688568 2.417082	C -2.966093 2.920670 2.356857	C -2.977650 2.887652 2.366596	C -2.957293 2.897708 2.379075
H -3.912648 3.391460 2.614583	H -2.348744 3.162832 3.077000	H -3.935052 3.367240 2.590574	H -3.944517 3.338599 2.600170	H -2.178029 3.443954 2.918060
H -2.952261 1.904004 2.759409	H -4.064826 3.114180 2.624673	H -2.973672 1.895575 2.729708	H -2.998682 1.856147 2.723292	H -3.924908 3.341804 2.622924

H -2.171005 3.474087 2.901747	H -3.120757 1.625445 2.661323	H -2.189453 3.472831 2.893847	H -2.200983 3.425030 2.918220	H -2.963104 1.868432 2.740752
C -3.779276 2.393798 -1.385043	C -3.511341 2.558791 -1.445117	C -3.766885 2.376107 -1.405033	C -3.740216 2.394379 -1.413836	C -3.767997 2.390527 -1.386894
H -4.274938 3.365808 -1.431142	H -3.994837 3.539022 -1.480816	H -4.245310 3.357578 -1.475351	H -4.215403 3.377406 -1.481753	H -4.247685 3.371056 -1.446430
H -2.781305 2.524325 -1.812295	H -2.463528 2.712580 -1.723397	H -2.767809 2.490668 -1.835503	H -2.735483 2.509872 -1.830700	H -2.765391 2.500591 -1.813374
C -4.562799 1.406183 -2.237304	C -4.179763 1.643352 -2.472832	C -4.565109 1.384978 -2.238720	C -4.530113 1.412478 -2.266497	C -4.578721 1.400727 -2.225896
H -4.109359 0.410272 -2.226424	H -3.708292 0.661118 -2.495795	H -4.106548 0.391775 -2.236146	H -4.087235 0.412118 -2.248592	H -4.122546 0.410971 -2.230884
H -5.585530 1.285202 -1.870854	H -5.237494 1.518366 -2.224461	H -5.577035 1.257497 -1.844158	H -5.554345 1.301657 -1.899506	H -5.588688 1.314646 -1.814514
C -4.625316 1.851508 -3.675729	C -4.076646 2.218007 -3.862093	C -4.671150 1.830069 -3.674487	C -4.590212 1.854123 -3.706056	C -4.686460 1.847100 -3.661195
O -4.066974 2.823149 -4.136947	O -3.523031 1.685793 -4.799022	O -4.174103 2.833076 -4.138584	O -4.062421 2.845728 -4.160715	O -4.324918 1.203180 -4.621540
O -5.378760 1.021565 -4.406081	O -4.669876 3.415362 -3.945658	O -5.389639 0.963437 -4.396854	O -5.305439 0.997557 -4.443715	O -5.237423 3.063352 -3.760472
C -4.805274 -0.860629 0.156203	C -4.754642 -0.799158 -0.369639	C -4.804924 -0.869678 0.176862	C -4.803592 -0.859043 0.158947	C -4.804664 -0.869330 0.171982
H -5.113156 -1.460173 -0.702485	H -5.320751 0.122844 -0.310066	H -5.122862 -1.445802 -0.694664	H -5.112462 -1.488320 -0.677804	H -5.121199 -1.440539 -0.703318
H -5.369695 0.063531 0.119723	H -5.217813 -1.501558 0.321854	H -5.370844 0.053215 0.188114	H -5.366926 0.063616 0.103057	H -5.371091 0.053179 0.187176
H -5.113643 -1.397862 1.057645	H -4.877849 -1.203570 -1.377381	H -5.094165 -1.434567 1.066997	H -5.098548 -1.359946 1.085223	H -5.095201 -1.438911 1.058650
C -3.212155 -3.299898 -1.728368	C -3.185916 -3.515672 -1.604846	C -3.214582 -3.289822 -1.732005	C -3.223656 -3.325672 -1.693168	C -3.209426 -3.279338 -1.747906
H -4.086055 -2.682218 -1.941243	H -4.075954 -2.964892 -1.908171	H -4.090843 -2.673024 -1.937637	H -4.106956 -2.720919 -1.903713	H -4.086987 -2.663725 -1.951538
H -3.466616 -4.336347 -1.962789	H -3.406737 -4.582722 -1.691400	H -3.466972 -4.325200 -1.973427	H -3.465803 -4.366939 -1.918776	H -3.458329 -4.313703 -1.997300
H -2.409281 -2.979540 -2.399012	H -2.384238 -3.271571 -2.308422	H -2.414178 -2.962492 -2.402264	H -2.430140 -2.998858 -2.371753	H -2.408205 -2.944613 -2.413439
C -3.931022 -3.772834 0.627127	C -3.927719 -3.647329 0.800849	C -3.927198 -3.782563 0.620355	C -3.919559 -3.775152 0.675951	C -3.930093 -3.784785 0.599070
H -4.885045 -3.343853 0.329832	H -4.903311 -3.484182 0.337590	H -4.883374 -3.355347 0.327858	H -4.862535 -3.295233 0.427705	H -4.885134 -3.355561 0.305825
H -4.012460 -4.837622 0.398435	H -3.843228 -4.729757 0.911519	H -4.004686 -4.845634 0.382628	H -4.051061 -4.825670 0.410292	H -4.007324 -4.846519 0.355519
C -3.763863 -3.594760 2.137430	C -3.882760 -2.973711 2.176228	C -3.758551 -3.618012 2.131866	C -3.681612 -3.662070 2.189349	C -3.765568 -3.628118 2.111654
H -2.960962 -4.225830 2.525022	H -2.845574 -2.851322 2.502268	H -2.950107 -4.246542 2.512008	H -3.014794 -4.457578 2.533484	H -2.960105 -4.261110 2.490728
H -3.504417 -2.562716 2.390944	H -4.332955 -1.980456 2.140772	H -3.505963 -2.586558 2.395186	H -3.208125 -2.708982 2.439786	H -3.511244 -2.598469 2.380599
C -5.077979 -3.919845 2.820206	C -4.575953 -3.740261 3.284638	C -5.068268 -3.960471 2.814928	C -5.014921 -3.710772 2.906055	C -5.077585 -3.968776 2.790697
O -6.109939 -3.309869 2.534992	O -5.489667 -3.261721 3.955787	O -6.109637 -3.368955 2.525454	O -5.810473 -2.770271 2.834759	O -6.116161 -3.371766 2.502078
N -5.042016 -4.954181 3.690217	N -4.067524 -4.979721 3.501585	N -5.016364 -4.987739 3.692574	N -5.312657 -4.854513 3.551272	N -5.031195 -4.998559 3.665625
H -4.134624 -5.163678 4.104545	H -3.135198 -5.129140 3.125653	H -4.105841 -5.181925 4.107893	H -4.586761 -5.540497 3.691140	H -4.121770 -5.199531 4.080344
C -1.233168 -5.262758 -0.691360	C -1.196023 -5.296201 -0.312905	C -1.231841 -5.258088 -0.710984	C -1.232031 -5.271966 -0.645365	C -1.232053 -5.254330 -0.730024
H -1.307938 -5.156359 -1.774155	H -1.230941 -5.311444 -1.403155	H -1.308407 -5.144835 -1.792969	H -1.305726 -5.178870 -1.729421	H -1.304676 -5.136051 -1.811750
H -0.226213 -5.621303 -0.463954	H -0.190764 -5.596664 -0.009058	H -0.224125 -5.616942 -0.487535	H -0.226198 -5.630128 -0.412146	H -0.225867 -5.615726 -0.504760
C -2.228764 -6.330396 -0.273427	C -2.164344 -6.356457 0.182124	C -2.224554 -6.330561 -0.298713	C -2.232333 -6.330256 -0.214759	C -2.226966 -6.327343 -0.324456
O -3.141055 -6.696651 -1.007605	O -3.005429 -6.861336 -0.556886	O -3.135516 -6.695837 -1.034999	O -3.144445 -6.703043 -0.945334	O -3.133123 -6.692520 -1.066650
N -2.068567 -6.809386 0.980576	N -2.022569 -6.713592 1.479386	N -2.062781 -6.816134 0.952560	N -2.080500 -6.789347 1.048891	N -2.073315 -6.813918 0.927632
H -2.698358 -7.525686 1.314081	H -2.708890 -7.353515 1.856723	H -2.690991 -7.535393 1.282570	H -2.700204 -7.516348 1.378826	H -2.704452 -7.532811 1.252800
H -1.267244 -6.580678 1.549597	H -1.480354 -6.164689 2.145289	H -1.263082 -6.586945 1.523644	H -1.265352 -6.577408 1.604845	H -1.279143 -6.583908 1.506200
O -4.951087 2.022844 0.624576	O -4.903028 1.953998 0.458648	O -4.913241 1.937352 0.694246	O -4.914578 1.939624 0.666733	O -4.912688 1.933805 0.708065
H -4.931411 1.723326 1.546511	H -5.346581 2.795203 0.267325	H -5.380938 2.765349 0.502765	H -5.367018 2.781248 0.498987	H -5.368005 2.776240 0.551687
C -5.488385 1.343145 -5.797622	C -4.604399 4.055470 -5.225579	C -5.542358 1.286343 -5.784128	C -5.415412 1.315794 -5.836013	C -5.371286 3.581181 -5.089319
H -6.124688 0.573207 -6.230547	H -5.120913 5.006407 -5.108169	H -6.144053 0.485497 -6.210282	H -4.427128 1.344225 -6.299620	H -5.825893 4.564158 -4.979842
H -5.940183 2.328818 -5.926980	H -3.564619 4.218126 -5.516426	H -6.048894 2.246866 -5.898754	H -6.018655 0.522772 -6.274476	H -4.392296 3.663601 -5.565711
H -4.503720 1.334528 -6.269732	H -5.098013 3.443159 -5.982913	H -4.567528 1.333418 -6.273919	H -5.902875 2.283850 -5.968764	H -6.009590 2.929401 -5.689190
C -6.199142 -5.296220 4.492162	C -4.325210 -5.678502 4.745131	C -6.167953 -5.339795 4.498076	C -6.565237 -5.020553 4.263247	C -6.184752 -5.344285 4.471078
H -6.125960 -6.349479 4.778950	H -3.946363 -6.699800 4.653639	H -6.081825 -6.390553 4.790439	H -6.684067 -6.081761 4.494720	H -6.109593 -6.397738 4.756602
H -7.092645 -5.163951 3.878651	H -5.404113 -5.725365 4.909586	H -7.063796 -5.221433 3.885210	H -7.385642 -4.716191 3.607747	H -7.080282 -5.211930 3.860720
C -6.320596 -4.440309 5.747949	C -3.686355 -4.989153 5.947042	C -6.298083 -4.479076 5.749688	C -6.623595 -4.202035 5.544021	C -6.301647 -4.490232 5.728459
H -6.349235 -3.388000 5.456565	H -4.045498 -3.957931 5.955431	H -6.334412 -3.428262 5.453511	H -6.479147 -3.151854 5.284774	H -6.321600 -3.436889 5.439497
C -7.550355 -4.798790 6.558959	C -4.052352 -5.673250 7.249545	C -7.526333 -4.843366 6.560424	C -7.931519 -4.377370 6.290582	C -7.535111 -4.840981 6.537294
H -8.457219 -4.634953 5.969683	H -5.136273 -5.663727 7.393428	H -8.433557 -4.688407 5.969305	H -8.778526 -4.060437 5.674278	H -8.440115 -4.667896 5.947826
H -7.516086 -5.850392 6.860833	H -3.708209 -6.712014 7.243209	H -7.484783 -5.893484 6.866453	H -8.074532 -5.425946 6.570693	H -7.509489 -5.893772 6.835851
H -7.602954 -4.181712 7.459189	H -3.583992 -5.158019 8.091619	H -7.584600 -4.223197 7.458158	H -7.909703 -3.770241 7.198184	H -7.583265 -4.226349 7.439453
C -1.625260 -0.050113 5.196253	C -1.401902 0.212359 5.364937	C -1.627821 -0.059208 5.195634	C -1.637373 -0.124804 5.194327	C -1.589444 -0.116336 5.214382
H -1.210362 0.457347 6.070169	H -1.138012 1.157689 5.847071	H -1.215112 0.449390 6.070010	H -1.289804 0.461121 6.049150	H -1.158181 0.369949 6.092652
C -1.998596 -1.507098 5.517344	C -1.302987 -0.980565 6.338784	C -1.994009 -1.518255 5.515843	C -1.792105 -1.610738 5.563598	C -1.969834 -1.575693 5.513424
H -1.372167 -1.906937 6.322248	H -1.675940 -0.605549 7.297239	H -1.365554 -1.915779 6.320307	H -1.142123 -1.890307 6.399236	H -1.329770 -1.997483 6.296153
C -3.462389 -1.321151 5.945733	C -2.394033 -1.921296 5.815091	C -3.458567 -1.338992 5.944572	C -3.274004 -1.658722 5.901861	C -3.422062 -1.383086 5.976612
H -3.501318 -0.906511 6.958831	H -2.775352 -2.548874 6.621295	H -3.499135 -0.923636 6.957338	H -3.440928 -1.213944 6.885712	H -3.434263 -0.985100 6.997233
C -3.946300 -0.279331 4.948158	C -3.444491 -0.945834 5.288468	C -3.946970 -0.300386 4.946040	C -3.871855 -0.731306 4.851603	C -3.916043 -0.319808 5.006697
H -4.181434 -0.770720 3.996548	H -3.902855 -1.342235 4.383346	H -4.178068 -0.793392 3.994454	H -3.923353 -1.237737 3.882625	H -4.177081 -0.793479 4.053095
C -5.115724 0.563956 5.411548	C -4.554461 -0.623423 6.262768	C -5.121686 0.537228 5.405758	C -5.255381 -0.196150 5.185819	C -5.065895 0.528251 5.508518
H -5.403018 1.258492 4.609477	H -5.151251 0.197858 5.844280	H -5.410097 1.228195 4.601322	H -5.425287 0.707801 4.590948	H -5.363328 1.236558 4.722735
H -5.966603 -0.091335 5.616240	H -5.199421 -1.509926 6.337537	H -5.969498 -0.122183 5.610002	H -5.977310 -0.950862 4.846592	H -5.918747 -0.121103 5.723690
O -2.817851 0.596354 4.791574	O -2.725277 0.260037 4.933808	O -2.823084 0.581545 4.791547	O -2.905954 0.335960 4.786712	O -2.781977 0.546716 4.838489
O -1.898239 -2.275875 4.347826	O -0.020732 -1.489086 6.504302	O -1.890328 -2.285939 4.345748	O -1.529689 -2.409174 4.427751	O -1.901364 -2.321615 4.327104
H -2.185216 -3.192667 4.599114	H 0.060631 -2.390532 6.099435	H -2.172748 -3.204041 4.596901	H -2.038658 -3.228532 4.555652	H -2.188830 -3.240574 4.569647
O -4.811320 1.253666 6.606583	O -3.993701 -0.273139 7.513831	O -4.823220 1.230649 6.600432	O -5.433236 0.129905 6.542874	O -4.726932 1.197793 6.706019
H -3.998775 1.754309 6.429237	H -4.714616 -0.094606 8.134016	H -4.012170 1.733854 6.423726	H -5.530396 -0.729822 7.018910	H -3.911841 1.689887 6.516963
O -4.278594 -2.464779 5.845708	O -1.976794 -2.722966 4.707285	O -4.269354 -2.486825 5.846194	O -3.783559 -2.970838 5.821526	O -4.253422 -2.516021 5.876071
P -3.760846 -3.881526 6.508767	P -1.255545 -4.186327 4.882207	P -3.742970 -3.899655 6.510431	P -4.648840 -3.520710 7.138411	P -3.740256 -3.950864 6.501723
O -3.231329 -3.661045 7.885193	O 0.043896 -4.006721 5.616853	O -3.213088 -3.674679 7.885979	O -5.515341 -2.371292 7.592923	O -3.204476 -3.769996 7.881504
O -2.861885 -4.536042 5.467930	O -1.251041 -4.795070 3.503685	O -2.841638 -4.550648 5.469401	O -3.740214 -4.225783 8.086662	O -2.850901 -4.586778 5.441122
O -5.181157 -4.654233 6.601537	O -2.246454 -4.986161 5.891222	O -5.158721 -4.680132 6.606459	O -5.538137 -4.639495 6.376377	O -5.165453 -4.715637 6.583083
N -0.656805 0.061842 4.130049	N -0.502541 0.079441 4.212591	N -0.659507 0.057809 4.129613	N -0.683688 0.072536 4.119055	N -0.633775 0.009636 4.138467
C -0.940468 -0.126852 2.827057	C -0.849930 -0.143772 2.930937	C -0.942949 -0.126061 2.825979	C -0.959179 -0.121053 2.816104	C -0.934599 -0.144228 2.834151
H -1.946961 -0.273167 2.475922	H -1.867239 -0.326371 2.627513	H -1.949696 -0.266925 2.473593	H -1.964116 -0.266590 2.459417	H -1.945156 -0.282823 2.491261
N 0.131003 -0.102010 2.067779	N 0.182965 -0.097869 2.111794	N 0.129226 -0.101862 2.067446	N 0.116978 -0.096975 2.061855	N 0.128143 -0.099736 2.063307
C 2.588500 0.114167 2.708602	C 2.657936 0.269186 2.619582	C 2.586657 0.109748 2.710234	C 2.570924 0.113379 2.715755	C 2.589013 0.118514 2.674614
H 2.992845 -0.001905 1.714489	H 3.023196 0.140107 1.612370	H 2.991498 -0.003612 1.716024	H 2.980544 -0.006006 1.723971	H 2.976213 0.043434 1.671111
C 3.442480 0.336127 3.777509	C 3.546011 0.581648 3.635784	C 3.440207 0.328542 3.780170	C 3.418947 0.328882 3.790694	C 3.459606 0.307276 3.734658
C 2.924071 0.515136 5.089149	C 3.081969 0.786933 4.964663	C 2.921269 0.504122 5.091996	C 2.892922 0.510966 5.099200	C 2.962866 0.429343 5.060753
C 1.560725 0.420603 5.324520	C 1.740710 0.628794 5.267627	C 1.557733 0.409638 5.326330	C 1.527700 0.423038 5.325968	C 1.602999 0.326471 5.314160
H 1.166945 0.537505 6.328487	H 1.384704 0.731302 6.284854	H 1.163423 0.524299 6.330346	H 1.127198 0.542223 6.327025	H 1.225776 0.406025 6.328078
C 0.721862 0.189880 4.237195	C 0.866185 0.299473 4.233187	C 0.719278 0.182773 4.237929	C 0.694947 0.196433 4.232955	C 0.746291 0.134299 4.232381
C 1.212800 0.069323 2.932448	C 1.295885 0.157689 2.910795	C 1.210820 0.065243 2.933123	C 1.193594 0.075311 2.931411	C 1.218760 0.053471 2.918499
C 4.924425 0.440747 3.534069	C 5.010391 0.743447 3.325662	C 4.922248 0.433910 3.537688	C 4.903421 0.422119 3.558223	C 4.937444 0.410473 3.467246
H 5.495250 -0.188653 4.224052	H 5.331709 1.768481 3.533758	H 5.186277 0.127125 2.522899	H 5.173657 0.102854 2.548815	H 5.139812 0.380612 2.392995
H 5.188138 0.130635 2.520186	H 5.628831 0.069752 3.928619	H 5.248282 1.468687 3.688339	H 5.234940 1.456356 3.700179	H 5.330692 1.353064 3.858117
H 5.250549 1.475979 3.681321	H 5.214696 0.533650 2.273594	H 5.492873 -0.197694 4.225803	H 5.464169 -0.205000 4.258493	H 5.489608 -0.417101 3.924318
C 3.858906 0.850569 6.219176	C 4.056072 1.189982 6.038630	C 3.855551 0.836801 6.223315	C 3.822285 0.842290 6.234939	C 3.918898 0.716692 6.185818
H 4.391612 1.782342 5.998139	H 4.554873 2.127482 5.769120	H 4.387494 1.769734 6.005253	H 4.367608 1.766453 6.012960	H 4.715044 -0.033012 6.242785
H 4.614623 0.071829 6.367785	H 4.839576 0.436724 6.176566	H 4.611964 0.058379 6.369848	H 4.567631 0.055759 6.394686	H 3.403999 0.741283 7.149300
H 3.315131 0.977590 7.158539	H 3.550450 1.331027 6.997019	H 3.311392 0.960526 7.162899	H 3.272112 0.981148 7.168854	H 4.400618 1.688237 6.025565

References

(1) Proinsias, K.; Sessler, J.L.; Kurcoń, S.; Gryko, D. New Hydrophobic Vitamin B_{12} Derivatives via Ring-Opening Reactions of *c*-Lactone. *Org. Lett.* **2010**, *12* (20), 4674–4677.

(2) Bonnett, R.; Neuberger, A.; Kenner, G.W. Neovitamin B_{12} (Cyano-13-Epicobalamin). *Philos. Trans. R. Soc.* **1997**, *273* (924), 295–301.

(3) Bonnett, R.; Godfrey, J.M.; Math, V.B. Cyano-13-Epicobalamin (Neovitamin B_{12}) and Its Relatives. *J .Chem. Soc. C.* **1971**, 3736–3743.

(4) Unno, M.; Kikukawa, T.; Kumauchi, M.; Kamo, N. Exploring the Active Site Structure of a Photoreceptor Protein by Raman Optical Activity. *J. Phys. Chem.* **2013**, *117* (5), 1321–1325.

(5) Haraguchi, S.; Hara, M.; Shingae, T.; Kumauchi, M.; Hoff, W. D.; Unno, M. Experimental Detection of the Intrinsic Difference in Raman Optical Activity of a Photoreceptor Protein under Preresonance and Resonance Conditions. *Angew. Chem. Int. Ed.* **2015**, *54* (39), 11555–11558.

(6) Prieto, L.; Neuburger, M.; Spingler, B.; Zelder, F. Inorganic Cyanide as Protecting Group in the Stereospecific Reconstitution of Vitamin B_{12} from an Artificial Green Secocorrinoid. *Org. Lett.* **2016**, *18* (20), 5292–5295.

(7) Wierzba, A.J.; Wincenciuk, A.; Karczewski, M.; Vullev, V.I.; Gryko, D. Meso-Modified Cobalamins: Synthesis, Structure, and Properties. *Chem. Eur. J.* **2018**, *24* (41), 10344–10356.

(8) Allouche, A.R. Gabedit—A Graphical User Interface for Computational Chemistry Softwares. *J. Comput. Chem.* **2011**, *32* (1), 174–182.

(9) Rappe, A.K.; Casewit, C.J.; Colwell, K.S.; Goddard, W.A.; Skiff, W.M. UFF, a Full Periodic Table Force Field for Molecular Mechanics and Molecular Dynamics Simulations. *J. Am. Chem. Soc.* **1992**, *114* (25), 10024–10035.

(10) Becke, A.D. Density-Functional Thermochemistry. III. The Role of Exact Exchange. *J. Chem. Phys.* **1993**, *98* (7), 5648.

(11) Lee, C.; Yang, W.; Parr, R.G. Development of the Colle-Salvetti Correlation-Energy Formula into a Functional of the Electron Density. *Phys. Rev. B* **1988**, *37* (2), 785–789.

(12) Stephens, P.J.; Devlin, F.J.; Chabalowski, C.F.; Frisch, M.J. Ab Initio Calculation of Vibrational Absorption and Circular Dichroism Spectra Using Density Functional Force Fields. *J. Phys. Chem.* **1994**, *98* (45), 11623–11627.

(13) Vosko, S. H.; Wilk, L.; Nusair, M. Accurate Spin-Dependent Electron Liquid Correlation Energies for Local Spin Density Calculations: A Critical Analysis. *Can. J. Phys.* **1980**, *58* (8), 1200–1211.

(14) Yanai, T.; Tew, D.P.; Handy, N.C. A New Hybrid Exchange–Correlation Functional Using the Coulomb-Attenuating Method (CAM-B3LYP). *Chem. Phys. Lett.* **2004**, *393* (1), 51–57.

(15) Gaussian 16, Revision C.01, M. J. Frisch, G. W. Trucks, H. B. Schlegel, G. E. Scuseria, M. A. Robb, J. R. Cheeseman, G. Scalmani, V. Barone, G. A. Petersson, H. Nakatsuji, X. Li, M. Caricato, A. V. Marenich, J. Bloino, B. G. Janesko, R. Gomperts, B. Mennucci, H.P. Hratchian, J. V. Ortiz, A. F. Izmaylov, J. L. Sonnenberg, D. Williams-Young, F. Ding, F. Lipparini, F. Egidi, J. Goings, B. Peng, A. Petrone, T. Henderson, D. Ranasinghe, V. G. Zakrzewski, J. Gao, N. Rega, G. Zheng, W. Liang, M. Hada, M. Ehara, K. Toyota, R. Fukuda, J. Hasegawa, M. Ishida, T. Nakajima, Y. Honda, O. Kitao, H. Nakai, T. Vreven, K. Throssell, J. A. Montgomery, Jr., J. E. Peralta, F. Ogliaro, M. J. Bearpark, J. J. Heyd, E. N. Brothers, K. N. Kudin, V. N. Staroverov, T. A. Keith, R. Kobayashi, J. Normand, K. Raghavachari, A. P. Rendell, J. C. Burant, S. S. Iyengar, J. Tomasi, M. Cossi, J. M. Millam, M. Klene, C. Adamo, R. Cammi, J. W. Ochterski, R. L. Martin, K. Morokuma, O. Farkas, J. B. Foresman, and D. J. Fox, Gaussian, Inc., Wallingford CT, **2019**.

(16) Grimme, S.; Antony, J.; Ehrlich, S.; Krieg, H.A Consistent and Accurate Ab Initio Parametrization of Density Functional Dispersion Correction (DFT-D) for the 94 Elements H-Pu. *J. Chem. Phys.* **2010**, *132* (15).

(17) Scalmani, G.; Frisch, M.J. Continuous Surface Charge Polarizable Continuum Models of Solvation. I. General Formalism. *J. Chem. Phys.* **2010**, *132* (11), 114110.

(18) Polavarapu, P.L.; Covington, C.L. Comparison of Experimental and Calculated Chiroptical Spectra for Chiral Molecular Structure Determination. *Chirality* **2014**, *26* (9), 539–552.

(19) Covington, C.L.; Polavarapu, P.L. Similarity in Dissymmetry Factor Spectra: A Quantitative Measure of Comparison between Experimental and Predicted Vibrational Circular Dichroism. *J. Phys. Chem. A* **2013**, *117* (16), 3377–3386.
